# Synthesis of Stable Betaines Based on 1*H*-Pyrrole-2,3-diones and Pyridinium Ylides and Their Thermal Conversion to Cyclopropane-Fused Pyrroles

**DOI:** 10.3390/molecules30234552

**Published:** 2025-11-26

**Authors:** Maria M. Muranova, Andrey R. Galeev, Ivan G. Mokrushin, Andrey N. Maslivets, Maksim V. Dmitriev

**Affiliations:** Department of Chemistry, Perm State University, ul. Bukireva 15, Perm 614990, Russiakoh2@psu.ru (A.N.M.)

**Keywords:** pyridinium ylides, azomethine ylides, pyrrole-2,3-diones, betaines, cyclopropanes, Michael addition

## Abstract

Pyridinium ylides, along with related azaheterocyclic ylides, are widely used in synthetic organic chemistry. However, reactions that yield stable zwitterionic adducts from these ylides remain underexplored. In this work, we demonstrate that the reaction of pyrrole-2,3-diones with in situ-generated pyridine-based azomethine ylides affords stable zwitterionic adducts, which are typically transient species in analogous processes. These betaines are used as key intermediates for the synthesis of cyclopropane-fused pyrroles or pyridine-2,3-diones via thermolysis in chlorobenzene.

## 1. Introduction

**Scheme 1:**
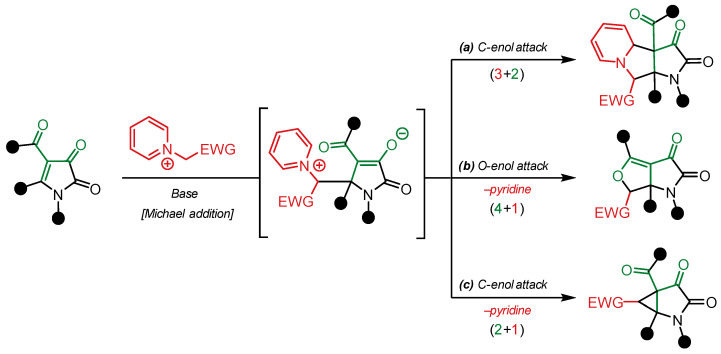
Main pathways for the reaction of Michael acceptors (exemplified on 1*H*-pyrrole-2,3-dione) with pyridinium ylides.

Pyridinium ylides (and related azaheterocyclic ylides) are widely used in synthetic organic chemistry as readily accessible C-nucleophiles and 1,3-dipoles [1,2,3,4,5,6]. Their reactions with α,β-unsaturated carbonyl compounds typically proceed via initial Michael addition to the activated double bond, generating an intermediate zwitterion, which can undergo further transformations via three major pathways (Scheme 1): (a) cyclization at the pyridine moiety to form dihydroindolizines [6,7,8]; (b) enolate attack, where pyridine acts as a leaving group, leading to dihydrofurans [9,10,11,12]; or (c) cyclopropane formation [13,14,15]. However, reactions yielding stable zwitterionic Michael adducts remain rare [16,17].

1*H*-Pyrrole-2,3-diones are cyclic α,β-unsaturated carbonyl compounds that serve as highly reactive Michael acceptors [18,19,20]. Recently, we developed an efficient cyclopropanation (similar to pathway **c**, Scheme 1) of pyrrole-2,3-diones using stabilized sulfur ylides, yielding 2-azabicyclo[3.1.0]hexanes [18]. The resulting cyclopropanes could be ring-opened under acidic conditions (e.g., with sulfuric acid) to afford the corresponding pyridines.

As part of our ongoing studies on the dipolar reactivity of 1*H*-pyrrole-2,3-diones [18,21,22,23], we herein report their reaction with stabilized azomethine ylides derived from pyridine and alkyl bromides. This transformation affords unusually stable zwitterionic intermediates, which can be converted to cyclopropane-fused pyrroles via controlled thermolysis.

## 2. Results and Discussion

**Scheme 2:**
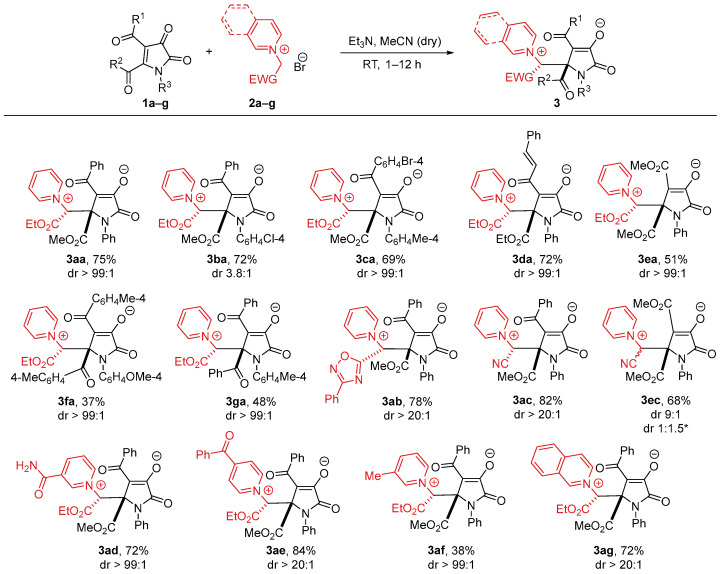
Synthesis of betaines **3**. * In a DMSO-*d*_6_ solution of **3ec**, the dr value changed from 9:1 to 1:1.5 within 30 min, resulting in the minor isomer becoming the major one.

Our study commenced with a model reaction of 1*H*-pyrrole-2,3-dione **1a** with quaternary salt **2a** in the presence of NEt_3_ in dry acetonitrile at room temperature (Scheme 2). The reaction proceeded smoothly under these mild conditions to afford the poorly soluble zwitterionic adduct **3aa**, which precipitated from the reaction mixture in a high yield. While the choice of solvent had a negligible effect on the product yield, elevating the reaction temperature to 60 °C led to a decrease in yield and the formation of unidentifiable side products, as monitored by HPLC analysis.

The structures of compounds **3aa** and **3da** were unambiguously confirmed by single-crystal X-ray diffraction analysis (**3aa**: CCDC 2500563; **3da**: CCDC 2500564). The unusual stability of this zwitterionic product arises from extensive negative charge delocalization across the 1,3-dicarbonyl system, as evidenced by the X-ray diffraction data. The bond lengths within this moiety (see Table S2 in the SI for selected bond lengths) indicate strong delocalization, with C–O and C–C distances ranging from 1.23 to 1.25 Å and 1.40 to 1.43 Å, respectively.

To explore the generality of this transformation, we investigated a range of pyrrole-2,3-diones, **1a**–**g**, bearing different carbonyl groups. The reaction proved to be tolerant of various substituents, including aroyl, alkoxycarbonyl and cinnamoyl groups in different combinations, providing the desired betaines **3aa**–**ga**. Oxadiazole and nitrile substituents were successfully employed as electron-withdrawing groups (EWGs) in the structures of the starting salts **2**, which allowed for the synthesis of the corresponding products **3ab**, **3ac** and **3ec**. Compounds **3ad**–**3ag** were prepared from salts **2d**–**g**, derived from substituted pyridines and isoquinoline, showcasing the possibility of utilizing other functionalized azaheterocycles instead of pyridine. The yields of the products **3** were generally good with moderate to excellent diastereoselectivity (the dr values were determined by an ^1^H NMR analysis of the isolated products). The most characteristic NMR distinction between the diastereomers was the chemical shift of the methine proton at the chiral carbon, observed as a singlet at 7.0–7.5 ppm for the major diastereomer and at 6.5–7.0 ppm for the minor one.

Betaines **3** are capable of epimerization in solution. Indeed, we observed rapid epimerization for compound **3ec** in DMSO-*d*_6_ during the recording of the NMR spectra (see the SI). Given the possibility of epimerization, the lower dr value for compound **3ba** can be explained by the different solubility of the diastereomers.

The electronic nature of the substituents on the pyrrole-2,3-dione core was found to be crucial for the success of the transformation. The reaction did not proceed for pyrrole-2,3-diones lacking strong electron-withdrawing groups at both the 4- and 5-positions or for those bearing only a single EWG at the 4-position. In these cases, HPLC analysis did not reveal consumption of the starting materials or the formation of any identifiable product.

Next, we investigated the thermal reactivity of the synthesized betaines **3**. Based on literature precedents (Scheme 1), we anticipated the two most probable formal pathways: a (2 + 1) annulation involving the C=C bond of the pyrrole-2,3-dione to form cyclopropanes or a (4 + 1) annulation of the enone moiety to yield dihydrofurans.

Initial studies focused on the thermolysis of betaine **3aa** as a model reaction. Thermal analysis of **3aa** (see Figure S3 in the SI) revealed an endothermic melting at 170–190 °C, accompanied by mass loss consistent with pyridine elimination, followed by an exothermic effect. Attempts to thermolyze **3aa** in solvent-free conditions at 180 °C resulted in numerous by-products, prompting us to conduct the reaction at a lower temperature. Refluxing a suspension of **3aa** in 1,4-dioxane until complete dissolution afforded cyclopropane **4aa** as the major product, albeit with a modest 24% HPLC yield (Table 1, entry 1). Given the promising initial result, we proceeded to optimize the reaction conditions. A screen of various solvents showed that refluxing in chlorobenzene gave the best outcome (entries 1–13). Finally, increasing the concentration in chlorobenzene from 0.05 M to 0.2 M did not diminish the yield (entry 14, optimal conditions for the synthesis of cyclopropanes **4**).

It is important to note that attempts to synthesize cyclopropane **4aa** via a one-pot procedure from pyrrole-2,3-dione **1a** and salt **2a** under these thermal conditions (PhCl, reflux) were unsuccessful, yielding only complex mixtures (see the complete optimization Table S1 in the SI). This finding underscores the necessity of isolating the betaine intermediate **3aa** for the efficient formation of the cyclopropane core.

Furthermore, we discovered that the reaction temperature is critical for reaction outcome (Table 1, entries 12, 13). When the thermolysis of **3aa** was performed in *o*-dichlorobenzene (b.p. 181 °C), the initially formed cyclopropane **4aa** underwent ring-opening to furnish pyridine **5aa**. This provides a direct, albeit lower-yielding (37% vs. 81% via H_2_SO_4_-mediated ring-opening for **5aa**) [18], route to valuable polysubstituted pyridine derivatives **5** from betaines **3** without the need to isolate cyclopropanes **4**. However, further optimization of the pyridine synthesis proved unsuccessful, as the high temperatures required for the cyclopropane ring-opening promoted competitive side reactions (see entry 19 of the Table S1 in the SI; cyclopropane **4aa** itself was unstable at 190 °C, giving pyridine-2,3-dione **5aa** and numerous unidentified products).

**Scheme 3:**
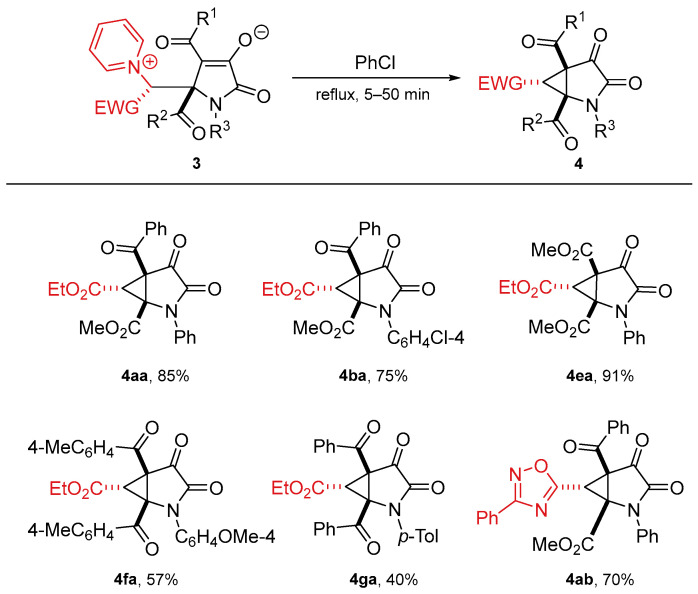
Synthesis of cyclopropanes **4**.

The optimized conditions (Table 1, entry 14: PhCl, 0.2M, reflux) were applied to the synthesis of a series of cyclopropane derivatives **4** (Scheme 3). Cyclopropanes **4** were formed as a single diastereomer, consistent with the synthesis of the same cyclopropanes using sulfur ylides [18]. Attempts to isolate cyclopropanes **4da** and **4ac** (functionalized with cinnamoyl and nitrile groups) were unsuccessful due to side reactions that led to complex mixtures.

**Scheme 4:**
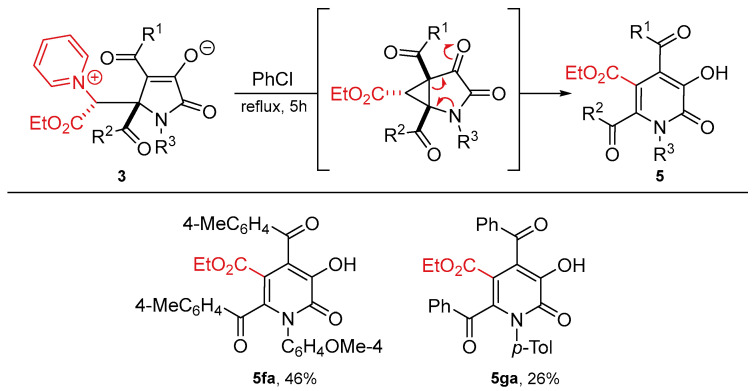
Synthesis of pyridine-2,3-diones **5**.

Cyclopropanes with two aroyl substituents were found to be more prone to ring-opening under thermolysis conditions. Consequently, the brief heating of the diaroyl-substituted betaines afforded cyclopropanes **4fa** and **4ga** in moderate yields (Scheme 3). In contrast, prolonged reflux in chlorobenzene resulted in ring-opening, yielding pyridines **5fa** and **5ga** as the major products (Scheme 4).

In conclusion, we have investigated the reaction of pyrrole-2,3-diones with stabilized azomethine ylides, generated in situ from pyridinium (or isoquinolinium) salts. This process affords unusually stable zwitterionic adducts, which are typically observed only as intermediates in analogous reactions. We studied the thermal behavior of these betaines, which enabled the development of a method for their conversion to cyclopropane-fused pyrroles **4**, and in some cases to pyridine-2,3-diones **5**, via thermal pyridine-elimination/cyclization in chlorobenzene. Although the synthesis of cyclopropane-fused pyrroles **4** from pyridinium ylides requires the preliminary isolation of betaines, the method can be useful as an alternative to the use of sulfonium ylides [18], particularly when pyridinium salts are more readily available than sulfonium salts.

## 3. Materials and Methods

^1^H, ^13^C NMR spectra were recorded on a Bruker Avance III HD spectrometer (400, 101 MHz, respectively; Bruker Corporation, Billerica, MA, USA) at 40 °C (313 K) in CDCl_3_ and DMSO-*d*_6_ using the residual solvent peak (CDCl_3_: δH = 7.26 ppm, δC = 77.16 ppm; DMSO-*d*_6_: δH = 2.50 ppm, δC = 39.52 ppm) as internal standards. Splitting patterns of apparent multiplets were designated as s (singlet), d (doublet), t (triplet), q (quartet), m (multiplet) and br (broadened). FT-IR spectra were recorded on a Perkin–Elmer Spectrum Two spectrometer (PerkinElmer, Waltham, MA, USA) from mulls in mineral oil. Melting points were measured with a Mettler Toledo MP70 Melting Point apparatus (Mettler-Toledo International Inc., Greifensee, Zurich, Switzerland). Elemental analysis was carried out on a Vario MICRO Cube analyzer (Elementar Analysensysteme GmbH, Langenselbold, Germany). Thin-layer chromatography (TLC) was performed on silica gel 60 F254 plates (Merck KGaA, Darmstadt, Germany); spots were visualized with UV light (254 nm/365 nm) or iodine vapors. Flash-column chromatography was performed on silica gel (Acros Organics, 35–70 μm, Geel, Belgium). HPLC analysis was performed on Hitachi Chromaster equipped with PDA detector Hitachi Chromaster 5430 (NUCLEODUR C18 Gravity column 3 μm, 4 × 150 mm) (Hitachi High-Tech Corporation, Tokyo, Japan; Macherey-Nagel GmbH & Co. KG, Düren, Germany). Thermal analysis was performed on NETZSCH Jupiter STA 449 F1 (NETZSCH Gerätebau GmbH, Selb, Bavaria, Germany). X-ray diffraction analysis was performed on an Xcalibur Ruby diffractometer (Malvern Panalytical Ltd., Malvern, Worcestershire, UK) using a Mo X-ray source (MoKα 0.71073 Å), by scanning at 295(2) K. All solvents and reagents were purchased from commercial vendors and were used as received. Solvent drying was performed by standard methods. MeCN and PhCl were stored over 4 Å molecular sieves.

Starting materials were prepared according to known procedures: 1*H*-pyrrole-2,3-diones—**1a**–**g** [18]; pyridinium/isoquinolinium salts—**2a**,**c**,**f**,**g** [24], **2d** [25].

### 3.1. Synthesis of the Pyridinium Salts

Salt **2b**: Pyridine (175 μL, 1.1 equiv.) is added to the solution of 5-(bromomethyl)-3-phenyl-1,2,4-oxadiazole [26] (478 mg, 2 mmol, 1 equiv.) in acetone (4 mL), and the solution is stirred at RT for 1 day. The resulting precipitate is filtered, washed with acetone and dried at 60 °C. Off-white solid, 517 mg (81%), m.p. 172–174 °C. ^1^H NMR (400 MHz, DMSO-*d*_6_): δ 8.66–8.49 (m, 2H), 8.04 (tt, J = 7.8, 1.4 Hz, 1H), 7.56 (dd, J = 7.9, 6.6 Hz, 2H), 7.32–7.04 (m, 2H), 6.93–6.64 (m, 3H), 5.81 (s, 2H). ^13^C NMR (101 MHz, DMSO-*d*_6_): δ 173.5, 167.6, 147.4, 146.3 (2C), 131.8, 129.2 (2C), 128.2 (2C), 126.9 (2C), 125.2, 55.1. Anal. Calcd for C_14_H_12_BrN_3_O: C 52.85; H 3.80; N 13.21. Found: C 52.97; H 3.69; N 13.41.

Salt **2e**: 4-benzoylpyridine (915 mg, 1 equiv.) and ethyl bromoacetate (1.1 mL, 2 equiv.) are added to the screw-capped vial. The vial is placed in a preheated-to-80 °C heating block and stirred at this temperature overnight. The formed orange-colored viscous oil is triturated with acetone to form an off-white powder, which is filtered and washed with acetone. Off-white powder, 1.17 g (68%), m.p. 139–141 °C (dec.). ^1^H NMR (400 MHz, DMSO-*d*_6_): δ 9.32 (d, *J* = 6.8 Hz, 2H), 8.47 (d, *J* = 6.8 Hz, 2H), 7.89–7.77 (m, 3H), 7.71–7.60 (m, 2H), 5.84 (s, 2H), 4.28 (q, *J* = 7.1 Hz, 2H), 1.29 (t, *J* = 7.1 Hz, 3H). ^13^C NMR (101 MHz, DMSO-*d*_6_): δ 191.7, 166.0, 152.3, 147.2 (2C), 134.7, 133.9, 130.1 (2C), 129.0 (2C), 126.6 (2C), 62.3, 60.4, 13.8. Anal. Calcd for C_16_H_16_BrNO_3_: C 54.87; H 4.61; N 4.00. Found: C 55.25; H 4.67; N 4.15.

### 3.2. Synthesis of Betaines 3

General Procedure A: To a round-bottom flask containing a magnetic stir bar and pyrrole-2,3-dione **1** (1 equiv.), dry acetonitrile (10 mL per 1 mmol of **1**) is added, followed by salt **2** (1.1 equiv.) and triethylamine (1.1 equiv.). The resulting mixture is stirred at room temperature for 1–2 h. The formed precipitate is filtered and washed with acetonitrile.

(S*)-4-Benzoyl-5-((R*)-2-ethoxy-2-oxo-1-(pyridin-1-ium-1-yl)ethyl)-5-(methoxycarbonyl)-2-oxo-1-phenyl-2,5-dihydro-1*H*-pyrrol-3-olate (**3aa**). Synthesized according to General Procedure A from pyrrole-2,3-dione **1a** (1 mmol scale) and salt **2a**. A pale-yellow/off-white powder, 376 mg (75%), m.p. 166–168 °C (dec.). ^1^H NMR (400 MHz, DMSO-*d*_6_): δ 8.95–8.84 (m, 2H), 8.64–8.54 (m, 1H), 8.05 (t, *J* = 7.1 Hz, 2H), 7.50 (dd, *J* = 8.7, 6.7 Hz, 2H), 7.43–7.33 (m, 3H), 7.33–7.24 (m, 1H), 7.24–7.11 (m, 5H), 3.82 (s, 3H), 3.65 (dq, *J* = 10.7, 7.1 Hz, 1H), 3.05 (dq, *J* = 10.7, 7.1 Hz, 1H), 0.97 (t, *J* = 7.1 Hz, 3H). ^13^C NMR (101 MHz, DMSO-*d*_6_): δ 184.6, 169.4, 169.1, 167.4, 164.9, 147.0, 146.5 (2C), 140.6, 135.7, 129.1, 128.7 (2C), 127.9 (2C), 127.5, 127.3 (2C), 126.5 (4C), 106.1, 71.6, 70.8, 62.1, 52.8, 13.1. IR (mineral oil), cm^−1^: 1741, 1729, 1713, 1654, 1632, 1582. Anal. Calcd for C_28_H_24_N_2_O_7_: C 67.19; H 4.83; N 5.60. Found: C 67.46; H 4.78; N 5.81.

(S*)-4-Benzoyl-1-(4-chlorophenyl)-5-((R*)-2-ethoxy-2-oxo-1-(pyridin-1-ium-1-yl)ethyl)-5-(methoxycarbonyl)-2-oxo-2,5-dihydro-1*H*-pyrrol-3-olate (**3ba**). Synthesized according to General Procedure A from pyrrole-2,3-dione **1b** (0.5 mmol scale) and salt **2a**. An off-white powder, 192 mg (72%), m.p. 167–175 °C (dec.). ^1^H NMR (400 MHz, DMSO-*d*_6_), mixture of diastereomers, d.r. ∼3.8:1 (A:B): δ 9.04 (d, *J* = 5.7 Hz, 0.42H, B), 8.89 (d, *J* = 5.6 Hz, 1.58H, A), 8.59 (t, *J* = 7.8 Hz, 1H), 8.08–8.00 (m, 2H), 7.82 (d, *J* = 7.0 Hz, 0.42H, B), 7.57 (d, *J* = 8.8 Hz, 1.58H, A), 7.46–7.14 (m, 7.37H, A + B), 6.79 (d, *J* = 8.8 Hz, 0.42H, B), 6.52 (s, 0.21H, B), 4.04–3.88 (m, 0.42H, B), 3.81 (s, 2.37H, A), 3.80 (s, 0.63H, B), 3.72 (dq, *J* = 10.7, 7.1 Hz, 0.79H, A), 3.19 (dq, *J* = 10.8, 7.2 Hz, 0.79H, A), 1.10 (t, *J* = 7.1 Hz, 0.63H, B), 1.01 (t, *J* = 7.1 Hz, 2.37H, A). ^13^C NMR (101 MHz, DMSO-*d*_6_), A (major): δ 184.6, 169.4, 169.1, 167.0, 164.9, 147.0, 146.6 (2C), 140.5, 134.7, 132.1, 129.2, 129.1 (2C), 128.7 (2C), 127.9 (2C), 126.5 (4C), 106.0, 71.5, 70.7, 62.2, 53.0, 13.0. IR (mineral oil), cm^−1^:1758, 1737, 1714, 1650, 1627, 1580, 1529. Anal. Calcd for C_28_H_23_ClN_2_O_7_: C 62.87; H 4.33; N 5.24. Found: C 63.10; H 4.27; N 5.35.

(S*)-4-(4-Bromobenzoyl)-5-((R*)-2-ethoxy-2-oxo-1-(pyridin-1-ium-1-yl)ethyl)-5-(methoxycarbonyl)-2-oxo-1-(*p*-tolyl)-2,5-dihydro-1*H*-pyrrol-3-olate (**3ca**). Synthesized according to General Procedure A from pyrrole-2,3-dione **1c** (1 mmol scale) and salt **2a**. A pale-yellow powder, 408 mg (69%), m.p. 188–189 °C (dec.). ^1^H NMR (400 MHz, DMSO-*d*_6_): δ 8.87 (d, *J* = 5.7 Hz, 2H), 8.59 (tt, *J* = 7.8, 1.3 Hz, 1H), 8.04 (t, *J* = 7.1 Hz, 2H), 7.43–7.34 (m, 2H), 7.32–7.25 (m, 2H), 7.27–7.18 (m, 2H), 7.18–7.06 (m, 3H), 3.79 (s, 3H), 3.68 (dq, *J* = 10.7, 7.1 Hz, 1H), 3.11 (dq, *J* = 10.7, 7.2 Hz, 1H), 2.35 (s, 3H), 0.98 (t, *J* = 7.1 Hz, 3H). ^13^C NMR (101 MHz, DMSO-*d*_6_): δ 182.9, 169.4, 168.9, 167.9, 164.8, 147.0, 146.4 (2C), 139.7, 137.2, 133.0, 130.0 (2C), 129.6 (2C), 129.2 (2C), 127.2 (2C), 126.5 (2C), 122.5, 105.9, 71.4, 70.7, 62.2, 52.8, 20.5, 13.0. IR (mineral oil), cm^−1^: 1743, 1728, 1712, 1648, 1632, 1571, 1511. Anal. Calcd for C_29_H_25_BrN_2_O_7_: C 58.70; H 4.25; N 4.72. Found: C 59.08; H 4.27; N 5.01.

(S*)-4-Cinnamoyl-5-((R*)-2-ethoxy-2-oxo-1-(pyridin-1-ium-1-yl)ethyl)-5-(ethoxycarbonyl)-2-oxo-1-phenyl-2,5-dihydro-1*H*-pyrrol-3-olate (**3da**). Synthesized according to General Procedure A from pyrrole-2,3-dione **1d** (1 mmol scale) and salt **2a**. A light-yellow powder, 390 mg (72%), m.p. 186–188 °C (dec.). ^1^H NMR (400 MHz, DMSO-*d*_6_): δ 8.86 (d, *J* = 5.3 Hz, 2H), 8.57 (t, *J* = 7.8 Hz, 1H), 8.05 (t, *J* = 7.3 Hz, 2H), 8.00 (d, *J* = 16.0 Hz, 1H), 7.53–7.28 (m, 11H), 7.18 (d, *J* = 15.9 Hz, 1H), 4.33–4.20 (m, 2H), 3.64 (dq, *J* = 10.7, 7.1 Hz, 1H), 3.07 (dq, *J* = 10.8, 7.1 Hz, 1H), 1.20 (t, *J* = 7.1 Hz, 3H), 0.97 (t, *J* = 7.1 Hz, 3H). ^13^C NMR (101 MHz, DMSO-*d*_6_): δ 178.4, 169.3, 168.8, 168.3, 164.9, 147.0, 146.3 (2C), 135.8, 135.8, 135.7, 128.8, 128.7 (2C), 128.6 (2C), 127.5, 127.3 (2C), 127.2 (2C), 127.1, 126.4 (2C), 108.1, 71.5, 70.7, 62.1, 61.9, 13.7, 13.0. IR (mineral oil), cm^−1^: 1751, 1731, 1715, 1634. Anal. Calcd for C_31_H_28_N_2_O_7_: C 68.88; H 5.22; N 5.18. Found: C 69.19; H 5.34; N 5.10.

(S*)-5-((R*)-2-Ethoxy-2-oxo-1-(pyridin-1-ium-1-yl)ethyl)-4,5-bis(methoxycarbonyl)-2-oxo-1-phenyl-2,5-dihydro-1*H*-pyrrol-3-olate (**3ea**). Synthesized according to General Procedure A from pyrrole-2,3-dione **1e** (1 mmol scale) and salt **2a**. An off-white powder, 265 mg (58%), m.p. 152–154 °C (dec.). ^1^H NMR (400 MHz, DMSO-*d*_6_): δ 8.88–8.82 (m, 2H), 8.65 (tt, *J* = 7.8, 1.3 Hz, 1H), 8.13 (t, *J* = 7.4 Hz, 2H), 7.49–7.43 (m, 2H), 7.40–7.32 (m, 3H), 7.11 (s, 1H), 3.79 (s, 3H), 3.60 (dq, *J* = 10.8, 7.1 Hz, 1H), 3.31 (s, 3H), 3.04 (dq, *J* = 10.8, 7.1 Hz, 1H), 0.95 (t, *J* = 7.1 Hz, 3H). ^13^C NMR (101 MHz, DMSO-*d*_6_): δ 169.2, 169.1, 166.3, 164.8, 164.2, 147.2, 146.2 (2C), 135.8, 128.6 (2C), 127.3, 127.2 (2C), 126.7 (2C), 92.4, 71.0, 70.7, 62.1, 52.9, 49.0, 13.0. IR (mineral oil), cm^−1^: 1743, 1712, 1655, 1629, 1607, 1589. Anal. Calcd for C_23_H_22_N_2_O_8_: C 60.79; H 4.88; N 6.16. Found: C 60.91; H 4.92; N 6.10.

(S*)-5-((R*)-2-Ethoxy-2-oxo-1-(pyridin-1-ium-1-yl)ethyl)-1-(4-methoxyphenyl)-4,5-bis(4-methylbenzoyl)-2-oxo-2,5-dihydro-1*H*-pyrrol-3-olate (**3fa**). Synthesized according to General Procedure A from pyrrole-2,3-dione **1f** (1 mmol scale) and salt **2a**. A light-yellow powder, 227 mg (37%), m.p. 146–148 °C (dec.). ^1^H NMR (400 MHz, DMSO-*d*_6_): δ 8.94 (d, *J* = 5.3 Hz, 2H), 8.55 (tt, *J* = 7.8, 1.3 Hz, 1H), 8.05–7.97 (m, 2H), 7.61 (d, *J* = 8.3 Hz, 2H), 7.25–7.13 (m, 5H), 7.05–6.92 (m, 6H), 3.83–3.76 (m, 1H), 3.75 (s, 3H), 3.38 (dq, *J* = 10.8, 7.1 Hz, 1H), 2.29 (s, 3H), 2.24 (s, 3H), 1.03 (t, *J* = 7.1 Hz, 3H). ^13^C NMR (101 MHz, DMSO-*d*_6_): δ 192.2, 184.2, 169.0, 168.0, 165.1, 157.9, 146.7, 146.5 (2C), 146.2, 142.2, 138.7, 137.7, 133.9, 128.7 (2C), 127.9 (2C), 127.9 (2C), 127.7 (2C), 127.1 (2C), 126.3 (2C), 113.8 (2C), 105.2, 75.9, 71.2, 62.3, 55.3, 20.8 (2C), 13.1. IR (mineral oil), cm^−1^: 1752, 1737, 1710, 1680, 1647, 1605, 1571. Anal. Calcd for C_36_H_32_N_2_O_7_: C 71.51; H 5.33; N 4.63. Found: C 71.66; H 5.54; N 4.47.

(S*)-4,5-Dibenzoyl-5-((R*)-2-ethoxy-2-oxo-1-(pyridin-1-ium-1-yl)ethyl)-2-oxo-1-(*p*-tolyl)-2,5-dihydro-1*H*-pyrrol-3-olate (**3ga**). Synthesized according to General Procedure A from pyrrole-2,3-dione **1g** (0.94 mmol scale) and salt **2a**. An off-white powder, 247 mg (48%), m.p. 151–153 °C (dec.). ^1^H NMR (400 MHz, DMSO-*d*_6_): δ 8.97 (d, *J* = 5.2 Hz, 2H), 8.57 (tt, *J* = 7.8, 1.3 Hz, 1H), 8.07–8.02 (m, 2H), 7.69–7.62 (m, 2H), 7.52–7.46 (m, 1H), 7.38–7.32 (m, 2H), 7.27–7.19 (m, 6H), 7.17–7.09 (m, 2H), 7.04–6.98 (m, 2H), 3.77 (dq, *J* = 10.8, 7.1 Hz, 1H), 3.35 (dq, *J* = 10.8, 7.1 Hz, 1H), 2.30 (s, 3H), 1.02 (t, *J* = 7.1 Hz, 3H). ^13^C NMR (101 MHz, DMSO-*d*_6_): δ 192.8, 184.4, 168.8, 168.1, 165.0, 146.8, 146.5 (2C), 140.6, 136.7, 136.6, 132.7, 131.9, 129.1 (2C), 129.0, 128.2 (2C), 127.7 (2C), 127.6 (2C), 126.5 (2C), 126.4 (2C), 126.0 (2C), 104.9, 75.6, 71.1, 62.3, 20.4, 13.1. IR (mineral oil), cm^−1^: 1747, 1719, 1681, 1647, 1597, 1581. Anal. Calcd for C_34_H_28_N_2_O_6_: C 72.85; H 5.03; N 5.00. Found: C 73.12; H 5.09; N 4.96.

(S*)-4-Benzoyl-5-(methoxycarbonyl)-2-oxo-1-phenyl-5-((R*)-(3-phenyl-1,2,4-oxadiazol-5-yl)(pyridin-1-ium-1-yl)methyl)-2,5-dihydro-1*H*-pyrrol-3-olate (**3ab**). Synthesized according to General Procedure A from pyrrole-2,3-dione **1a** (1 mmol scale) and salt **2b**. An off-white powder, 444 mg (78%), m.p. 173–175 °C (dec.). ^1^H NMR (400 MHz, DMSO-*d*_6_), mixture of diastereomers, d.r. ∼33:1 (A:B): δ 9.26 (d, *J* = 6.5 Hz, 0.06H, B), 9.17 (d, *J* = 5.8 Hz, 1.94H, A), 8.65 (t, *J* = 7.8 Hz, 0.97H, A), 8.61 (t, *J* = 7.7 Hz, 0.03H, B), 8.12 (t, *J* = 7.0 Hz, 1.94H, A), 8.02 (t, *J* = 7.2 Hz, 0.06H, B), 7.97 (s, 1H), 7.74 (d, *J* = 6.8 Hz, 2H), 7.64–7.50 (m, 3H), 7.39–7.30 (m, 3H), 7.28–7.18 (m, 4H), 7.13–7.02 (m, 2.94H), 6.88 (d, *J* = 7.8 Hz, 0.06H, B), 3.86 (s, 2.91H, A), 3.83 (s, 0.09H, B). ^13^C NMR (101 MHz, DMSO-*d*_6_), A (major): δ 184.8, 172.2, 170.2, 168.7, 167.4, 167.0, 147.7, 146.4 (2C), 140.1, 135.3, 131.6, 129.4, 129.0 (2C), 128.7 (2C), 128.1 (2C), 127.1 (2C), 127.0 (2C), 126.8, 126.7 (2C), 125.5 (2C), 125.1, 105.5, 72.2, 65.8, 53.1.IR (mineral oil), cm^−1^: 1743, 1732, 1651, 1629, 1582. Anal. Calcd for C_33_H_24_N_4_O_6_: C 69.22; H 4.23; N 9.79. Found: C 69.51; H 4.45; N 9.71.

(S*)-4-Benzoyl-5-((R*)-cyano(pyridin-1-ium-1-yl)methyl)-5-(methoxycarbonyl)-2-oxo-1-phenyl-2,5-dihydro-1*H*-pyrrol-3-olate (**3ac**). Synthesized according to General Procedure A from pyrrole-2,3-dione **1a** (1 mmol scale) and salt **2c**. A pale-beige powder, 370 mg (82%), m.p. 180–182 °C (dec.). ^1^H NMR (400 MHz, DMSO-*d*_6_), mixture of diastereomers, d.r. ∼50:1, A (major): δ 8.91 (d, *J* = 5.5 Hz, 2H), 8.67 (tt, *J* = 7.9, 1.3 Hz, 1H), 8.12 (dd, *J* = 7.8, 6.7 Hz, 2H), 7.61–7.54 (m, 4H), 7.51–7.45 (m, 1H), 7.42–7.28 (m, 5H), 7.27 (s, 1H), 3.77 (s, 3H). ^13^C NMR (101 MHz, DMSO-*d*_6_), A (major): δ 184.9, 170.0, 168.3, 166.9, 148.3, 145.1 (2C), 139.5, 134.8, 129.8, 129.5 (2C), 128.5 (2C), 128.3, 127.7 (2C), 127.2 (2C), 126.8 (2C), 113.9, 103.6, 71.2, 63.2, 53.1. IR (mineral oil), cm^−1^:1734, 1714, 1653, 1629, 1595, 1581 (CN-peak was not observed even in KBr-tablet). Anal. Calcd for C_26_H_19_N_3_O_5_: C 68.87; H 4.22; N 9.27. Found: C 69.16; H 4.13; N 9.26.

(5-(Cyano(pyridin-1-ium-1-yl)methyl)-4,5-bis(methoxycarbonyl)-2-oxo-1-phenyl-2,5-dihydro-1*H*-pyrrol-3-olate (**3ec**). Synthesized according to General Procedure A from pyrrol-2,3-dione **1e** (0.4 mmol scale) and salt **2c**. A pale-beige powder, 110 mg (68%), m.p. 176–178 °C (dec.). ^1^H NMR (400 MHz, DMSO-*d*_6_), mixture of diastereomers, d.r. ∼9:1 (A:B): δ 9.08 (d, *J* = 5.5 Hz, 1.8H, A), 8.87 (d, *J* = 5.5 Hz, 0.2H, B), 8.73 (tt, *J* = 7.9, 1.3 Hz, 0.1H, B), 8.64 (tt, *J* = 7.8, 1.3 Hz, 0.9H, A), 8.20 (dd, *J* = 7.8, 6.7 Hz, 0.2H, B), 8.15–8.06 (m, 1.8H, A), 7.58–7.53 (m, 0.2H, B), 7.52–7.39 (m, 2.8H, A + B), 7.28–7.25 (m, 0.2H, B), 7.24–7.17 (m, 1.8H, A), 7.08 (s, 0.1H, B), 6.64 (s, 0.9H, A), 3.78 (s, 2.7H, A), 3.74 (s, 0.3H, B), 3.33 (s, 0.3H, B), 3.14 (s, 2.7H, A). 13C spectral signals are listed together for both diastereomers due to rapid epimerization in solution (see NMR ^1^H spectrum recorded 30 min after dissolution in the SI, d.r. ∼1:1.5 (A:B)). ^13^C NMR (101 MHz, DMSO-*d*_6_), A + B: δ 170.2, 170.1, 168.0, 167.9, 167.0, 166.5, 164.9, 164.6, 148.3, 147.6, 145.8, 145.3, 135.4, 134.8, 129.5, 129.4, 128.4, 128.1, 127.6, 127.6, 127.1, 126.9, 113.9, 113.1, 91.3, 89.9, 71.1, 70.9, 65.0, 64.0, 53.2, 53.0, 49.5, 49.1. IR (mineral oil), cm^−1^: 1748, 1723, 1694, 1679, 1634 (CN-peak was not observed even in KBr-tablet). Anal. Calcd for C_21_H_17_N_3_O_6_: C 61.92; H 4.21; N 10.31. Found: C 61.70; H 4.05; N 9.98.

(S*)-4-benzoyl-5-((R*)-1-(3-carbamoylpyridin-1-ium-1-yl)-2-ethoxy-2-oxoethyl)-5-(methoxycarbonyl)-2-oxo-1-phenyl-2,5-dihydro-1*H*-pyrrol-3-olate (**3ad**). Synthesized according to General Procedure A from pyrrole-2,3-dione **1a** (0.5 mmol scale) and salt **2d**. An off-white powder, 197 mg (72%), m.p. 148–150 °C (dec.). ^1^H NMR (400 MHz, DMSO-*d*_6_): δ 9.34 (s, 1H), 9.03 (d, *J* = 6.3 Hz, 1H), 8.97 (d, *J* = 8.2 Hz, 1H), 8.43 (s, 1H), 8.18 (dd, *J* = 8.1, 6.3 Hz, 1H), 8.04 (s, 1H), 7.54–7.47 (m, 2H), 7.42–7.34 (m, 3H), 7.31–7.26 (m, 1H), 7.24–7.15 (m, 5H), 3.79 (s, 3H), 3.71 (dq, *J* = 10.8, 7.1 Hz, 1H), 3.26–3.18 (m, 1H), 1.01 (t, *J* = 7.1 Hz, 3H). ^13^C NMR (101 MHz, DMSO-*d*_6_), A (major): δ 184.6, 169.4, 168.8, 167.5, 164.6, 162.1, 144.4, 140.6, 135.6, 131.7, 129.1, 128.8 (2C), 127.9 (2C), 127.5, 126.8 (2C), 126.6 (2C), 126.2, 105.6, 71.4, 71.3, 62.3, 52.9, 13.1. IR (mineral oil), cm^−1^: 3404, 3302, 1754, 1746, 1710, 1688, 1645, 1634, 1581. Anal. Calcd for C_29_H_25_N_3_O_8_: C 64.08; H 4.64; N 7.73. Found: C 64.29; H 4.75; N 7.63.

(S*)-4-benzoyl-5-((R*)-1-(4-benzoylpyridin-1-ium-1-yl)-2-ethoxy-2-oxoethyl)-5-(methoxycarbonyl)-2-oxo-1-phenyl-2,5-dihydro-1*H*-pyrrol-3-olate (**3ae**). Synthesized according to General Procedure A from pyrrole-2,3-dione **1a** (0.5 mmol scale) and salt **2e**. A beige powder, 253 mg (84%), m.p. 145–148 °C (dec.). ^1^H NMR (400 MHz, DMSO-*d*_6_), mixture of diastereomers, d.r. ∼30:1, A (major): δ 9.18 (d, *J* = 7.1 Hz, 2H), 8.21 (d, *J* = 6.9 Hz, 2H), 7.76 (tt, *J* = 7.2, 1.4 Hz, 1H), 7.61–7.47 (m, 6H), 7.42–7.31 (m, 6H), 7.28–7.20 (m, 3H), 3.80 (s, 3H), 3.74 (dq, *J* = 10.7, 7.1 Hz, 1H), 3.25–3.17 (m, 1H), 0.99 (t, *J* = 7.1 Hz, 3H). ^13^C NMR (101 MHz, DMSO-*d*_6_), A (major): δ 191.5, 184.6, 169.7, 169.0, 167.7, 164.6, 152.8, 147.6 (2C), 140.2, 135.7, 134.6, 133.8, 129.9 (2C), 129.4, 128.9 (2C), 128.8 (2C), 128.2 (2C), 127.4, 126.9 (2C), 126.7 (2C), 125.0 (2C), 106.0, 71.3, 71.1, 62.3, 52.9, 13.1. IR (mineral oil), cm^−1^: 1747, 1734, 1701, 1675, 1641, 1595, 1582, 1513. Anal. Calcd for C_35_H_28_N_2_O_8_: C 69.53; H 4.67; N 4.63. Found: C 69.20; H 4.79; N 4.61.

(S*)-4-benzoyl-5-((R*)-2-ethoxy-1-(3-methylpyridin-1-ium-1-yl)-2-oxoethyl)-5-(methoxycarbonyl)-2-oxo-1-phenyl-2,5-dihydro-1*H*-pyrrol-3-olate (**3af**). Synthesized according to General Procedure A from pyrrole-2,3-dione **1a** (0.5 mmol scale) and salt **2f**. An off-white powder, 98 mg (38%), m.p. 172–174 °C (dec.). ^1^H NMR (400 MHz, DMSO-*d*_6_): δ 8.78 (d, *J* = 6.3 Hz, 1H), 8.70 (s, 1H), 8.44–8.41 (m, 1H), 8.02–7.97 (m, 1H), 7.55–7.45 (m, 2H), 7.42–7.18 (m, 8H), 7.01 (s, 1H), 3.79 (s, 3H), 3.72 (dq, *J* = 10.7, 7.1 Hz, 1H), 3.26–3.18 (m, 1H), 2.24 (s, 3H), 0.99 (t, *J* = 7.1 Hz, 3H). ^13^C NMR (101 MHz, DMSO-*d*_6_), A (major): δ 184.5, 169.6, 169.1, 167.3, 164.8, 147.2, 145.9, 143.3, 140.4, 136.7, 135.7, 129.3, 128.8 (2C), 128.0 (2C), 127.4, 126.9 (2C), 126.6 (2C), 126.1, 105.9, 71.4, 70.8, 62.2, 52.7, 17.5, 13.1. IR (mineral oil), cm^−1^: 1740, 1732, 1725, 1631, 1584, 1534, 1518. Anal. Calcd for C_29_H_26_N_2_O_7_: C 67.70; H 5.09; N 5.44. Found: C 67.82; H 4.95; N 5.49.

(S*)-4-benzoyl-5-((R*)-2-ethoxy-1-(isoquinolin-2-ium-2-yl)-2-oxoethyl)-5-(methoxycarbonyl)-2-oxo-1-phenyl-2,5-dihydro-1*H*-pyrrol-3-olate (**3ag**). Synthesized according to General Procedure A from pyrrole-2,3-dione **1a** (0.5 mmol scale) and salt **2g**. A pale-yellow powder, 198 mg (72%), m.p. 175–177 °C (dec.). ^1^H NMR (400 MHz, DMSO-*d*_6_): δ 9.86 (s, 1H), 8.66 (d, *J* = 7.6 Hz, 1H), 8.46 (d, *J* = 7.0 Hz, 1H), 8.39 (d, *J* = 8.4 Hz, 1H), 8.30 (d, *J* = 8.3 Hz, 1H), 8.22 (ddd, *J* = 8.2, 6.8, 1.2 Hz, 1H), 7.96–7.91 (m, 1H), 7.56–7.49 (m, 2H), 7.44–7.35 (m, 3H), 7.24–7.11 (m, 2H), 7.10–6.97 (m, 4H), 3.80 (s, 3H), 3.78–3.67 (m, 1H), 0.99 (t, *J* = 7.1 Hz, 3H). The signal of one diastereotopic atom of the CH_2_ group is in the water region in ^1^H NMR. ^13^C NMR (101 MHz, DMSO-*d*_6_): δ 169.8, 169.1, 137.7, 135.8, 130.8, 129.1, 128.8, 127.8, 127.4, 127.0, 126.3, 125.8, 123.8, 71.5, 70.9, 62.2, 52.8, 13.1. Some of the carbon atom signals are not visible due to the very low solubility of the compound. IR (mineral oil), cm^−1^: 1744, 1732, 1714, 1646. Anal. Calcd for C_32_H_26_N_2_O_7_: C 69.81; H 4.76; N 5.09. Found: C 70.17; H 4.81; N 4.86.

### 3.3. Synthesis of Cyclopropanes 4

General Procedure B: To a V-vial containing a magnetic stir bar and betaine **3** (1 equiv.), dry chlorobenzene (5 mL per 1 mmol of **3**) is added. The vial is connected to a Vigreux column (serving as a condenser) and placed in a preheated-to-140 °C metal heating block. The resulting mixture is stirred at 140 °C until complete dissolution of the starting betaine **3** (5–50 min depending on the betaine used). The solution is cooled, and cyclopropanes **4** are isolated by flash-column chromatography (loaded directly on a column in PhCl) or crystallization.

6-Ethyl 1-methyl (1R*,5S*,6R*)-5-benzoyl-3,4-dioxo-2-phenyl-2-azabicyclo[3.1.0]hexane-1,6-dicarboxylate (**4aa**). Synthesized according to General Procedure B from betaine **3aa** (0.3 mmol scale). Isolated by FCC, Hexanes/EtOAc 2/1, R*_f_* 0.33. An off-white powder, 102 mg (81%), m.p. 158–160 °C (lit. [18] 159–161 °C). ^1^H NMR (400 MHz, CDCl_3_): δ 7.87–7.80 (m, 2H), 7.66 (t, *J* = 7.5 Hz, 1H), 7.60–7.49 (m, 4H), 7.48–7.41 (m, 2H), 7.30 (t, *J* = 7.4 Hz, 1H), 4.18 (q, *J* = 7.1 Hz, 2H), 4.15 (s, 1H), 3.58 (s, 3H), 1.16 (t, *J* = 7.1 Hz, 3H). ^13^C NMR (101 MHz, CDCl_3_): δ 186.6, 185.1, 165.4, 164.2, 156.7, 136.7, 135.0, 134.2, 129.7 (2C), 129.6 (2C), 129.3 (2C), 127.1, 120.1 (2C), 63.9, 54.8, 54.0, 46.8, 45.7, 13.9. IR (mineral oil), cm^−1^: 1785, 1756, 1742, 1724, 1671, 1595. The spectral data was consistent with the data reported in the literature [18].

6-Ethyl 1-methyl (1R*,5S*,6R*)-5-benzoyl-2-(4-chlorophenyl)-3,4-dioxo-2-azabicyclo[3.1.0]hexane-1,6-dicarboxylate (**4ba**). Synthesized according to General Procedure B from betaine **3ba** (0.37 mmol scale). Isolated by crystallization from EtOH. An off-white powder, 128 mg (75%), m.p. 166–169 °C (lit. [18] 167–169 °C). ^1^H NMR (400 MHz, CDCl_3_): δ 7.87–7.78 (m, 2H), 7.72–7.62 (m, 1H), 7.57–7.49 (m, 4H), 7.47–7.37 (m, 2H), 4.18 (q, *J* = 7.1 Hz, 2H), 4.13 (s, 1H), 3.60 (s, 3H), 1.17 (t, *J* = 7.1 Hz, 3H). ^13^C NMR (101 MHz, CDCl_3_): δ 186.3, 184.6, 165.3, 164.0, 156.6, 135.2, 135.1, 134.1, 132.7, 129.7 (2C), 129.7 (2C), 129.4 (2C), 121.3 (2C), 64.0, 54.6, 54.1, 46.8, 45.5, 14.0. IR (mineral oil), cm^−1^: 1782, 1758, 1749, 1722, 1674, 1596. The spectral data was consistent with the data reported in the literature [18].

6-Ethyl 1,5-dimethyl (1R*,5R*,6R*)-3,4-dioxo-2-phenyl-2-azabicyclo[3.1.0]hexane-1,5,6-tricarboxylate (**4ea**). Synthesized according to General Procedure B from betaine **3ea** (0.3 mmol scale). Isolated by FCC, Hexanes/EtOAc 3/2, R*_f_* 0.32. An off-white powder, 102 mg (91%), m.p. 129–131 °C. ^1^H NMR (400 MHz, CDCl_3_): δ 7.50–7.46 (m, 2H), 7.45–7.39 (m, 2H), 7.30–7.25 (m, 1H), 4.16 (s, 1H), 4.12 (qd, *J* = 7.2, 1.0 Hz, 2H), 3.84 (s, 3H), 3.69 (s, 3H), 1.13 (t, *J* = 7.1 Hz, 3H). ^13^C NMR (101 MHz, CDCl_3_): δ 183.6, 164.9, 163.7, 162.7, 156.6, 136.5, 129.6 (2C), 127.2, 120.2 (2C), 63.8, 55.1, 54.2 (2C), 45.4, 41.9, 13.9. IR (mineral oil), cm^−1^: 1777, 1747, 1724, 1597. The spectral data was consistent with the data reported in the literature [18].

Ethyl (1R*,5S*,6R*)-2-(4-methoxyphenyl)-1,5-bis(4-methylbenzoyl)-3,4-dioxo-2-azabicyclo[3.1.0]hexane-6-carboxylate (**4fa**). Synthesized according to General Procedure **B** from betaine **3fa** (0.3 mmol scale). Isolated by FCC, Hexanes/EtOAc 2/1, R*_f_* 0.5. A yellow powder, 90 mg (57%), m.p. 177–179 °C (lit. [18] 178–180 °C). ^1^H NMR (400 MHz, CDCl_3_): δ 7.84 (d, *J* = 8.0 Hz, 2H), 7.64 (d, *J* = 8.0 Hz, 2H), 7.32 (d, *J* = 8.0 Hz, 2H), 7.26 (d, *J* = 9.0 Hz, 2H), 7.12 (d, *J* = 8.0 Hz, 2H), 6.75 (d, *J* = 9.0 Hz, 2H), 4.25 (s, 1H), 4.25–4.18 (m, 2H), 3.71 (s, 3H), 2.45 (s, 3H), 2.33 (s, 3H), 1.19 (t, *J* = 7.1 Hz, 3H). ^13^C NMR (101 MHz, CDCl_3_) δ 190.4, 186.3, 185.3, 165.9, 158.2, 156.7, 146.5, 144.9, 132.9, 132.3, 130.6 (2C), 129.9 (2C), 129.3 (2C), 129.2, 128.9 (2C), 122.0 (2C), 114.6 (2C), 63.8, 60.0, 55.5, 49.8, 45.6, 22.0, 21.8, 14.0. IR (mineral oil), cm^−1^: 1778, 1769, 1737, 1678, 1602. The spectral data was consistent with the data reported in the literature [18].

Ethyl (1R*,5S*,6R*)-1,5-dibenzoyl-3,4-dioxo-2-(*p*-tolyl)-2-azabicyclo[3.1.0]hexane-6-carboxylate (**4ga**). Synthesized according to General Procedure B from betaine **3ga** (0.3 mmol scale). Isolated by FCC, Hexanes/EtOAc 3/1, R*_f_* 0.42. A pale-yellow/off-white powder, 57 mg (40%), m.p. 177–180 °C (dec.). ^1^H NMR (400 MHz, CDCl_3_): δ 7.97 (d, *J* = 7.2 Hz, 2H), 7.72–7.65 (m, 3H), 7.56–7.50 (m, 2H), 7.49–7.42 (m, 1H), 7.34–7.28 (m, 2H), 7.20 (d, *J* = 8.5 Hz, 2H), 7.02 (d, *J* = 8.4 Hz, 2H), 4.29 (s, 1H), 4.27–4.17 (m, 2H), 2.22 (s, 3H), 1.19 (t, *J* = 7.1 Hz, 3H). ^13^C NMR (101 MHz, CDCl_3_): δ 191.3, 186.9, 185.1, 165.8, 156.6, 137.0, 135.5, 135.2, 134.6, 133.7, 133.6, 130.4 (2C), 130.0 (2C), 129.2 (2C), 128.7 (2C), 128.6 (2C), 120.4 (2C), 63.9, 59.9, 50.0, 45.5, 21.0, 14.0. IR (mineral oil), cm^−1^: 1772, 1764, 1742, 1715, 1685, 1678, 1599, 1577. Anal. Calcd for C_29_H_23_NO_6_: C 72.34; H 4.81; N 2.91. Found: C 72.62; H 4.96; N 2.68.

Methyl (1R*,5S*,6R*)-5-benzoyl-3,4-dioxo-2-phenyl-6-(3-phenyl-1,2,4-oxadiazol-5-yl)-2-azabicyclo[3.1.0]hexane-1-carboxylate (**4ab**)**.** Synthesized according to General Procedure B from betaine **3ab** (0.3 mmol scale). Isolated by FCC, Hexanes/EtOAc 4/1, R*_f_* 0.31. An off-white powder, 104 mg (70%), m.p. 194–196 °C (lit. [18] 195–197 °C). ^1^H NMR (400 MHz, CDCl_3_): δ 7.92–7.79 (m, 4H), 7.68 (t, *J* = 7.4 Hz, 1H), 7.53 (t, *J* = 7.7 Hz, 2H), 7.50–7.33 (m, 7H), 7.25–7.18 (m, 1H), 4.76 (s, 1H), 3.64 (s, 3H). ^13^C NMR (101 MHz, CDCl_3_): δ 186.2, 184.2, 170.7, 168.9, 163.9, 156.7, 136.3, 135.3, 134.1, 132.0, 129.8 (2C), 129.7 (2C), 129.5 (2C), 129.1 (2C), 127.9 (2C), 127.4, 125.3, 120.0 (2C), 55.7, 54.2, 47.1, 38.1. IR (mineral oil), cm^−1^: 1774, 1755, 1739, 1682, 1597, 1579. The spectral data was consistent with the data reported in the literature [18].

### 3.4. Synthesis of Pyridine-2,3-Diones 5

General procedure C: To a V-vial containing a magnetic stir bar and betaine **3** (1 equiv.), dry chlorobenzene (5 mL per 1 mmol of **3**) is added. The vial is connected to a Vigreux column (serving as a condenser) and placed in a preheated-to-140 °C metal heating block. The resulting mixture is stirred at 140 °C until the complete consumption of cyclopropane **4** (TLC or HPLC control). The solution is cooled, PhCl evaporated under reduced pressure and the residue crystallized from EtOH/petroleum ether to obtain, after filtration and washing with cold EtOH, pyridine-2,3-diones **5**.

Ethyl 5-hydroxy-1-(4-methoxyphenyl)-2,4-bis(4-methylbenzoyl)-6-oxo-1,6-dihydropyridine-3-carboxylate (**5fa**). Synthesized according to General Procedure C from betaine **3fa** (0.3 mmol scale). Isolated by crystallization from EtOH. An off-white powder, 73 mg (46%), m.p. 217–219 °C (lit. [18] 218–220 °C). ^1^H NMR (400 MHz, CDCl_3_): δ 7.91 (d, *J* = 7.9 Hz, 2H), 7.54 (d, *J* = 8.0 Hz, 2H), 7.32 (d, *J* = 7.9 Hz, 2H), 7.16 (d, *J* = 7.9 Hz, 2H), 7.12–6.30 (br. m, 5H), 3.84 (q, *J* = 7.1 Hz, 2H), 3.74 (s, 3H), 2.45 (s, 3H), 2.38 (s, 3H), 0.71 (t, *J* = 7.1 Hz, 3H). ^13^C NMR (101 MHz, CDCl_3_) δ 191.7, 188.2, 163.3, 160.3, 159.3, 145.2, 144.9, 142.4, 142.3, 134.2, 134.2, 130.2 (2C), 129.6 (2C), 129.6 (2C), 129.5 (2C), 129.1 (2C), 128.1, 125.1, 114.5 (2C), 108.7, 62.1, 55.6, 21.9, 21.9, 13.0. IR (mineral oil), cm^−1^: 3259, 1727, 1686, 1677, 1651, 1606. The spectral data was consistent with the data reported in the literature [18].

Ethyl 2,4-dibenzoyl-5-hydroxy-6-oxo-1-(*p*-tolyl)-1,6-dihydropyridine-3-carboxylate (**5ga**). Synthesized according to General Procedure C from betaine **3ga** (0.14 mmol scale). Isolated by crystallization from EtOH. An off-white powder, 18 mg (26%), m.p. 208–209 °C, R*_f_* 0.45 (PhMe/EtOAc 5/1). ^1^H NMR (400 MHz, CDCl_3_): δ 8.06–7.99 (m, 2H), 7.68–7.58 (m, 3H), 7.58–7.47 (m, 3H), 7.37 (t, *J* = 7.8 Hz, 2H), 7.22–6.71 (br. m, 5H), 3.84 (q, *J* = 7.2 Hz, 2H), 2.27 (s, 3H), 0.70 (t, *J* = 7.2 Hz, 3H). ^13^C NMR (101 MHz, CDCl_3_): δ 192.0, 188.6, 163.3, 159.0, 142.6, 141.9, 140.1, 136.6, 136.5, 134.0, 133.9, 132.9, 129.9 (2C), 129.4 (2C), 129.0 (2C), 128.9 (2C), 128.8 (4C), 124.8, 108.9, 62.2, 21.3, 13.0. IR (mineral oil), cm^−1^: 3255, 1722, 1693, 1678, 1640, 1600. Anal. Calcd for C_29_H_23_NO_6_: C 72.34; H 4.81; N 2.91. Found: C 72.60; H 4.92; N 2.75.

### 3.5. Crystal Structure Determination

The unit cell parameters and the X-ray diffraction intensities were measured on an Xcalibur Ruby diffractometer. The empirical absorption correction was introduced by the multi-scan method using the SCALE3 ABSPACK algorithm [27]. Using the Olex2 [28], the structures were solved with the SHELXT [29] program and refined by the full-matrix least squares method in the anisotropic approximation for all non-hydrogen atoms with the SHELXL program [30]. Hydrogen atoms were positioned geometrically and refined using a riding model. Deposition Numbers CCDC 2500563 (for **3aa**) and CCDC 2500564 (for **3da**) contain the supplementary crystallographic data for this paper.

## Figures and Tables

**Table 1:** Optimization of the reaction conditions ^1^.

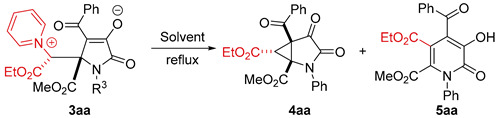				
Entry	Solvent (b.p., °C)	Time, h	Yield ^2^ of 4aa, %	Yield ^2^ of 5aa, %
1	1,4-Dioxane (101)	2	24	–
2	PhCl (132)	1	72	–
3	HFIP (58)	2	Traces	–
4	*i*-AmOH (131)	1	Traces	–
5	AcOH (118)	1	Traces	–
6	Dimethyl carbonate (90)	1	2	–
7	PhOMe (154)	1	55	–
8	BuOAc (126)	2	59	–
9	Methyl isobutyl ketone (117)	2	44	–
10	*N,N*-Dimethylacetamide (165)	1	Traces	–
11	*p*-Xylene (138)	1	69	–
12	o-Dichorobenzene (181)	1	28	20
13	o-Dichorobenzene (181)	2	8	37
14 ^3^	PhCl (132)	40 min	83	–

^1^ Reaction conditions: A mixture of **3aa** (0.05 mmol) and an anhydrous solvent (1000 µL; 0.05 M) was refluxed; ^2^ the yields were determined by HPLC using Ph_3_CH as an internal standard; ^3^ PhCl (250 µL; 0.2 M) was used; refluxing until complete dissolution of **3aa**.

## Data Availability

Data is contained within the article or the Supplementary Materials.

## Supplementary Materials

The following supporting information can be downloaded at https://www.mdpi.com/article/10.3390/molecules30234552/s1: General information; thermal analysis of betaine **3aa**; Table S1, optimization of the thermolysis of betaine **3aa**; general procedures for the synthesis of compounds **3**, **4** and **5**; crystal structure determination; copies of NMR spectra.
