# Supplementary material for: Synthesis of Stable Betaines Based on 1H-Pyrrole-2,3-diones and Pyridinium Ylides and Their Thermal Conversion to Cyclopropane-Fused Pyrroles
Source: Molecules. 2025 Nov 26;30(23):4552. doi: 10.3390/molecules30234552 (PMC12693529; doi:10.3390/molecules30234552)
Supplement: Supplementary file 1 [file molecules-30-04552-s001.zip › molecules-4001538-supplementary.pdf]

SUPPORTING INFORMATION

Synthesis of stable betaines based on 1*H*-pyrrole-2,3-diones and pyridinium ylides and their thermal conversion to cyclopropane-fused pyrroles

Maria M. Muranova, Andrey R. Galeev, Ivan G. Mokrushin, Andrey N. Maslivets and Maksim V. Dmitriev

Department of Chemistry, Perm State University, ul. Bukireva 15, Perm, 614990, Russian Federation

Table of Contents:

General information 2

Thermal analysis of betaine 3aa 3

Table S1. Optimization of the thermolysis of betaine 3aa 4

General procedures for the synthesis of compounds 3, 4 and 5 7

Crystal structure determination 16

Copies of NMR spectra 19

References 46

General information

^{1}H, ^{13}C NMR spectra were recorded on a Bruker Avance III HD spectrometer (400, 101 MHz, respectively) at 40 °C (313K) in $CDCl_3$ and DMSO-d_6, using the residual solvent peak ($CDCl_3$: δH = 7.26 ppm, δC = 77.16 ppm; DMSO-d_6: δH = 2.50 ppm; δC = 39.52 ppm) as internal standards. Splitting patterns of apparent multiplets were designated as s (singlet), d (doublet), t (triplet), q(quartet), m (multiplet) or br (broadened). FT-IR spectra were recorded on a Perkin–Elmer Spectrum Two spectrometer from mulls in mineral oil. Melting points were measured with Mettler Toledo MP70 Melting Point apparatus. Thin-layer chromatography (TLC) was performed on silica gel 60 F254 plates (Merck); spots were visualized with UV light (254 nm / 365 nm) or iodine vapors. Flash-column chromatography was performed on silica gel (Acros Organics, 35–70 μm). HPLC analysis was performed on Hitachi Chromaster equipped with PDA detector Hitachi Chromaster 5430 (NUCLEODUR C18 Gravity column 3 μm, 4 × 150 mm). Elemental analysis was carried out on a Vario MICRO Cube analyzer. Thermal analysis was performed on NETZSCH Jupiter STA 449 F1. X-ray diffraction analysis was performed on Xcalibur Ruby diffractometer using Mo X-ray source (MoKα 0.71073 Å), by scanning at 295(2) K. All solvents and reagents were purchased from commercial vendors and were used as received. Solvent drying was performed by standard methods. MeCN and PhCl were stored over 4Å molecular sieves.

Starting materials were prepared according to known procedures: 1*H*-pyrrole-2,3-diones (**Figure S1**) – **1a-g** [1]; pyridinium/isoquinolinium salts (**Figure S2**) – **2a,c,f,g** [2], **2d** [3].

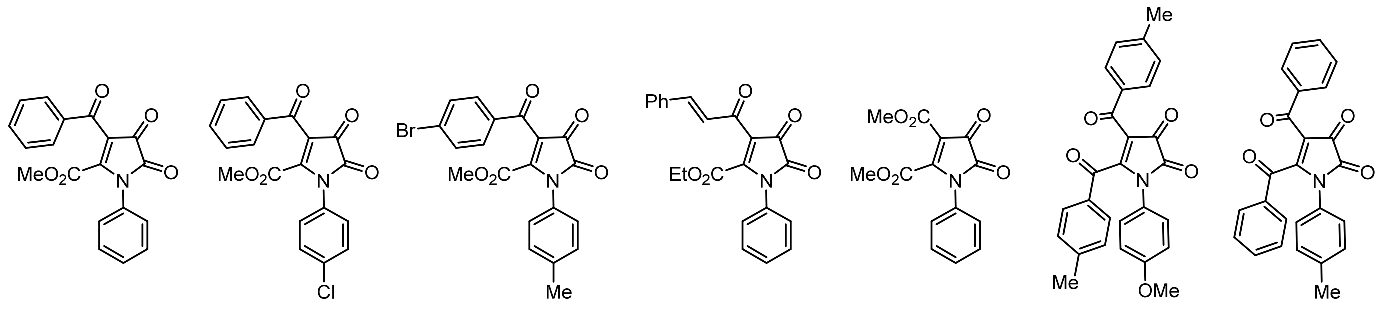

Figure S1. 1*H*-pyrrol-2,3-diones used in the present work

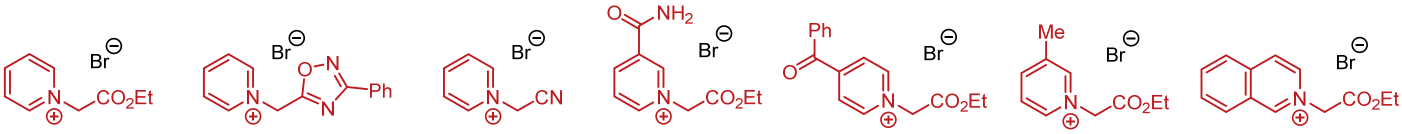

Figure S2. Pyridinium/isoquinolinium salts used in the present work

Synthesis of pyridinium salt **2b**: pyridine (175 μL, 1.1 equiv.) is added to the solution of 5-(bromomethyl)-3-phenyl-1,2,4-oxadiazole [4] (478 mg, 2 mmol, 1 equiv.) in acetone (4 mL), and the solution is stirred at RT for 1 day. The resulting precipitate is filtered, washed with acetone and dried at 60 °C. Off-white solid, 517 mg (81%), m.p. 172–174°C. **^{1}H NMR** (400 MHz, DMSO-d_6): δ 8.66 – 8.49 (m, 2H), 8.04 (tt, *J* = 7.8, 1.4 Hz, 1H), 7.56 (dd, *J* = 7.9, 6.6 Hz, 2H), 7.32 – 7.04 (m, 2H), 6.93 – 6.64 (m, 3H), 5.81 (s, 2H). **^{13}C NMR** (101 MHz, DMSO-d_6): δ 173.5, 167.6, 147.4, 146.3 (2C), 131.8, 129.2 (2C), 128.2 (2C), 126.9 (2C), 125.2, 55.1. Anal. Calcd for $C_{14}H_{12}BrN_3O$: C 52.85; H 3.80; N 13.21. Found: C 52.97; H 3.69; N 13.41.

Synthesis of pyridinium salt **2e**: 4-benzoylpyridine (915 mg, 1 equiv.) and ethyl bromoacetate (1.1 mL, 2 equiv.) are added to the screw-capped vial. The vial is placed in a preheated-to-80 °C heating block and stirred at this temperature overnight. The formed orange-colored viscous oil is triturated with acetone to form an off-white powder, which is filtered and washed with acetone. Off-white powder, 1.17 g (68%), m.p. 139–141°C (dec.). **^{1}H NMR** (400 MHz, DMSO-d_6): δ 9.32 (d, *J* = 6.8 Hz, 2H), 8.47 (d, *J* = 6.8 Hz, 2H), 7.89 – 7.77 (m, 3H), 7.71 – 7.60 (m, 2H), 5.84 (s, 2H), 4.28 (q, *J* = 7.1 Hz, 2H), 1.29 (t, *J* = 7.1 Hz, 3H). **^{13}C NMR** (101 MHz, DMSO-d_6): δ 191.7, 166.0, 152.3, 147.2 (2C), 134.7, 133.9, 130.1 (2C), 129.0 (2C), 126.6 (2C), 62.3, 60.4, 13.8. Anal. Calcd for $C_{16}H_{16}BrNO_3$: C 54.87; H 4.61; N 4.00. Found: C 55.25; H 4.67; N 4.15.

Thermal analysis of betaine 3aa

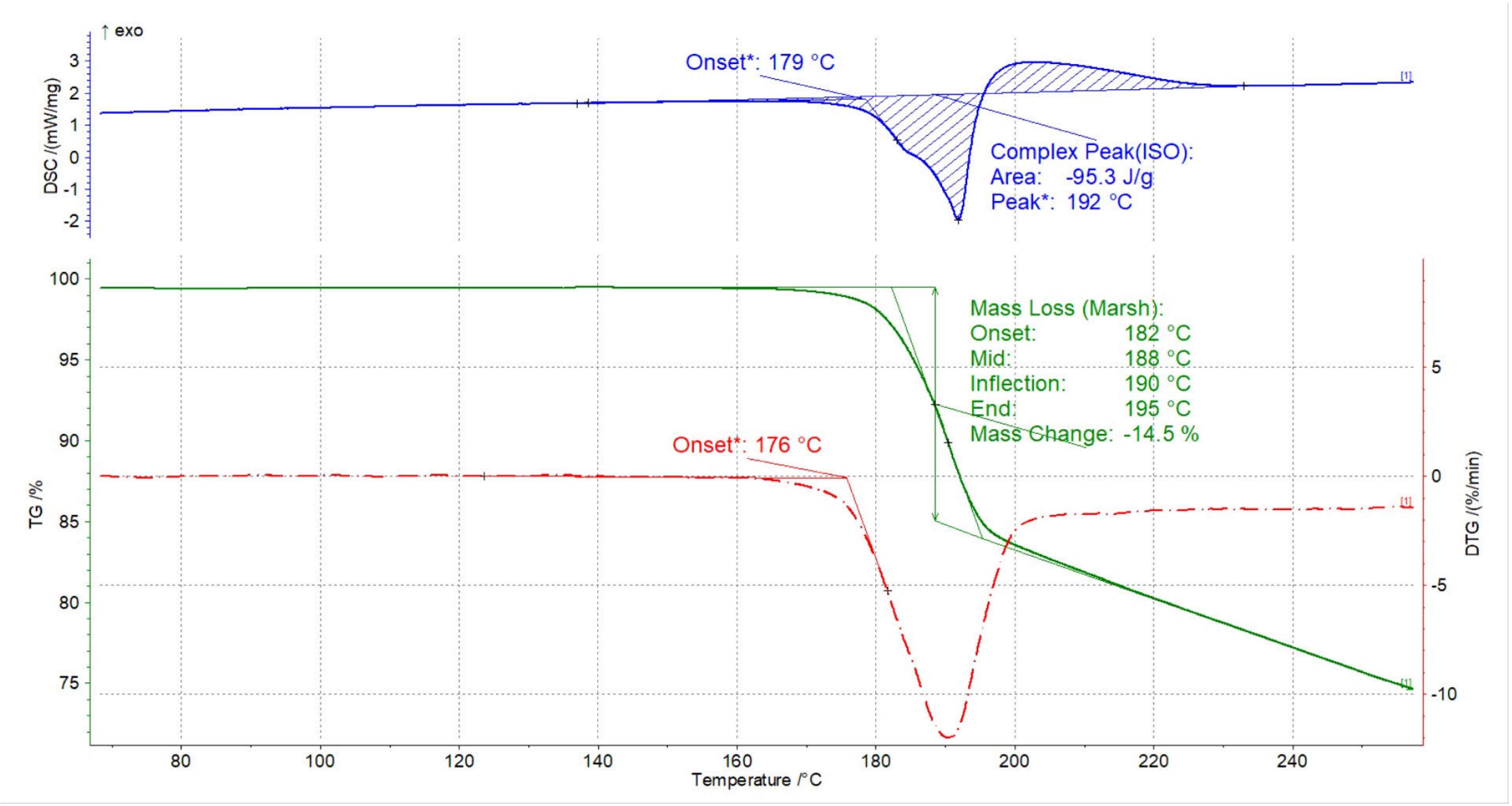

Figure S3. Thermal analysis of betaine **3aa** (rate 10K/min, Ar atmosphere)

Table S1. Optimization of the thermolysis of betaine 3aa

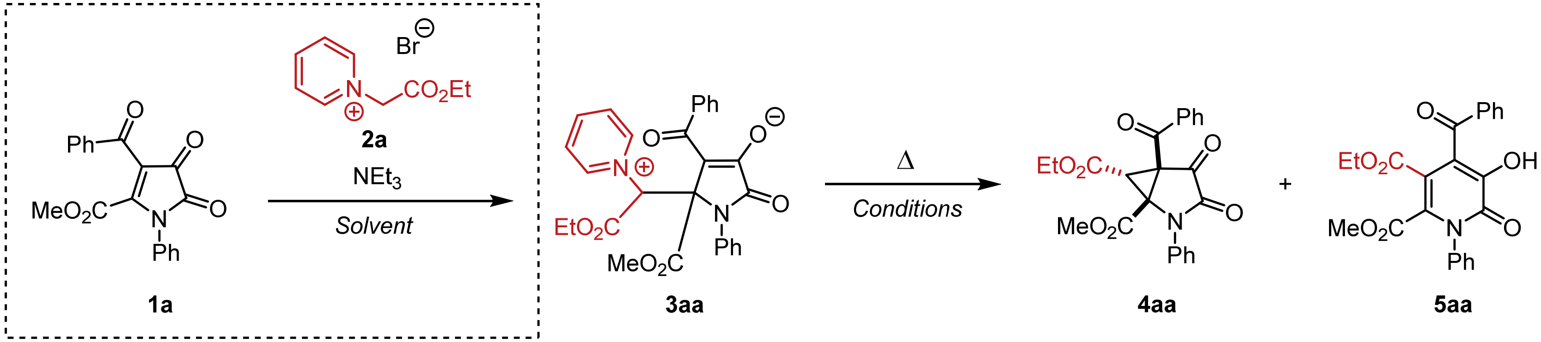

Entry	Solvent (boiling point, °C)	Concentration, M	The reaction was performed in	Temperature, °C	Time	Yield [a], % Cyclopropane 4aa	Pyridine-2,3-dione 5aa
1	1,4-Dioxane (101)	0.05	Flask	Reflux	2h	24	—
2	PhCl (132)	0.05	Flask	Reflux	1h	72	—
		0.05			2h	69	—
3	HFIP (59)	0.05	Flask	Reflux	1h	**3aa**	
4	*i*-AmOH (131)	0.05	Flask	Reflux	1h	*Complex mixture*	
5	AcOH (118)	0.05	Flask	Reflux	1h	*Complex mixture*	
6	Dimethyl carbonate (90)	0.05	Flask	Reflux	1h	2	—
7	PhOMe (154)	0.05	Flask	Reflux	1h	55	—
					2h	44	—
8	BuOAc (126)	0.05	Flask	Reflux	1h	39	—
					2h	59	—
9	Methyl isobutyl ketone (116)	0.05	Flask	Reflux	1h	30	—
					2h	44	—
10	*N,N*-Dimethylacetamide (165)	0.05	Flask	Reflux	1h	*Complex mixture*	
11	*o*-DCB (181)	0.05	Flask	Reflux	1h	28	20
					2h	8	37
12	*p*-Xylene (138)	0.05	Flask	Reflux	1h	69	—

					2h	68	—
13 [b]	PhCl (132)	0.05	Flask	Reflux	1h	*Complex mixture*	
14	*o*-DCB (181)	0.05	Flask	140	1h	65	—
				190	1h	—	38
15 [b]	*o*-DCB (181)	0.05	Flask	140	1h	—	–
				190	1h	—	19
16	PhCl (132)	0.2	V-vial	140	1h	71	—
17	*o*-DCB (181)	0.2	V-vial	190	4min	65	—
					10min	43	13
					30min	23	21
					1h	—	37
					2h	—	31
					3h	—	30
					4h	—	28
					5h	—	26
18 [c]	PhCl (132)	0.2	V-vial	140	40min	83	—
19 [d]	*o*-DCB (181)	0.2	V-vial	190	30min	49	18
					1h	22	30
20	*p*-Xylene (138)	0.2	V-vial	140	1.5h	78	—
21	PhCN (191)	0.2	V-vial	190	2min	55	—
					30min	*Complex mixture*	
22	$(CO_2Et)_2$ (185)	0.2	V-vial	190	2min	30	—
					30min	6	14
23 [e]	PhCl (132)	0,2	V-vial	140	0min	99	—
					20min	98	—
					12h	72	18
24 [f]	PhCl (132)	0.2	V-vial	140		—	—
25 [g]	PhCl (132)	0.2	V-vial	140	25min	67	—

26 [h]	PhCl (132)	0.2	V-vial	190	1h	—	96
					2h	—	91
27	*o*-DCB (181)	0.2	V-vial	165	20min	49	7
					1h	29	18
28 [f]	*o*-DCB (181)	0.2	V-vial	190	30 min	21	36
29 [g]	*o*-DCB (181)	0.2	V-vial	190	30 min	—	11
30 [i]	*o*-DCB (181)	0.2	V-vial	190	10 min	60	13
					30 min	27	35
31 [j]	*o*-DCB (181)	0.2	V-vial	190 (oil bath)	30 min	4	35
32	Pyridine (115)	0.2	V-vial	115	30 min	7	7
33	Ethylene glycol (197)	0.2	V-vial	190	2 min	6	—
34	Dodecane (216)	0.2	V-vial	190	10 min	34	8

[a] Based on HPLC analysis with Ph_3CH as the internal standard; [b] starting from 1*H*-pyrrol-2,3-dione **1a** without isolation of betaine **3aa**; [c] the vial was removed from the heating block when starting betaine **3aa was** completely dissolved; [d] starting from cyclopropane **4aa**; [e] stability test of cyclopropane **4aa** in PhCl at 140 °C; [f] with 1 equiv. of TSA·H_2O; [g] with 1 equiv. of AcOH; [h] stability test of pyridine-2,3-dione **5aa** in *o*-DCB at 190 °C; [i] under argon; [j] reaction in pressure tube under argon (oil bath); *o*-DCB – 1,2-dichlorobenzene

General procedures for the synthesis of compounds 3, 4 and 5

General Procedure A—synthesis of betaines 3: To a round-bottom flask containing a magnetic stir bar and pyrrole-2,3-dione **1** (1 equiv.), dry acetonitrile (10 mL per 1 mmol of **1**) is added, followed by salt **2** (1.1 equiv.) and triethylamine (1.1 equiv.). The resulting mixture is stirred at room temperature for 1–2 hours. The formed precipitate is filtered and washed with acetonitrile.

General Procedure B—synthesis of cyclopropanes 4: To a V-vial containing a magnetic stir bar and betaine **3** (1 equiv.), dry chlorobenzene (5 mL per 1 mmol of **3**) is added. The vial is connected to a Vigreux column (serving as a condenser) and placed in a preheated-to-140 °C metal heating block. The resulting mixture is stirred at 140 °C until the complete dissolution of the starting betaine **3** (5–50 min, depending on the betaine used). The solution is cooled, and cyclopropanes **4** are isolated by flash-column chromatography (loaded directly on a column in PhCl) or crystallization.

General Procedure C—synthesis of pyridine-2,3-diones 5: To a V-vial containing a magnetic stir bar and betaine **3** (1 equiv.), dry chlorobenzene (5 mL per 1 mmol of **3**) is added. The vial is connected to a Vigreux column (serving as a condenser) and placed in a preheated-to-140 °C metal heating block. The resulting mixture is stirred at 140 °C until the complete consumption of cyclopropane **4** (TLC or HPLC control). The solution is cooled, PhCl evaporated under reduced pressure and the residue crystallized from EtOH/petroleum ether to obtain, after filtration and washing with cold EtOH, pyridine-2,3-diones **5**.

(*S)-4-benzoyl-5-((*R**)-2-ethoxy-2-oxo-1-(pyridin-1-ium-1-yl)ethyl)-5-(methoxycarbonyl)-2-oxo-1-phenyl-2,5-dihydro-1*H*-pyrrol-3-olate (3aa)**

Synthesized according to **General Procedure A** from pyrrole-2,3-dione **1a** (1 mmol scale) and salt **2a**. A pale-yellow/off-white powder, 376 mg (75%), m.p. 166–168 °C (dec.).^{1}**H NMR** (400 MHz, DMSO-*d*6): δ 8.95 – 8.84 (m, 2H), 8.64 – 8.54 (m, 1H), 8.05 (t, *J* = 7.1 Hz, 2H), 7.50 (dd, *J* = 8.7, 6.7 Hz, 2H), 7.43 – 7.33 (m, 3H), 7.33 – 7.24 (m, 1H), 7.24 – 7.11 (m, 5H), 3.82 (s, 3H), 3.65 (dq, *J* = 10.7, 7.1 Hz, 1H), 3.05 (dq, *J* = 10.7, 7.1 Hz, 1H), 0.97 (t, *J* = 7.1 Hz, 3H). ^{13}**C NMR** (101 MHz, DMSO-*d*6): δ 184.6, 169.4, 169.1, 167.4, 164.9, 147.0, 146.5 (2C), 140.6, 135.7, 129.1, 128.7 (2C), 127.9 (2C), 127.5, 127.3 (2C), 126.5 (4C), 106.1, 71.6, 70.8, 62.1, 52.8, 13.1. **IR** (mineral oil), cm^{-1}: 1741, 1729, 1713, 1654, 1632, 1582. Anal. Calcd for $C_{28}H_{24}N_2O_7$: C 67.19; H 4.83; N 5.60. Found: C 67.46; H 4.78; N 5.81.

(*S)-4-benzoyl-1-(4-chlorophenyl)-5-((*R**)-2-ethoxy-2-oxo-1-(pyridin-1-ium-1-yl)ethyl)-5-(methoxycarbonyl)-2-oxo-2,5-dihydro-1*H*-pyrrol-3-olate (3ba)**

Synthesized according to **General Procedure A** from pyrrole-2,3-dione **1b** (0.5 mmol scale) and salt **2a**. An off-white powder, 192 mg (72%), m.p. 167–175 °C (dec.). ^{1}**H NMR** (400 MHz, DMSO-*d*6), mixture of diastereomers, d.r. ~3.8 : 1 (A : B): δ 9.04 (d, *J* = 5.7 Hz, 0.42H, B), 8.89 (d, *J* = 5.6 Hz, 1.58H, A), 8.59 (t, *J* = 7.8 Hz, 1H), 8.08 – 8.00 (m, 2H), 7.82 (d, *J* = 7.0 Hz, 0.42H, B), 7.57 (d, *J* = 8.8 Hz, 1.58H, A), 7.46 – 7.14 (m, 7.37H, A+B), 6.79 (d, *J* = 8.8 Hz, 0.42H, B), 6.52 (s, 0.21H, B), 4.04 – 3.88 (m, 0.42H, B), 3.81 (s, 2.37H, A), 3.80 (s, 0.63H, B), 3.72 (dq, *J* = 10.7, 7.1 Hz, 0.79H, A), 3.19 (dq, *J* = 10.8, 7.2 Hz, 0.79H, A), 1.10 (t, *J* = 7.1 Hz, 0.63H, B), 1.01 (t, *J* = 7.1

Hz, 2.37H, A). **^{13}C NMR** (101 MHz, DMSO-*d*$_6$), A (major): δ 184.6, 169.4, 169.1, 167.0, 164.9, 147.0, 146.6 (2C), 140.5, 134.7, 132.1, 129.2, 129.1 (2C), 128.7 (2C), 127.9 (2C), 126.5 (4C), 106.0, 71.5, 70.7, 62.2, 53.0, 13.0. **IR** (mineral oil), cm^{-1}:1758, 1737, 1714, 1650, 1627, 1580, 1529. Anal. Calcd for $C_{28}H_{23}ClN_2O_7$: C 62.87; H 4.33; N 5.24. Found: C 63.10; H 4.27; N 5.35.

(*S)-4-(4-bromobenzoyl)-5-((*R**)-2-ethoxy-2-oxo-1-(pyridin-1-ium-1-yl)ethyl)-5-(methoxycarbonyl)-2-oxo-1-(*p*-tolyl)-2,5-dihydro-1*H*-pyrrol-3-olate (3ca)**

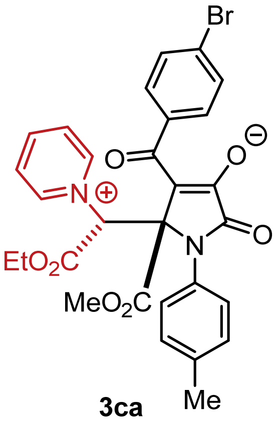

3ca

Synthesized according to **General Procedure A** from pyrrole-2,3-dione **1c** (1 mmol scale) and salt **2a**. A pale-yellow powder, 408 mg (69%), m.p. 188–189 °C (dec.). **^{1}H NMR** (400 MHz, DMSO-*d*$_6$): δ 8.87 (d, *J* = 5.7 Hz, 2H), 8.59 (tt, *J* = 7.8, 1.3 Hz, 1H), 8.04 (t, *J* = 7.1 Hz, 2H), 7.43 – 7.34 (m, 2H), 7.32 – 7.25 (m, 2H), 7.27 – 7.18 (m, 2H), 7.18 – 7.06 (m, 3H), 3.79 (s, 3H), 3.68 (dq, *J* = 10.7, 7.1 Hz, 1H), 3.11 (dq, *J* = 10.7, 7.2 Hz, 1H), 2.35 (s, 3H), 0.98 (t, *J* = 7.1 Hz, 3H).**^{13}C NMR** (101 MHz, DMSO-*d*$_6$): δ 182.9, 169.4, 168.9, 167.9, 164.8, 147.0, 146.4 (2C), 139.7, 137.2, 133.0, 130.0 (2C), 129.6 (2C), 129.2 (2C), 127.2 (2C), 126.5 (2C), 122.5, 105.9, 71.4, 70.7, 62.2, 52.8, 20.5, 13.0. **IR** (mineral oil), cm^{-1}: 1743, 1728, 1712, 1648, 1632, 1571, 1511. Anal. Calcd for $C_{29}H_{25}BrN_2O_7$: C 58.70; H 4.25; N 4.72. Found: C 59.08; H 4.27; N 5.01.

(*S)-4-cinnamoyl-5-((*R**)-2-ethoxy-2-oxo-1-(pyridin-1-ium-1-yl)ethyl)-5-(ethoxycarbonyl)-2-oxo-1-phenyl-2,5-dihydro-1*H*-pyrrol-3-olate (3da)**

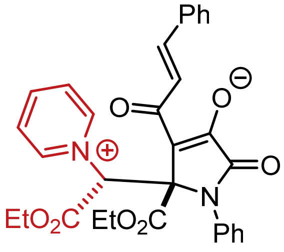

3da

Synthesized according to **General Procedure A** from pyrrole-2,3-dione **1d** (1 mmol scale) and salt **2a**. A light-yellow powder, 390 mg (72%), m.p. 186–188 °C (dec.). **^{1}H NMR** (400 MHz, DMSO-*d*$_6$): δ 8.86 (d, *J* = 5.3 Hz, 2H), 8.57 (t, *J* = 7.8 Hz, 1H), 8.05 (t, *J* = 7.3 Hz, 2H), 8.00 (d, *J* = 16.0 Hz, 1H), 7.53 – 7.28 (m, 11H), 7.18 (d, *J* = 15.9 Hz, 1H), 4.33 – 4.20 (m, 2H), 3.64 (dq, *J* = 10.7, 7.1 Hz, 1H), 3.07 (dq, *J* = 10.8, 7.1 Hz, 1H), 1.20 (t, *J* = 7.1 Hz, 3H), 0.97 (t, *J* = 7.1 Hz, 3H). **^{13}C NMR** (101 MHz, DMSO-*d*$_6$): δ 178.4, 169.3, 168.8, 168.3, 164.9, 147.0, 146.3 (2C), 135.8, 135.8, 135.7, 128.8, 128.7 (2C), 128.6 (2C), 127.5, 127.3 (2C), 127.2 (2C), 127.1, 126.4 (2C), 108.1, 71.5, 70.7, 62.1, 61.9, 13.7, 13.0. **IR** (mineral oil), cm^{-1}: 1751, 1731, 1715, 1634. Anal. Calcd for $C_{31}H_{28}N_2O_7$: C 68.88; H 5.22; N 5.18. Found: C 69.19; H 5.34; N 5.10.

(*S)-5-((*R**)-2-ethoxy-2-oxo-1-(pyridin-1-ium-1-yl)ethyl)-4,5-bis(methoxycarbonyl)-2-oxo-1-phenyl-2,5-dihydro-1*H*-pyrrol-3-olate (3ea)**

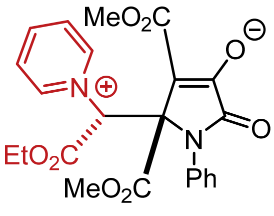

3ea

Synthesized according to **General Procedure A** from pyrrole-2,3-dione **1e** (1 mmol scale) and salt **2a**. An off-white powder, 265 mg (58%), m.p. 152–154 °C (dec.). **^{1}H NMR** (400 MHz, DMSO-*d*$_6$): δ 8.88 – 8.82 (m, 2H), 8.65 (tt, *J* = 7.8, 1.3 Hz, 1H), 8.13 (t, *J* = 7.4 Hz, 2H), 7.49 – 7.43 (m, 2H), 7.40 – 7.32 (m, 3H), 7.11 (s, 1H), 3.79 (s, 3H), 3.60 (dq, *J* = 10.8, 7.1 Hz, 1H), 3.31 (s, 3H), 3.04 (dq, *J* = 10.8, 7.1 Hz, 1H), 0.95 (t, *J* = 7.1 Hz, 3H). **^{13}C NMR** (101 MHz, DMSO-*d*$_6$): δ 169.2, 169.1, 166.3, 164.8, 164.2, 147.2, 146.2 (2C), 135.8, 128.6 (2C), 127.3, 127.2 (2C), 126.7 (2C), 92.4, 71.0, 70.7, 62.1, 52.9, 49.0,

13.0. **IR** (mineral oil), cm^{-1}: 1743, 1712, 1655, 1629, 1607, 1589. Anal. Calcd for $C_{23}H_{22}N_2O_8$: C 60.79; H 4.88; N 6.16. Found: C 60.91; H 4.92; N 6.10.

(*S)-5-((*R**)-2-ethoxy-2-oxo-1-(pyridin-1-ium-1-yl)ethyl)-1-(4-methoxyphenyl)-4,5-bis(4-methylbenzoyl)-2-oxo-2,5-dihydro-1*H*-pyrrol-3-olate (3fa)**

Synthesized according to **General Procedure A** from pyrrole-2,3-dione **1f** (1 mmol scale) and salt **2a**. A light-yellow powder, 227 mg (37%), m.p. 146–148 °C (dec.). **^{1}H NMR** (400 MHz, DMSO-*d*6): δ 8.94 (d, *J* = 5.3 Hz, 2H), 8.55 (tt, *J* = 7.8, 1.3 Hz, 1H), 8.05 – 7.97 (m, 2H), 7.61 (d, *J* = 8.3 Hz, 2H), 7.25 – 7.13 (m, 5H), 7.05 – 6.92 (m, 6H), 3.83 – 3.76 (m, 1H), 3.75 (s, 3H), 3.38 (dq, *J* = 10.8, 7.1 Hz, 1H), 2.29 (s, 3H), 2.24 (s, 3H), 1.03 (t, *J* = 7.1 Hz, 3H). **^{13}C NMR** (101 MHz, DMSO-*d*6): δ 192.2, 184.2, 169.0, 168.0, 165.1, 157.9, 146.7, 146.5 (2C), 146.2, 142.2, 138.7, 137.7, 133.9, 128.7 (2C), 127.9 (2C), 127.9 (2C), 127.7 (2C), 127.1 (2C), 126.3 (2C), 113.8 (2C), 105.2, 75.9, 71.2, 62.3, 55.3, 20.8 (2C), 13.1. **IR** (mineral oil), cm^{-1}: 1752, 1737, 1710, 1680, 1647, 1605, 1571. Anal. Calcd for $C_{36}H_{32}N_2O_7$: C 71.51; H 5.33; N 4.63. Found: C 71.66; H 5.54; N 4.47.

(*S)-4,5-dibenzoyl-5-((*R**)-2-ethoxy-2-oxo-1-(pyridin-1-ium-1-yl)ethyl)-2-oxo-1-(*p*-tolyl)-2,5-dihydro-1*H*-pyrrol-3-olate (3ga)**

Synthesized according to **General Procedure A** from pyrrole-2,3-dione **1g** (0.94 mmol scale) and salt **2a**. An off-white powder, 247 mg (48%), m.p. 151–153 °C (dec.). **^{1}H NMR** (400 MHz, DMSO-*d*6): δ 8.97 (d, *J* = 5.2 Hz, 2H), 8.57 (tt, *J* = 7.8, 1.3 Hz, 1H), 8.07 – 8.02 (m, 2H), 7.69 – 7.62 (m, 2H), 7.52 – 7.46 (m, 1H), 7.38 – 7.32 (m, 2H), 7.27 – 7.19 (m, 6H), 7.17 – 7.09 (m, 2H), 7.04 – 6.98 (m, 2H), 3.77 (dq, *J* = 10.8, 7.1 Hz, 1H), 3.35 (dq, *J* = 10.8, 7.1 Hz, 1H), 2.30 (s, 3H), 1.02 (t, *J* = 7.1 Hz, 3H). **^{13}C NMR** (101 MHz, DMSO-*d*6): δ 192.8, 184.4, 168.8, 168.1, 165.0, 146.8, 146.5 (2C), 140.6, 136.7, 136.6, 132.7, 131.9, 129.1 (2C), 129.0, 128.2 (2C), 127.7 (2C), 127.6 (2C), 126.5 (2C), 126.4 (2C), 126.0 (2C), 104.9, 75.6, 71.1, 62.3, 20.4, 13.1. **IR** (mineral oil), cm^{-1}: 1747, 1719, 1681, 1647, 1597, 1581. Anal. Calcd for $C_{34}H_{28}N_2O_6$: C 72.85; H 5.03; N 5.00. Found: C 73.12; H 5.09; N 4.96.

(*S)-4-benzoyl-5-(methoxycarbonyl)-2-oxo-1-phenyl-5-((*R**)-(3-phenyl-1,2,4-oxadiazol-5-yl)(pyridin-1-ium-1-yl)methyl)-2,5-dihydro-1*H*-pyrrol-3-olate (3ab)**

Synthesized according to **General Procedure A** from pyrrole-2,3-dione **1a** (1 mmol scale) and salt **2b**. An off-white powder, 444 mg (78%), m.p. 173–175 °C (dec.). **^{1}H NMR** (400 MHz, DMSO-*d*6), mixture of diastereomers, d.r. ~33 : 1 (A : B): δ 9.26 (d, *J* = 6.5 Hz, 0.06H, B), 9.17 (d, *J* = 5.8 Hz, 1.94H, A), 8.65 (t, *J* = 7.8 Hz, 0.97H, A), 8.61 (t, *J* = 7.7 Hz, 0.03H, B), 8.12 (t, *J* = 7.0 Hz, 1.94H, A), 8.02 (t, *J* = 7.2 Hz, 0.06H, B), 7.97 (s, 1H), 7.74 (d, *J* = 6.8 Hz, 2H), 7.64 – 7.50 (m, 3H), 7.39 – 7.30 (m, 3H), 7.28 – 7.18 (m, 4H), 7.13 – 7.02 (m, 2.94H), 6.88 (d, *J* = 7.8 Hz, 0.06H, B), 3.86 (s, 2.91H, A), 3.83 (s,

0.09H, B). **^{13}C NMR** (101 MHz, DMSO-d_6), A (major): δ 184.8, 172.2, 170.2, 168.7, 167.4, 167.0, 147.7, 146.4 (2C), 140.1, 135.3, 131.6, 129.4, 129.0 (2C), 128.7 (2C), 128.1 (2C), 127.1 (2C), 127.0 (2C), 126.8, 126.7 (2C), 125.5 (2C), 125.1, 105.5, 72.2, 65.8, 53.1.**IR** (mineral oil), cm^{-1}: 1743, 1732, 1651, 1629, 1582. Anal. Calcd for $C_{33}H_{24}N_4O_6$: C 69.22; H 4.23; N 9.79. Found: C 69.51; H 4.45; N 9.71.

(*S)-4-benzoyl-5-((*R**)-cyano(pyridin-1-ium-1-yl)methyl)-5-(methoxycarbonyl)-2-oxo-1-phenyl-2,5-dihydro-1*H*-pyrrol-3-olate (3ac)**

Synthesized according to **General Procedure A** from pyrrole-2,3-dione **1a** (1 mmol scale) and salt **2c**. A pale-beige powder, 370 mg (82%), m.p. 180–182 °C (dec.). **^{1}H NMR** (400 MHz, DMSO-d_6), mixture of diastereomers, d.r. ~50 : 1, A (major): δ 8.91 (d, *J* = 5.5 Hz, 2H), 8.67 (tt, *J* = 7.9, 1.3 Hz, 1H), 8.12 (dd, *J* = 7.8, 6.7 Hz, 2H), 7.61 – 7.54 (m, 4H), 7.51 – 7.45 (m, 1H), 7.42 – 7.28 (m, 5H), 7.27 (s, 1H), 3.77 (s, 3H). **^{13}C NMR** (101 MHz, DMSO-d_6), A (major): δ 184.9, 170.0, 168.3, 166.9, 148.3, 145.1 (2C), 139.5, 134.8, 129.8, 129.5 (2C), 128.5 (2C), 128.3, 127.7 (2C), 127.2 (2C), 126.8 (2C), 113.9, 103.6, 71.2, 63.2, 53.1. **IR** (mineral oil), cm^{-1}:1734, 1714, 1653, 1629, 1595, 1581 (CN-peak was not observed even in KBr-tablet). Anal. Calcd for $C_{26}H_{19}N_3O_5$: C 68.87; H 4.22; N 9.27. Found: C 69.16; H 4.13; N 9.26.

(5-(Cyano(pyridin-1-ium-1-yl)methyl)-4,5-bis(methoxycarbonyl)-2-oxo-1-phenyl-2,5-dihydro-1*H*-pyrrol-3-olate (3ec)

Synthesized according to **General Procedure A** from pyrrole-2,3-dione **1e** (0.4 mmol scale) and salt **2c**. A pale-beige powder, 110 mg (68%), m.p. 176–178 °C (dec.). **^{1}H NMR** (400 MHz, DMSO-d_6), mixture of diastereomers, d.r. ~9 : 1 (A : B): δ 9.08 (d, *J* = 5.5 Hz, 1.8H, A), 8.87 (d, *J* = 5.5 Hz, 0.2H, B), 8.73 (tt, *J* = 7.9, 1.3 Hz, 0.1H, B), 8.64 (tt, *J* = 7.8, 1.3 Hz, 0.9H, A), 8.20 (dd, *J* = 7.8, 6.7 Hz, 0.2H, B), 8.15 – 8.06 (m, 1.8H, A), 7.58 – 7.53 (m, 0.2H, B), 7.52 – 7.39 (m, 2.8H, A + B), 7.28 – 7.25 (m, 0.2H, B), 7.24 – 7.17 (m, 1.8H, A), 7.08 (s, 0.1H, B), 6.64 (s, 0.9H, A), 3.78 (s, 2.7H, A), 3.74 (s, 0.3H, B), 3.33 (s, 0.3H, B), 3.14 (s, 2.7H, A). 13C spectral signals are listed together for both diastereomers due to rapid epimerization in solution (see NMR ^{1}H spectrum recorded 30 min after dissolution; d.r. ~1 : 1.5 (A : B)). **^{13}C NMR** (101 MHz, DMSO-d_6), A + B: δ 170.2, 170.1, 168.0, 167.9, 167.0, 166.5, 164.9, 164.6, 148.3, 147.6, 145.8, 145.3, 135.4, 134.8, 129.5, 129.4, 128.4, 128.1, 127.6, 127.6, 127.1, 126.9, 113.9, 113.1, 91.3, 89.9, 71.1, 70.9, 65.0, 64.0, 53.2, 53.0, 49.5, 49.1. **IR** (mineral oil), cm^{-1}: 1748, 1723, 1694, 1679, 1634 (CN-peak was not observed even in KBr-tablet). Anal. Calcd for $C_{21}H_{17}N_3O_6$: C 61.92; H 4.21; N 10.31. Found: C 61.70; H 4.05; N 9.98.

(*S)-4-benzoyl-5-((*R**)-1-(3-carbamoylpyridin-1-ium-1-yl)-2-ethoxy-2-oxoethyl)-5-(methoxycarbonyl)-2-oxo-1-phenyl-2,5-dihydro-1*H*-pyrrol-3-olate (3ad)**

Synthesized according to **General Procedure A** from pyrrole-2,3-dione **1a** (0.5 mmol scale) and salt **2d**. An off-white powder, 197 mg (72%), m.p. 148–150 °C (dec.). ^{1}**H NMR** (400 MHz, DMSO-d_6): δ 9.34 (s, 1H), 9.03 (d, *J* = 6.3 Hz, 1H), 8.97 (d, *J* = 8.2 Hz, 1H), 8.43 (s, 1H), 8.18 (dd, *J* = 8.1, 6.3 Hz, 1H), 8.04 (s, 1H), 7.54 – 7.47 (m, 2H), 7.42 – 7.34 (m, 3H), 7.31 – 7.26 (m, 1H), 7.24 – 7.15 (m, 5H), 3.79 (s, 3H), 3.71 (dq, *J* = 10.8, 7.1 Hz, 1H), 3.26 – 3.18 (m, 1H), 1.01 (t, *J* = 7.1 Hz, 3H). ^{13}**C NMR** (101 MHz, DMSO-d_6): δ 184.6, 169.4, 168.8, 167.5, 164.6, 162.1, 144.4, 140.6, 135.6, 131.7, 129.1, 128.8 (2C), 127.9 (2C), 127.5, 126.8 (2C), 126.6 (2C), 126.2, 105.6, 71.4, 71.3, 62.3, 52.9, 13.1. **IR** (mineral oil), cm^{-1}: 3404, 3302, 1754, 1746, 1710, 1688, 1645, 1634, 1581. Anal. Calcd for $C_{29}H_{25}N_3O_8$: C 64.08; H 4.64; N 7.73. Found: C 64.29; H 4.75; N 7.63.

(*S)-4-benzoyl-5-((*R**)-1-(4-benzoylpyridin-1-ium-1-yl)-2-ethoxy-2-oxoethyl)-5-(methoxycarbonyl)-2-oxo-1-phenyl-2,5-dihydro-1*H*-pyrrol-3-olate (3ae)**

Synthesized according to **General Procedure A** from pyrrole-2,3-dione **1a** (0.5 mmol scale) and salt **2e**. A beige powder, 253 mg (84%), m.p. 145–148 °C (dec.). ^{1}**H NMR** (400 MHz, DMSO-d_6), mixture of diastereomers, d.r. ~30 : 1 (major): δ 9.18 (d, *J* = 7.1 Hz, 2H), 8.21 (d, *J* = 6.9 Hz, 2H), 7.76 (tt, *J* = 7.2, 1.4 Hz, 1H), 7.61 – 7.47 (m, 6H), 7.42 – 7.31 (m, 6H), 7.28 – 7.20 (m, 3H), 3.80 (s, 3H), 3.74 (dq, *J* = 10.7, 7.1 Hz, 1H), 3.25 – 3.17 (m, 1H), 0.99 (t, *J* = 7.1 Hz, 3H). ^{13}**C NMR** (101 MHz, DMSO-d_6), A (major): δ 191.5, 184.6, 169.7, 169.0, 167.7, 164.6, 152.8, 147.6 (2C), 140.2, 135.7, 134.6, 133.8, 129.9 (2C), 129.4, 128.9 (2C), 128.8 (2C), 128.2 (2C), 127.4, 126.9 (2C), 126.7 (2C), 125.0 (2C), 106.0, 71.3, 71.1, 62.3, 52.9, 13.1. **IR** (mineral oil), cm^{-1}: 1747, 1734, 1701, 1675, 1641, 1595, 1582, 1513. Anal. Calcd for $C_{35}H_{28}N_2O_8$: C 69.53; H 4.67; N 4.63. Found: C 69.20; H 4.79; N 4.61.

(*S)-4-benzoyl-5-((*R**)-2-ethoxy-1-(3-methylpyridin-1-ium-1-yl)-2-oxoethyl)-5-(methoxycarbonyl)-2-oxo-1-phenyl-2,5-dihydro-1*H*-pyrrol-3-olate (3af)**

Synthesized according to **General Procedure A** from pyrrole-2,3-dione **1a** (0.5 mmol scale) and salt **2f**. An off-white powder, 98 mg (38%), m.p. 172–174 °C (dec.). ^{1}**H NMR** (400 MHz, DMSO-d_6): δ 8.78 (d, *J* = 6.3 Hz, 1H), 8.70 (s, 1H), 8.44 – 8.41 (m, 1H), 8.02 – 7.97 (m, 1H), 7.55 – 7.45 (m, 2H), 7.42 – 7.18 (m, 8H), 7.01 (s, 1H), 3.79 (s, 3H), 3.72 (dq, *J* = 10.7, 7.1 Hz, 1H), 3.26 – 3.18 (m, 1H), 2.24 (s, 3H), 0.99 (t, *J* = 7.1 Hz, 3H). ^{13}**C NMR** (101 MHz, DMSO-d_6), A (major): δ 184.5, 169.6, 169.1, 167.3, 164.8, 147.2, 145.9, 143.3, 140.4, 136.7, 135.7, 129.3, 128.8 (2C), 128.0 (2C), 127.4, 126.9 (2C), 126.6 (2C), 126.1, 105.9, 71.4, 70.8, 62.2, 52.7, 17.5, 13.1. **IR** (mineral oil), cm^{-1}: 1740, 1732, 1725, 1631, 1584, 1534, 1518. Anal. Calcd for $C_{29}H_{26}N_2O_7$: C 67.70; H 5.09; N 5.44. Found: C 67.82; H 4.95; N 5.49.

(*S)-4-benzoyl-5-((*R**)-2-ethoxy-1-(isoquinolin-2-ium-2-yl)-2-oxoethyl)-5-(methoxycarbonyl)-2-oxo-1-phenyl-2,5-dihydro-1*H*-pyrrol-3-olate (3ag)**

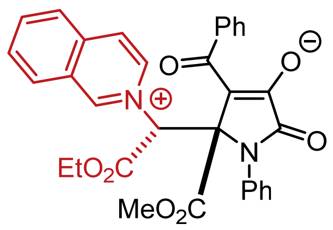

3ag

Synthesized according to **General Procedure A** from pyrrole-2,3-dione **1a** (0.5 mmol scale) and salt **2g**. A pale-yellow powder, 198 mg (72%), m.p. 175–177 °C (dec.). **^{1}H NMR** (400 MHz, DMSO-*d*$_6$): δ 9.86 (s, 1H), 8.66 (d, *J* = 7.6 Hz, 1H), 8.46 (d, *J* = 7.0 Hz, 1H), 8.39 (d, *J* = 8.4 Hz, 1H), 8.30 (d, *J* = 8.3 Hz, 1H), 8.22 (ddd, *J* = 8.2, 6.8, 1.2 Hz, 1H), 7.96 – 7.91 (m, 1H), 7.56 – 7.49 (m, 2H), 7.44 – 7.35 (m, 3H), 7.24 – 7.11 (m, 2H), 7.10 – 6.97 (m, 4H), 3.80 (s, 3H), 3.78 – 3.67 (m, 1H), 0.99 (t, *J* = 7.1 Hz, 3H). The signal of one diastereotopic atom of the CH_2 group is in the water region in ^{1}H NMR. **^{13}C NMR** (101 MHz, DMSO-*d*$_6$): δ 169.8, 169.1, 137.7, 135.8, 130.8, 129.1, 128.8, 127.8, 127.4, 127.0, 126.3, 125.8, 123.8, 71.5, 70.9, 62.2, 52.8, 13.1. Some of the carbon atom signals are not visible due to the very low solubility of the compound. **IR** (mineral oil), cm^{-1}: 1744, 1732, 1714, 1646. Anal. Calcd for $C_{32}H_{26}N_2O_7$: C 69.81; H 4.76; N 5.09. Found: C 70.17; H 4.81; N 4.86.

6-Ethyl 1-methyl (1*R,5*S**,6*R**)-5-benzoyl-3,4-dioxo-2-phenyl-2-azabicyclo[3.1.0]hexane-1,6-dicarboxylate** (**4aa**)

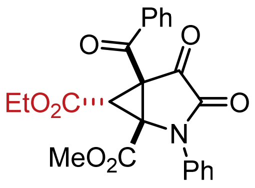

4aa

Synthesized according to **General Procedure B** from betaine **3aa** (0.3 mmol scale). Isolated by FCC, Hexanes/EtOAc 2/1, R_f 0.33. An off-white powder, 102 mg (81%), m.p. 158–160 °C (lit. [9] 159–161 °C). **^{1}H NMR** (400 MHz, $CDCl_3$): δ 7.87 – 7.80 (m, 2H), 7.66 (t, *J* = 7.5 Hz, 1H), 7.60 – 7.49 (m, 4H), 7.48 – 7.41 (m, 2H), 7.30 (t, *J* = 7.4 Hz, 1H), 4.18 (q, *J* = 7.1 Hz, 2H), 4.15 (s, 1H), 3.58 (s, 3H), 1.16 (t, *J* = 7.1 Hz, 3H). **^{13}C NMR** (101 MHz, $CDCl_3$): δ 186.6, 185.1, 165.4, 164.2, 156.7, 136.7, 135.0, 134.2, 129.7 (2C), 129.6 (2C), 129.3 (2C), 127.1, 120.1 (2C), 63.9, 54.8, 54.0, 46.8, 45.7, 13.9. **IR** (mineral oil), cm^{-1}: 1785, 1756, 1742, 1724, 1671, 1595. The spectral data was consistent with the data reported in the literature [9].

6-Ethyl 1-methyl (1*R,5*S**,6*R**)-5-benzoyl-2-(4-chlorophenyl)-3,4-dioxo-2-azabicyclo[3.1.0]hexane-1,6-dicarboxylate** (**4ba**)

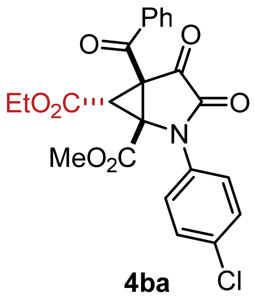

4ba

Synthesized according to **General Procedure B** from betaine **3ba** (0.37 mmol scale). Isolated by crystallization from EtOH. An off-white powder, 128 mg (75%), m.p. 166–169 °C (lit. [9] 167–169 °C). **^{1}H NMR** (400 MHz, $CDCl_3$): δ 7.87 – 7.78 (m, 2H), 7.72 – 7.62 (m, 1H), 7.57 – 7.49 (m, 4H), 7.47 – 7.37 (m, 2H), 4.18 (q, *J* = 7.1 Hz, 2H), 4.13 (s, 1H), 3.60 (s, 3H), 1.17 (t, *J* = 7.1 Hz, 3H). **^{13}C NMR** (101 MHz, $CDCl_3$): δ 186.3, 184.6, 165.3, 164.0, 156.6, 135.2, 135.1, 134.1, 132.7, 129.7 (2C), 129.7 (2C), 129.4 (2C), 121.3 (2C), 64.0, 54.6, 54.1, 46.8, 45.5, 14.0. **IR** (mineral oil), cm^{-1}: 1782, 1758, 1749, 1722, 1674, 1596. The spectral data was consistent with the data reported in the literature [9].

6-Ethyl 1,5-dimethyl (1*R,5*R**,6*R**)-3,4-dioxo-2-phenyl-2-azabicyclo[3.1.0]hexane-1,5,6-tricarboxylate (4ea)**

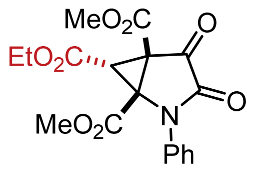

4ea

Synthesized according to **General Procedure B** from betaine **3ea** (0.3 mmol scale). Isolated by FCC, Hexanes/EtOAc 3/2, R_f 0.32. An off-white powder, 102 mg (91%), m.p. 129–131 °C. **^{1}H NMR** (400 MHz, $CDCl_3$): δ 7.50 – 7.46 (m, 2H), 7.45 – 7.39 (m, 2H), 7.30 – 7.25 (m, 1H), 4.16 (s, 1H), 4.12 (qd, *J* = 7.2, 1.0 Hz, 2H), 3.84 (s, 3H), 3.69 (s, 3H), 1.13 (t, *J* = 7.1 Hz, 3H). **^{13}C NMR** (101 MHz, $CDCl_3$): δ 183.6, 164.9, 163.7, 162.7, 156.6, 136.5, 129.6 (2C), 127.2, 120.2 (2C), 63.8, 55.1, 54.2 (2C), 45.4, 41.9, 13.9. **IR** (mineral oil), cm^{-1}: 1777, 1747, 1724, 1597. The spectral data was consistent with the data reported in the literature [9].

Ethyl (1*R,5*S**,6*R**)-2-(4-methoxyphenyl)-1,5-bis(4-methylbenzoyl)-3,4-dioxo-2-azabicyclo[3.1.0]hexane-6-carboxylate (4fa)**

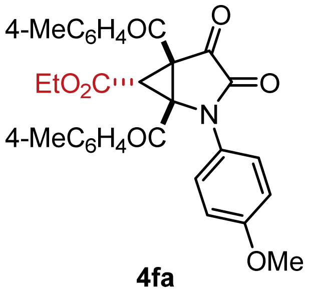

4fa

Synthesized according to **General Procedure B** from betaine **3fa** (0.3 mmol scale). Isolated by FCC, Hexanes/EtOAc 2/1, R_f 0.5. A yellow powder, 90 mg (57%), m.p. 177–179 °C (lit. [9] 178–180 °C). **^{1}H NMR** (400 MHz, $CDCl_3$): δ 7.84 (d, *J* = 8.0 Hz, 2H), 7.64 (d, *J* = 8.0 Hz, 2H), 7.32 (d, *J* = 8.0 Hz, 2H), 7.26 (d, *J* = 9.0 Hz, 2H), 7.12 (d, *J* = 8.0 Hz, 2H), 6.75 (d, *J* = 9.0 Hz, 2H), 4.25 (s, 1H), 4.25 – 4.18 (m, 2H), 3.71 (s, 3H), 2.45 (s, 3H), 2.33 (s, 3H), 1.19 (t, *J* = 7.1 Hz, 3H). **^{13}C NMR** (101 MHz, $CDCl_3$) δ 190.4, 186.3, 185.3, 165.9, 158.2, 156.7, 146.5, 144.9, 132.9, 132.3, 130.6 (2C), 129.9 (2C), 129.3 (2C), 129.2, 128.9 (2C), 122.0 (2C), 114.6 (2C), 63.8, 60.0, 55.5, 49.8, 45.6, 22.0, 21.8, 14.0. **IR** (mineral oil), cm^{-1}: 1778, 1769, 1737, 1678, 1602. The spectral data was consistent with the data reported in the literature [9].

Ethyl (1*R,5*S**,6*R**)-1,5-dibenzoyl-3,4-dioxo-2-(*p*-tolyl)-2-azabicyclo[3.1.0]hexane-6-carboxylate (4ga)**

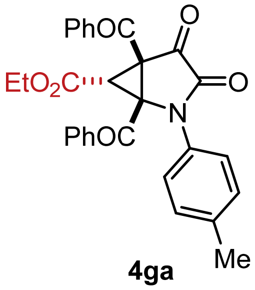

4ga

Synthesized according to **General Procedure B** from betaine **3ga** (0.3 mmol scale). Isolated by FCC, Hexanes/EtOAc 3/1, R_f 0.42. A pale-yellow/off-white powder, 57 mg (40%), m.p. 177–180 °C (dec.). **^{1}H NMR** (400 MHz, $CDCl_3$): δ 7.97 (d, *J* = 7.2 Hz, 2H), 7.72 – 7.65 (m, 3H), 7.56 – 7.50 (m, 2H), 7.49 – 7.42 (m, 1H), 7.34 – 7.28 (m, 2H), 7.20 (d, *J* = 8.5 Hz, 2H), 7.02 (d, *J* = 8.4 Hz, 2H), 4.29 (s, 1H), 4.27 – 4.17 (m, 2H), 2.22 (s, 3H), 1.19 (t, *J* = 7.1 Hz, 3H). **^{13}C NMR** (101 MHz, $CDCl_3$): δ 191.3, 186.9, 185.1, 165.8, 156.6, 137.0, 135.5, 135.2, 134.6, 133.7, 133.6, 130.4 (2C), 130.0 (2C), 129.2 (2C), 128.7 (2C), 128.6 (2C), 120.4 (2C), 63.9, 59.9, 50.0, 45.5, 21.0, 14.0. **IR** (mineral oil), cm^{-1}: 1772, 1764, 1742, 1715, 1685, 1678, 1599, 1577. Anal. Calcd for $C_{29}H_{23}NO_6$: C 72.34; H 4.81; N 2.91. Found: C 72.62; H 4.96; N 2.68.

Methyl (1*R,5*S**,6*R**)-5-benzoyl-3,4-dioxo-2-phenyl-6-(3-phenyl-1,2,4-oxadiazol-5-yl)-2-azabicyclo[3.1.0]hexane-1-carboxylate (4ab)**

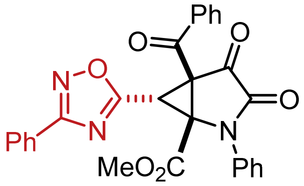

4ab

Synthesized according to **General Procedure B** from betaine **3ab** (0.3 mmol scale). Isolated by FCC, Hexanes/EtOAc 4/1, R_f 0.31. An off-white powder, 104 mg (70%), m.p. 194–196 °C (lit. [9] 195–197 °C). **^{1}H NMR** (400 MHz, $CDCl_3$): δ 7.92 – 7.79 (m, 4H), 7.68 (t, *J* = 7.4 Hz, 1H), 7.53 (t, *J* = 7.7 Hz, 2H), 7.50 – 7.33 (m, 7H), 7.25 – 7.18 (m, 1H), 4.76 (s, 1H), 3.64 (s, 3H). **^{13}C NMR** (101 MHz, $CDCl_3$): δ 186.2, 184.2, 170.7, 168.9, 163.9, 156.7, 136.3, 135.3, 134.1, 132.0, 129.8 (2C), 129.7 (2C), 129.5 (2C), 129.1 (2C), 127.9 (2C), 127.4, 125.3, 120.0 (2C), 55.7, 54.2, 47.1, 38.1. **IR** (mineral oil), cm^{-1}: 1774, 1755, 1739, 1682, 1597, 1579. The spectral data was consistent with the data reported in the literature [9].

Ethyl 5-hydroxy-1-(4-methoxyphenyl)-2,4-bis(4-methylbenzoyl)-6-oxo-1,6-dihydropyridine-3-carboxylate (5fa)

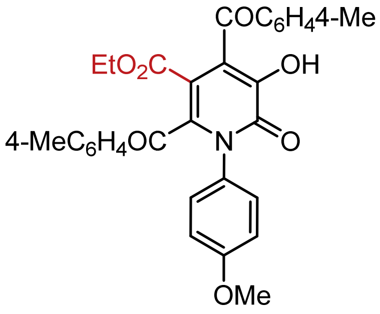

5fa

Synthesized according to **General Procedure C** from betaine **3fa** (0.3 mmol scale). Isolated by crystallization from EtOH. An off-white powder, 73 mg (46%), m.p. 217–219 °C (lit. [9] 218–220 °C). **^{1}H NMR** (400 MHz, $CDCl_3$): δ 7.91 (d, J = 7.9 Hz, 2H), 7.54 (d, J = 8.0 Hz, 2H), 7.32 (d, J = 7.9 Hz, 2H), 7.16 (d, J = 7.9 Hz, 2H), 7.12 – 6.30 (br. m, 5H), 3.84 (q, J = 7.1 Hz, 2H), 3.74 (s, 3H), 2.45 (s, 3H), 2.38 (s, 3H), 0.71 (t, J = 7.1 Hz, 3H). **^{13}C NMR** (101 MHz, $CDCl_3$) δ 191.7, 188.2, 163.3, 160.3, 159.3, 145.2, 144.9, 142.4, 142.3, 134.2, 134.2, 130.2 (2C), 129.6 (2C), 129.6 (2C), 129.5 (2C), 129.1 (2C), 128.1, 125.1, 114.5 (2C), 108.7, 62.1, 55.6, 21.9, 21.9, 13.0. **IR** (mineral oil), cm^{-1}: 3259, 1727, 1686, 1677, 1651, 1606. The spectral data was consistent with the data reported in the literature [9].

Ethyl 2,4-dibenzoyl-5-hydroxy-6-oxo-1-(*p*-tolyl)-1,6-dihydropyridine-3-carboxylate (5ga)

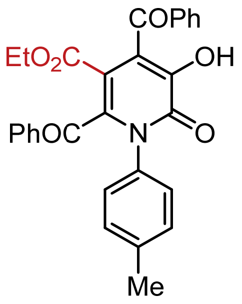

5ga

Synthesized according to **General Procedure C** from betaine **3ga** (0.14 mmol scale). Isolated by crystallization from EtOH. An off-white powder, 18 mg (26%), m.p. 208–209 °C, R_f 0.45 (PhMe/EtOAc 5/1). **^{1}H NMR** (400 MHz, $CDCl_3$): δ 8.06 – 7.99 (m, 2H), 7.68 – 7.58 (m, 3H), 7.58 – 7.47 (m, 3H), 7.37 (t, J = 7.8 Hz, 2H), 7.22 – 6.71 (br. m, 5H), 3.84 (q, J = 7.2 Hz, 2H), 2.27 (s, 3H), 0.70 (t, J = 7.2 Hz, 3H). **^{13}C NMR** (101 MHz, $CDCl_3$): δ 192.0, 188.6, 163.3, 159.0, 142.6, 141.9, 140.1, 136.6, 136.5, 134.0, 133.9, 132.9, 129.9 (2C), 129.4 (2C), 129.0 (2C), 128.9 (2C), 128.8 (4C), 124.8, 108.9, 62.2, 21.3, 13.0. **IR** (mineral oil), cm^{-1}: 3255, 1722, 1693, 1678, 1640, 1600. Anal. Calcd for $C_{29}H_{23}NO_6$: C 72.34; H 4.81; N 2.91. Found: C 72.60; H 4.92; N 2.75.

Crystal structure determination

The unit cell parameters and the X-ray diffraction intensities were measured on an Xcalibur Ruby diffractometer. The empirical absorption correction was introduced by the multi-scan method using the SCALE3 ABSPACK algorithm [5]. Using the Olex2 [6], the structures were solved with the SHELXT [7] program and refined by the full-matrix least squares method in the anisotropic approximation for all non-hydrogen atoms with the SHELXL program [8]. Hydrogen atoms were positioned geometrically and refined using a riding model.

Table S2. Selected bond lengths in compounds **3aa** and **3da**.

Compound	Bond	Bond length, Å
**3aa**	O3–C11	1.252(3)
**3aa**	C11–C3	1.418(3)
**3aa**	C3–C2	1.408(3)
**3aa**	C2–O2	1.231(3)
**3da**	O3–C11	1.253(3)
**3da**	C11–C3	1.426(5)
**3da**	C3–C2	1.399(4)
**3da**	C2–O2	1.247(3)

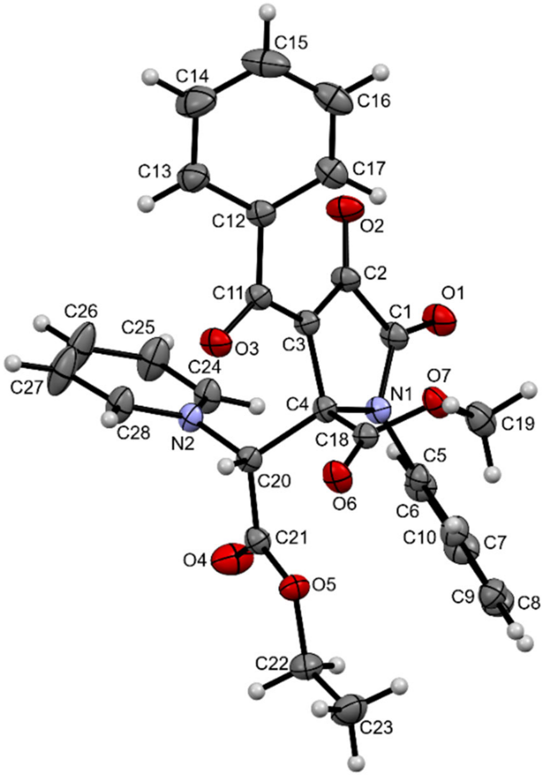

Figure S4. *Molecular structure of compound* ***3aa*** *showing 30% probability amplitude displacement ellipsoids (CCDC 2500563).*

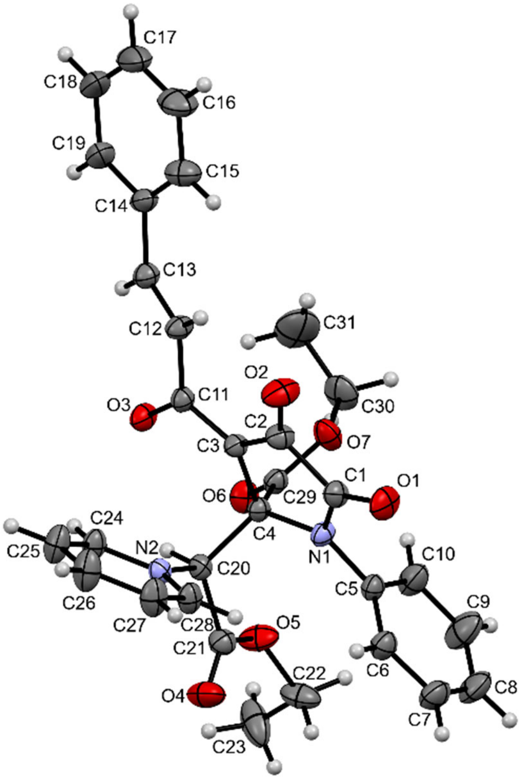

Figure S5. *Molecular structure of compound **3da** showing 30% probability amplitude displacement ellipsoids (CCDC 2500564).*

Table S3. Crystal data and structure refinement for compounds **3aa**, **3da**.

Compound	**3aa**	**3da**
CCDC	2500563	2500564
Empirical formula	$C_{28}H_{24}N_2O_7$	$C_{31}H_{28}N_2O_7$
Formula weight	500.49	540.55
Temperature, K	295(2)	295(2)
Crystal system	Monoclinic	Monoclinic
Space group	$P2_1/c$	$P2_1/n$
a, Å	12.963(3)	13.356(4)
b, Å	21.217(4)	15.057(4)
c, Å	9.2820(18)	15.344(5)
α, °	90	90
β, °	106.44(2)	112.55(4)
γ, °	90	90
Volume, Å^3	2448.4(9)	2849.8(16)

Z	4	4
Density (calculated), g/cm^3	1.358	1.260
Absorption coefficient, mm^{-1}	0.099	0.090
F(000)	1048.0	1136.0
Crystal size, mm^3	0.2 × 0.15 × 0.04	0.5 × 0.05 × 0.04
Radiation	Mo Kα (λ = 0.71073)	Mo Kα (λ = 0.71073)
2Θ range for data collection, °	4.962 to 59.032	3.946 to 58.752
Index ranges	-17 ≤ h ≤ 16, -26 ≤ k ≤ 20, -11 ≤ l ≤ 12	-17 ≤ h ≤ 18, -20 ≤ k ≤ 18, -19 ≤ l ≤ 17
Reflections collected	12800	15366
Independent reflections	5761 [R_{int} = 0.0519, R_{sigma} = 0.0754]	6710 [R_{int} = 0.0524, R_{sigma} = 0.0875]
Data/restraints/parameters	5761/0/336	6710/3/384
Goodness-of-fit on F^2	1.008	1.023
Final R indexes [I>=2σ (I)]	R_1 = 0.0593, wR_2 = 0.1285	R_1 = 0.0778, wR_2 = 0.1873
Final R indexes [all data]	R_1 = 0.1074, wR_2 = 0.1562	R_1 = 0.1499, wR_2 = 0.2398
Largest diff. peak/hole, eÅ^{-3}	0.21/-0.27	0.89/-0.23

Copies of NMR spectra

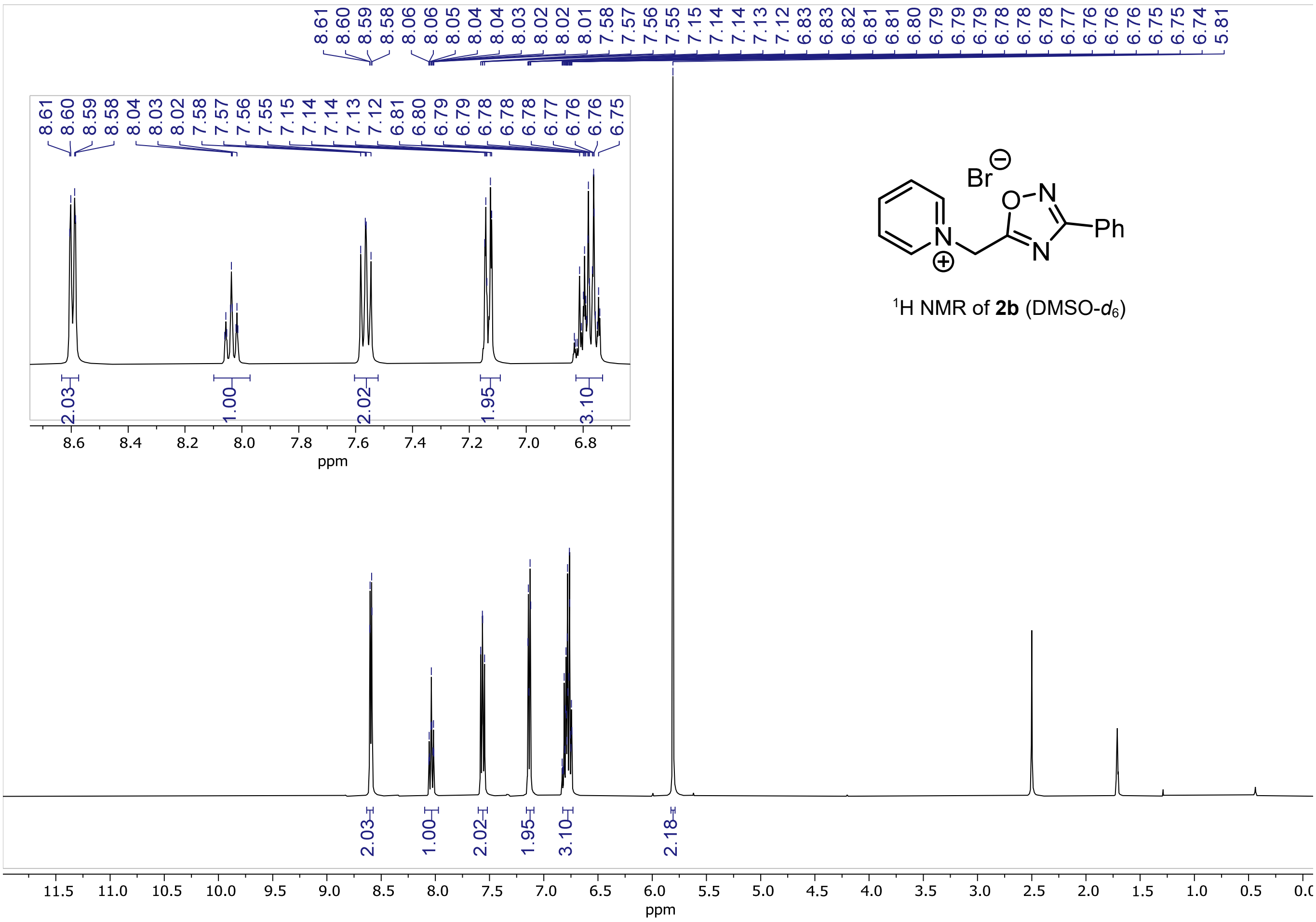

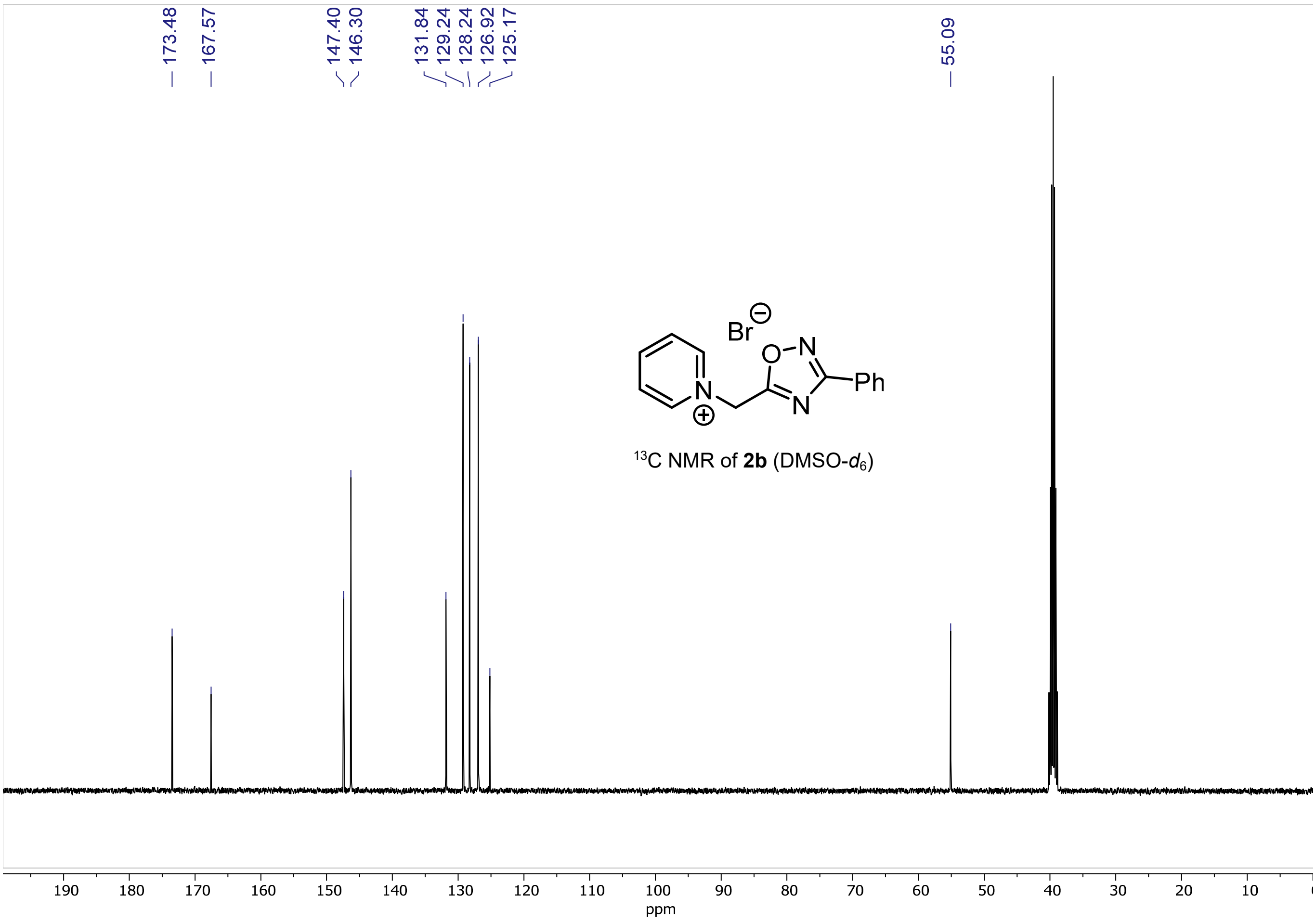

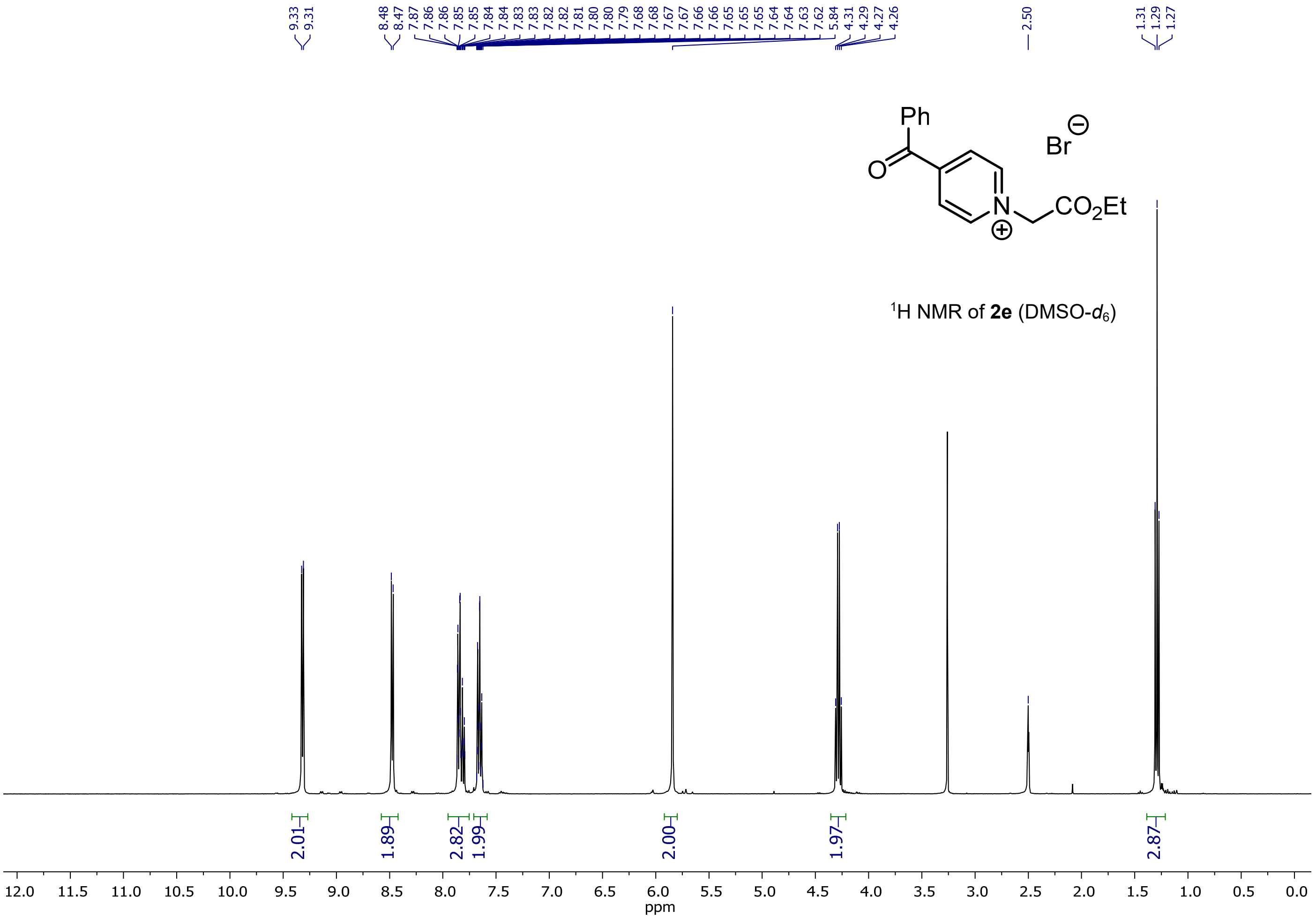
Ph
Br
N
CO2Et
O
1H NMR of 2e (DMSO-d6)
2.01
1.89
2.82
1.99
2.00
1.97
2.87
ppm

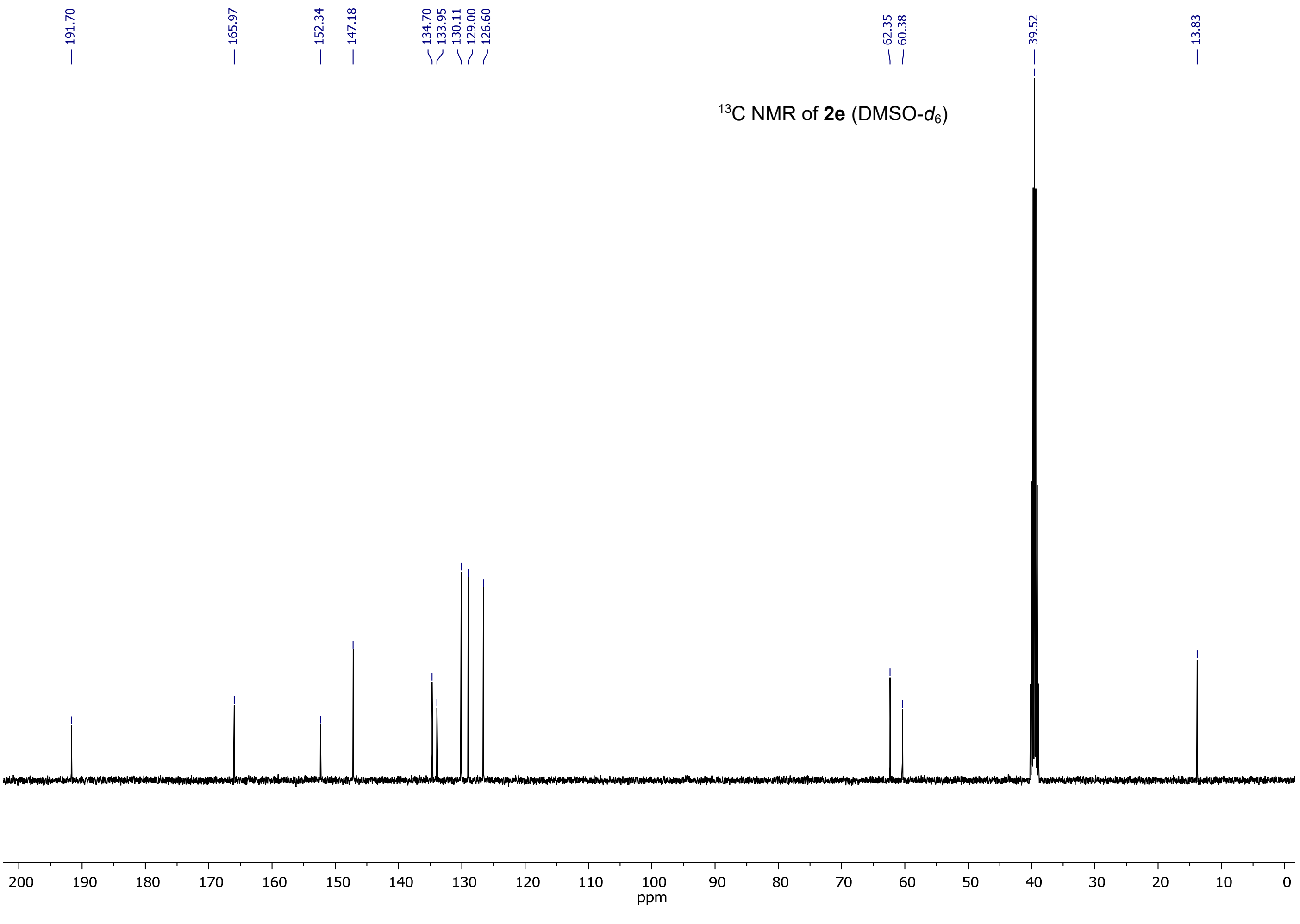
191.70
165.97
152.34
147.18
134.70
133.95
130.11
129.00
126.60
62.35
60.38
39.52
13.83
13C NMR of 2e (DMSO-d6)
ppm

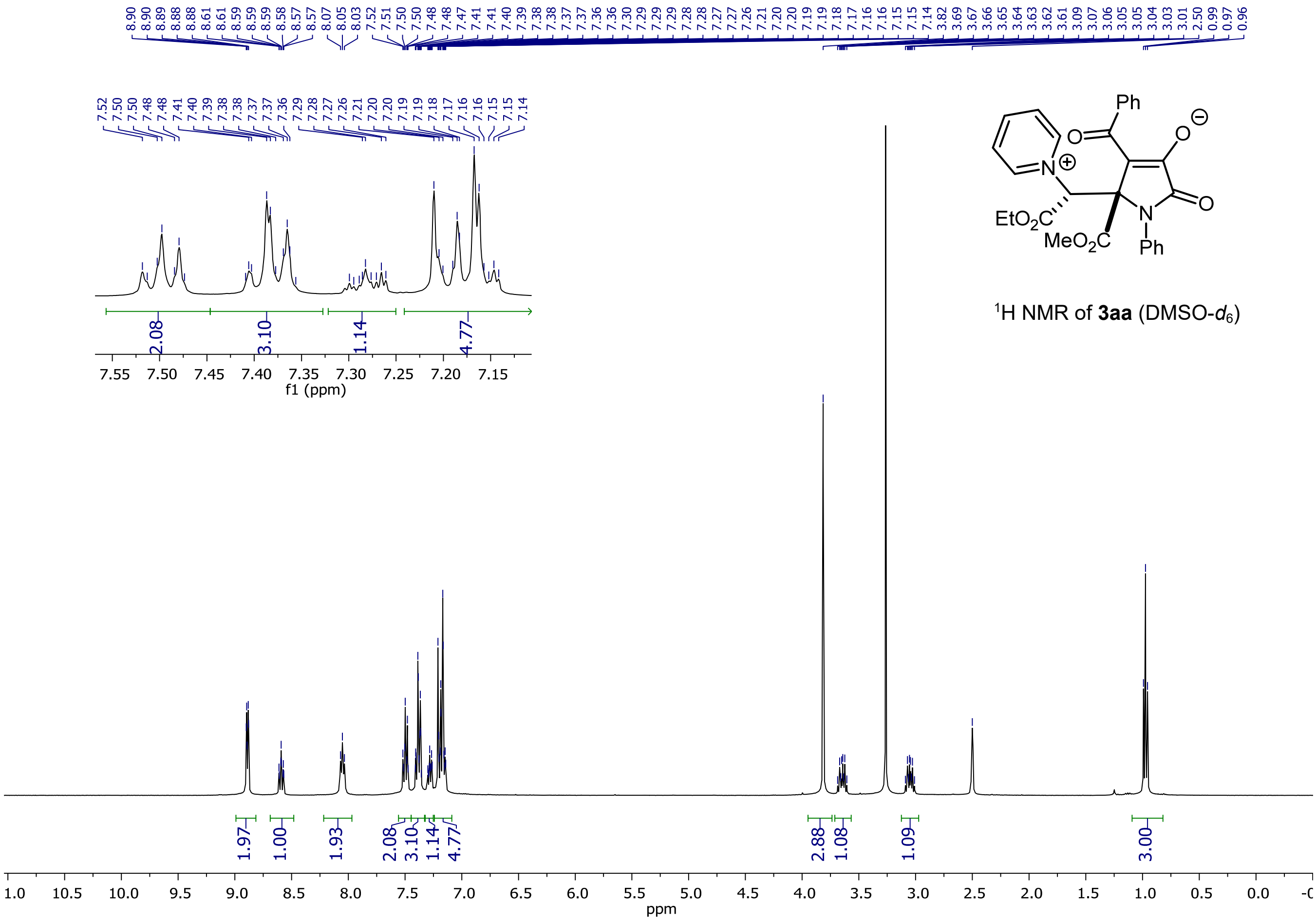

^{1}H NMR of **3aa** (DMSO-d_6)

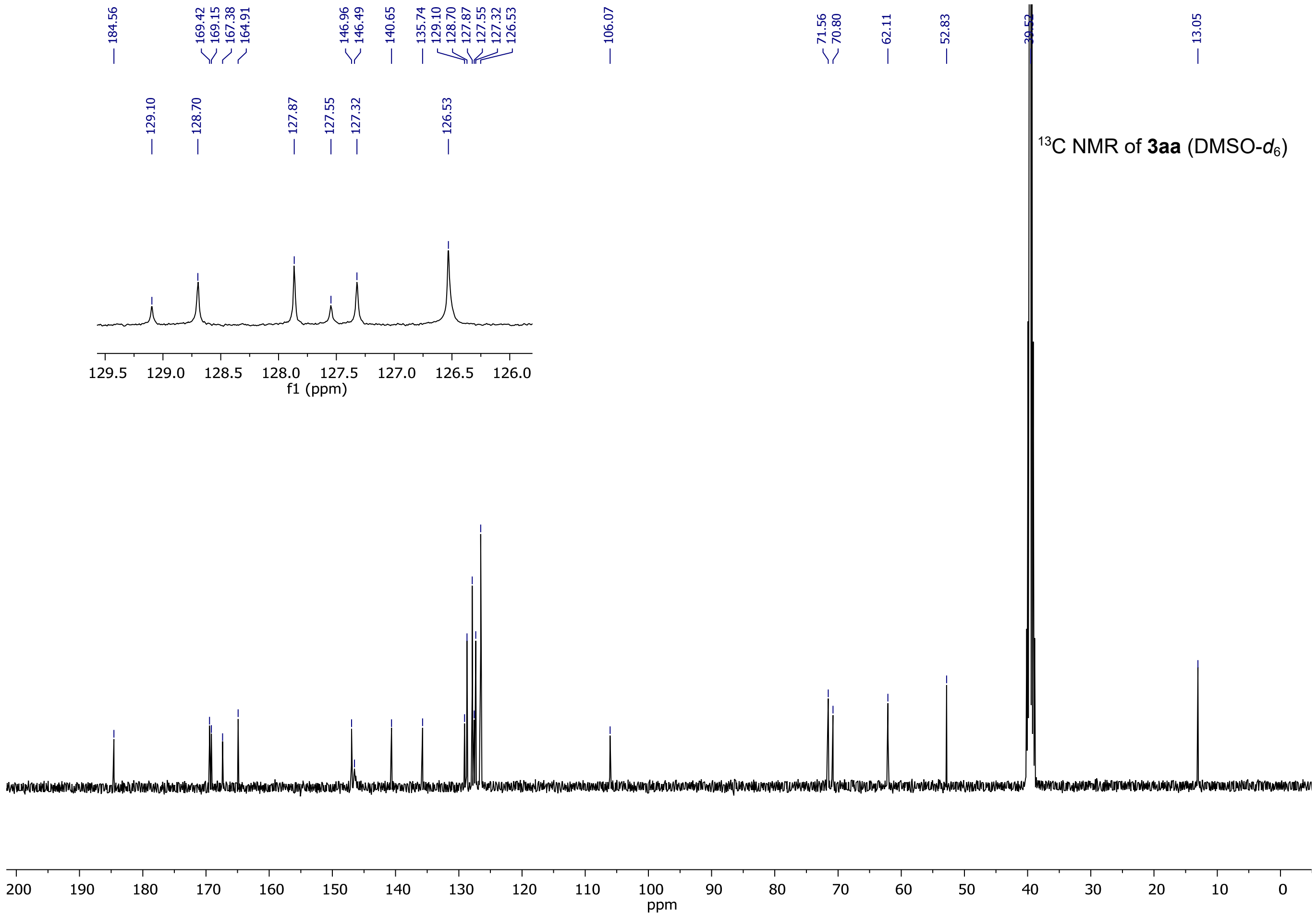

^{13}C NMR of **3aa** (DMSO-d_6)

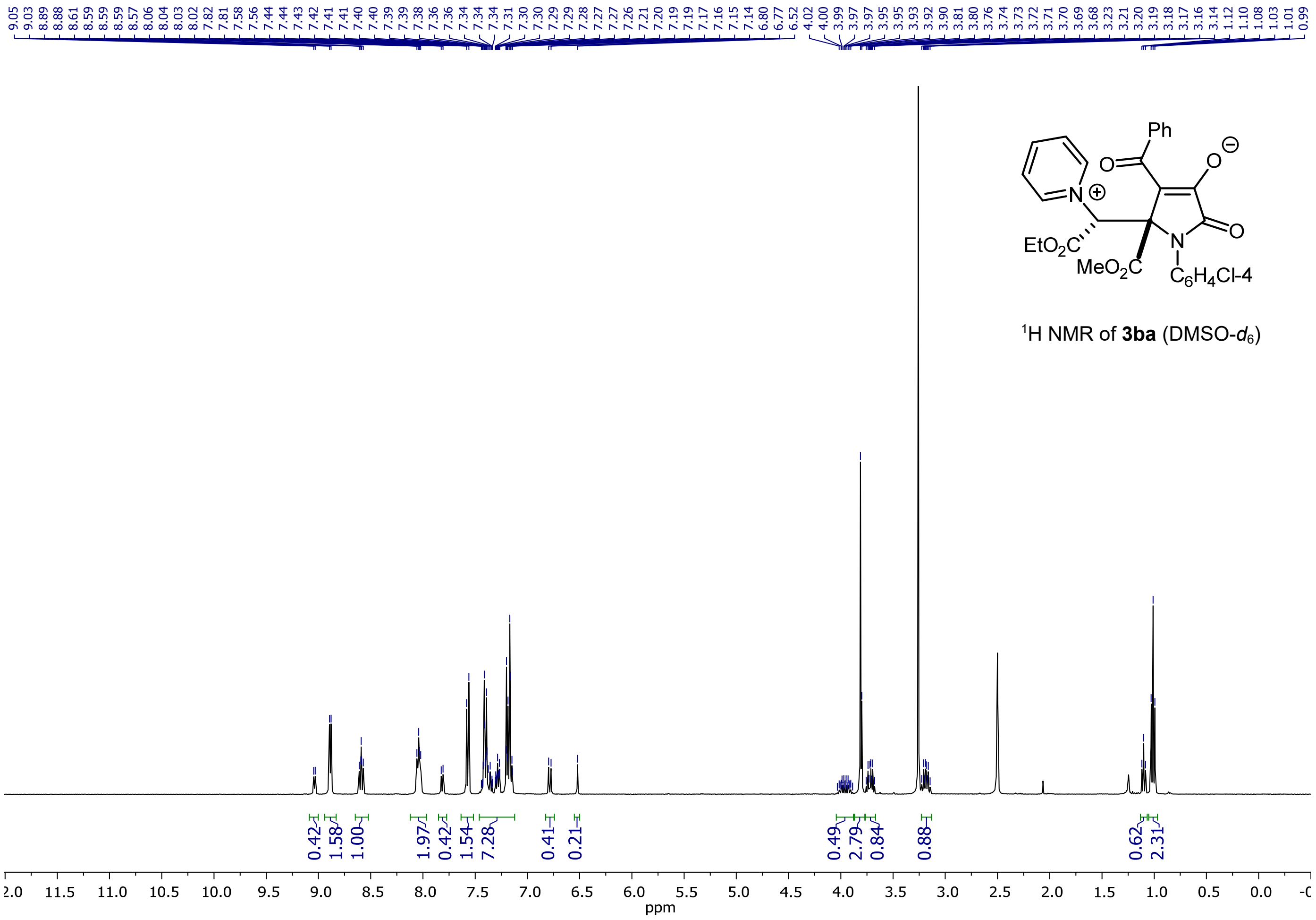
Ph
O
N
EtO2C
MeO2C
C6H4Cl-4
1H NMR of 3ba (DMSO-d6)
ppm

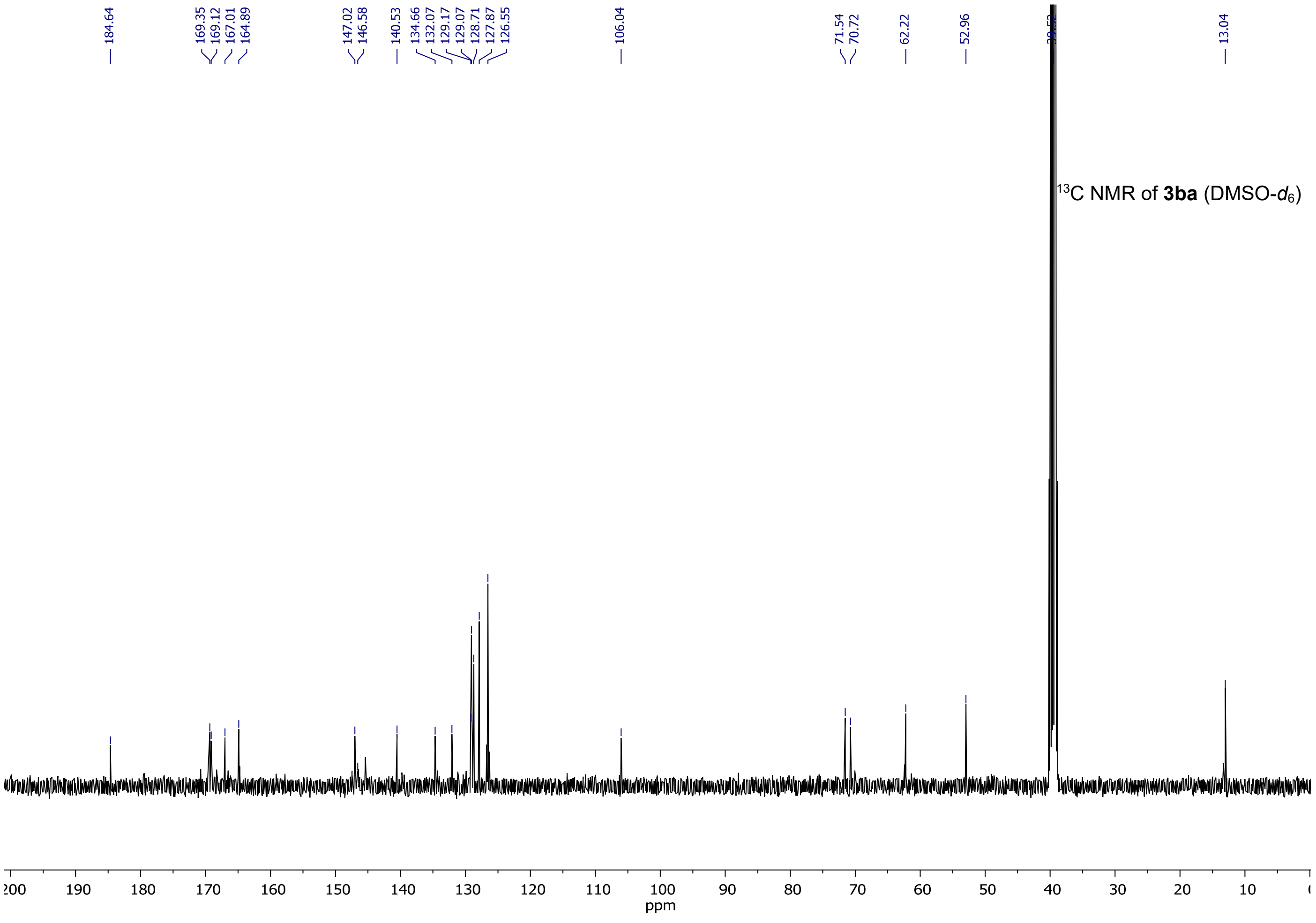
13C NMR of 3ba (DMSO-d6)
ppm

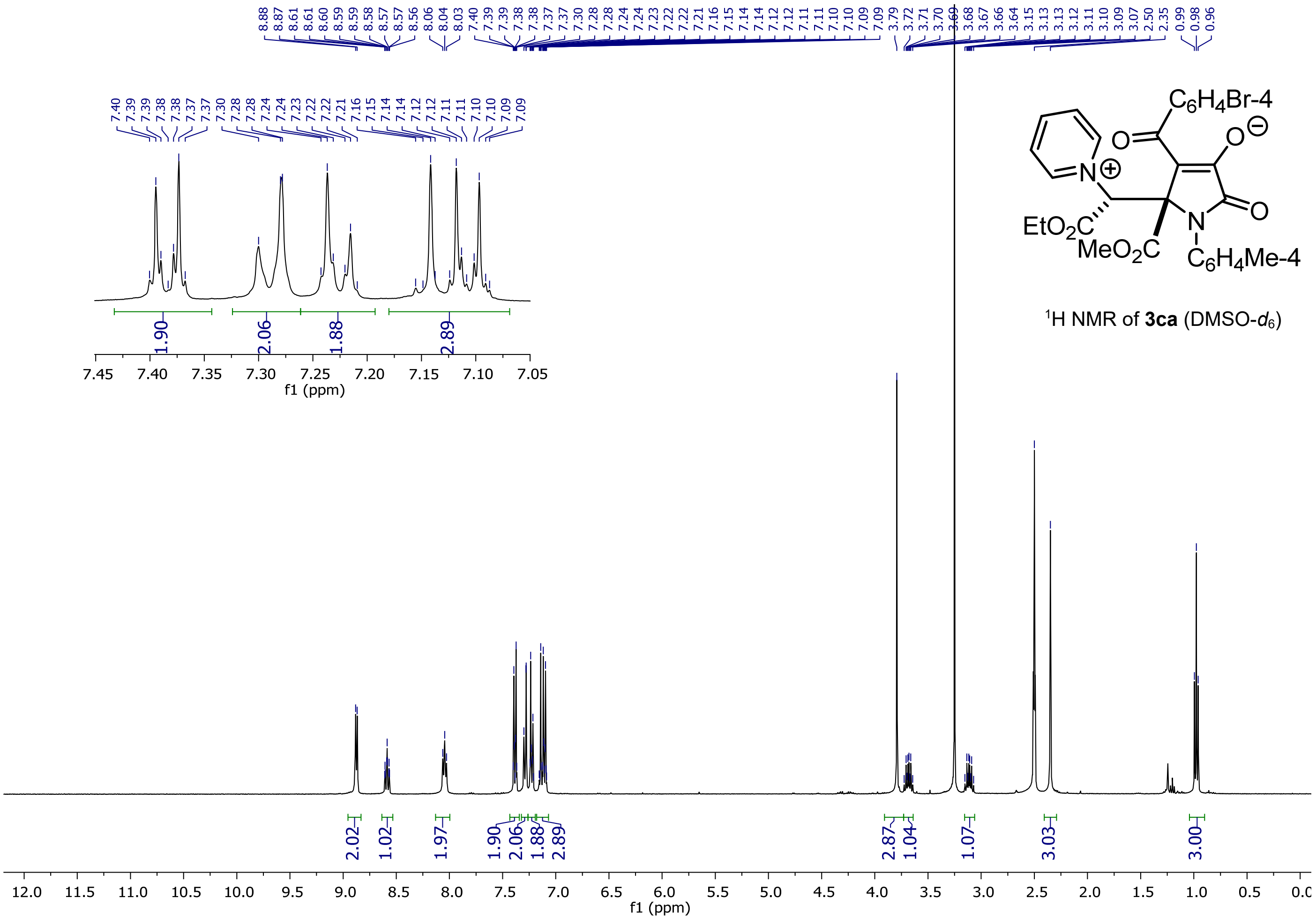

^{1}H NMR of **3ca** (DMSO-d_6)

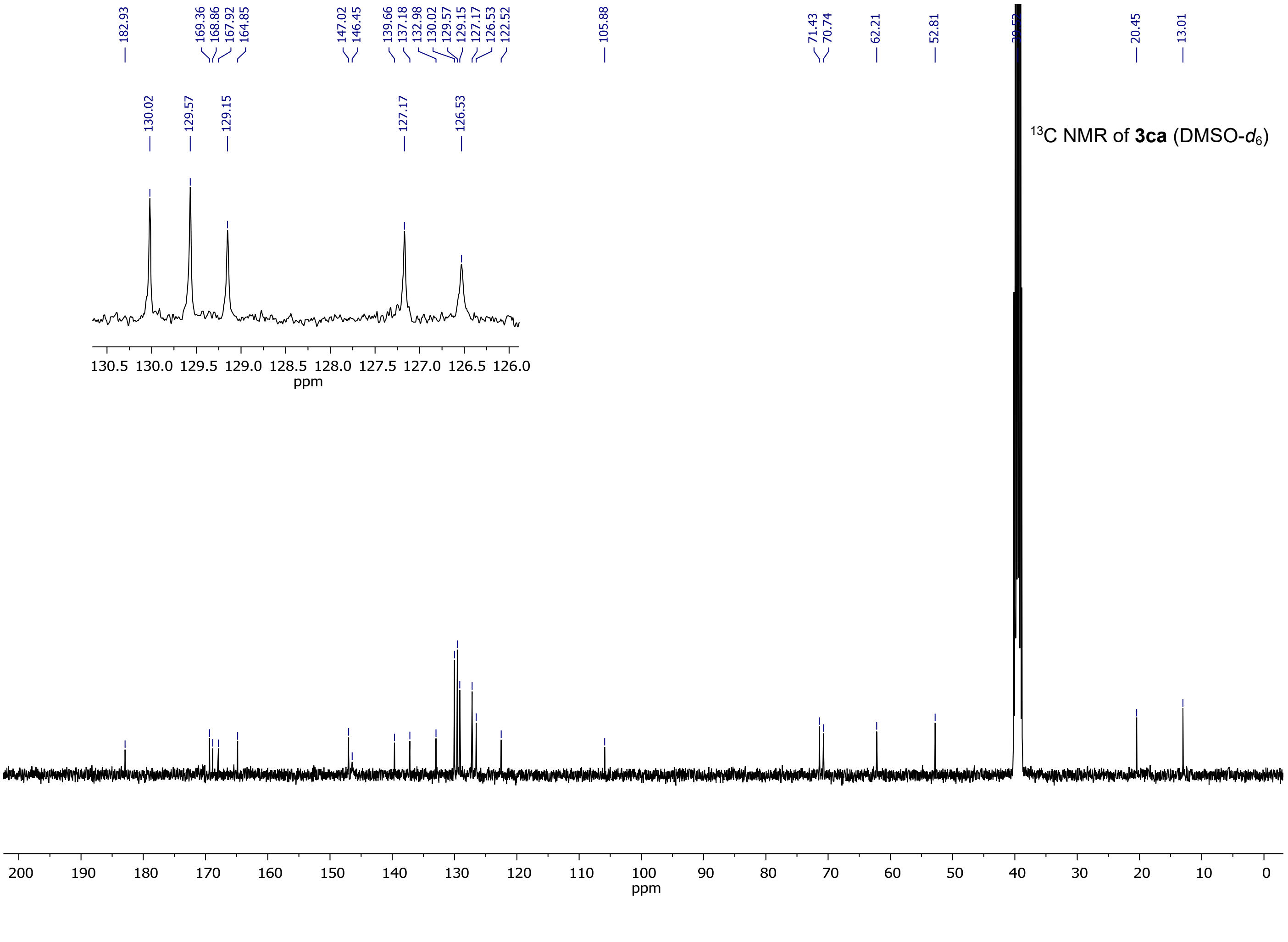

^{13}C NMR of **3ca** (DMSO-d_6)

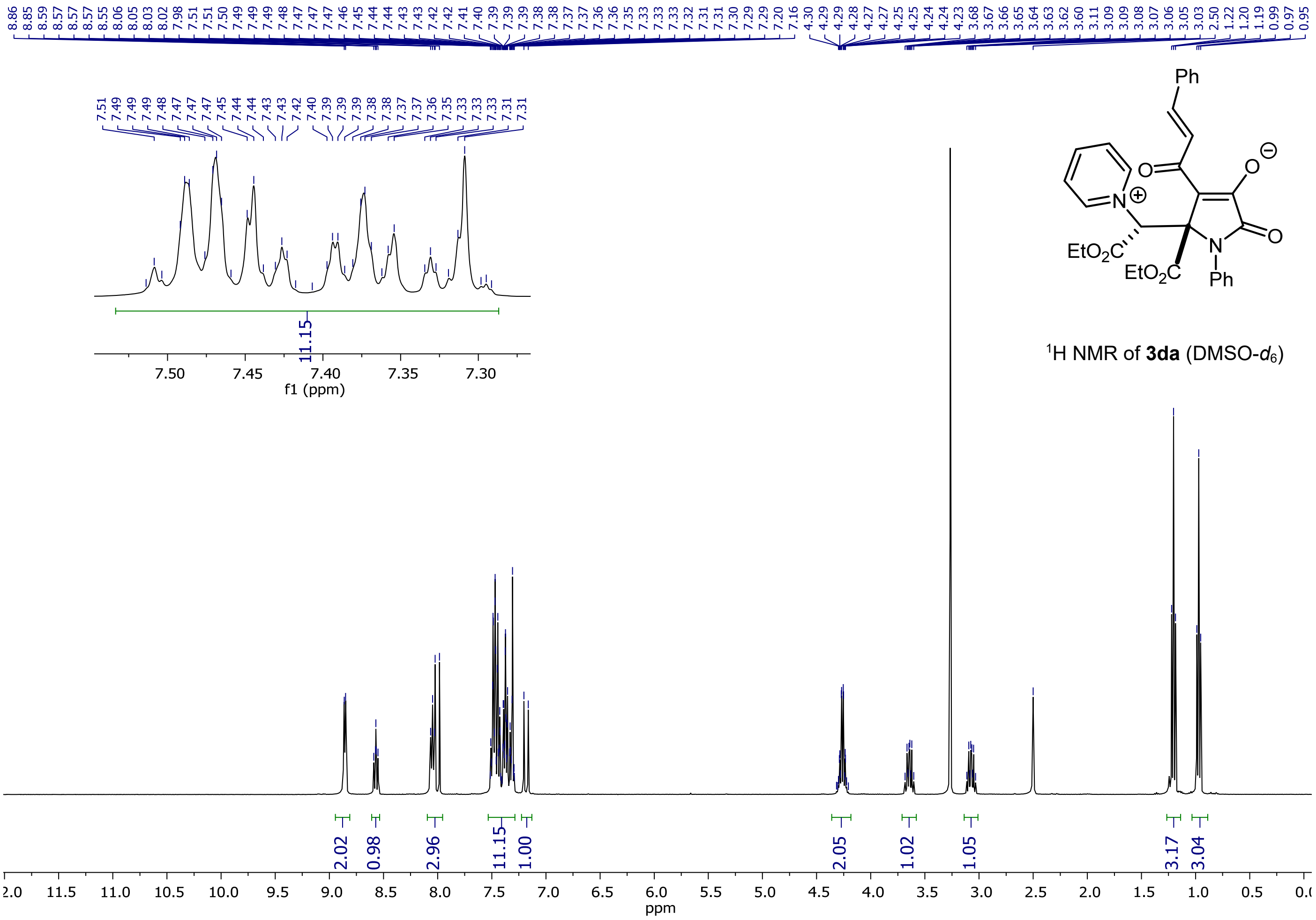

^{1}H NMR of **3da** (DMSO-d_6)

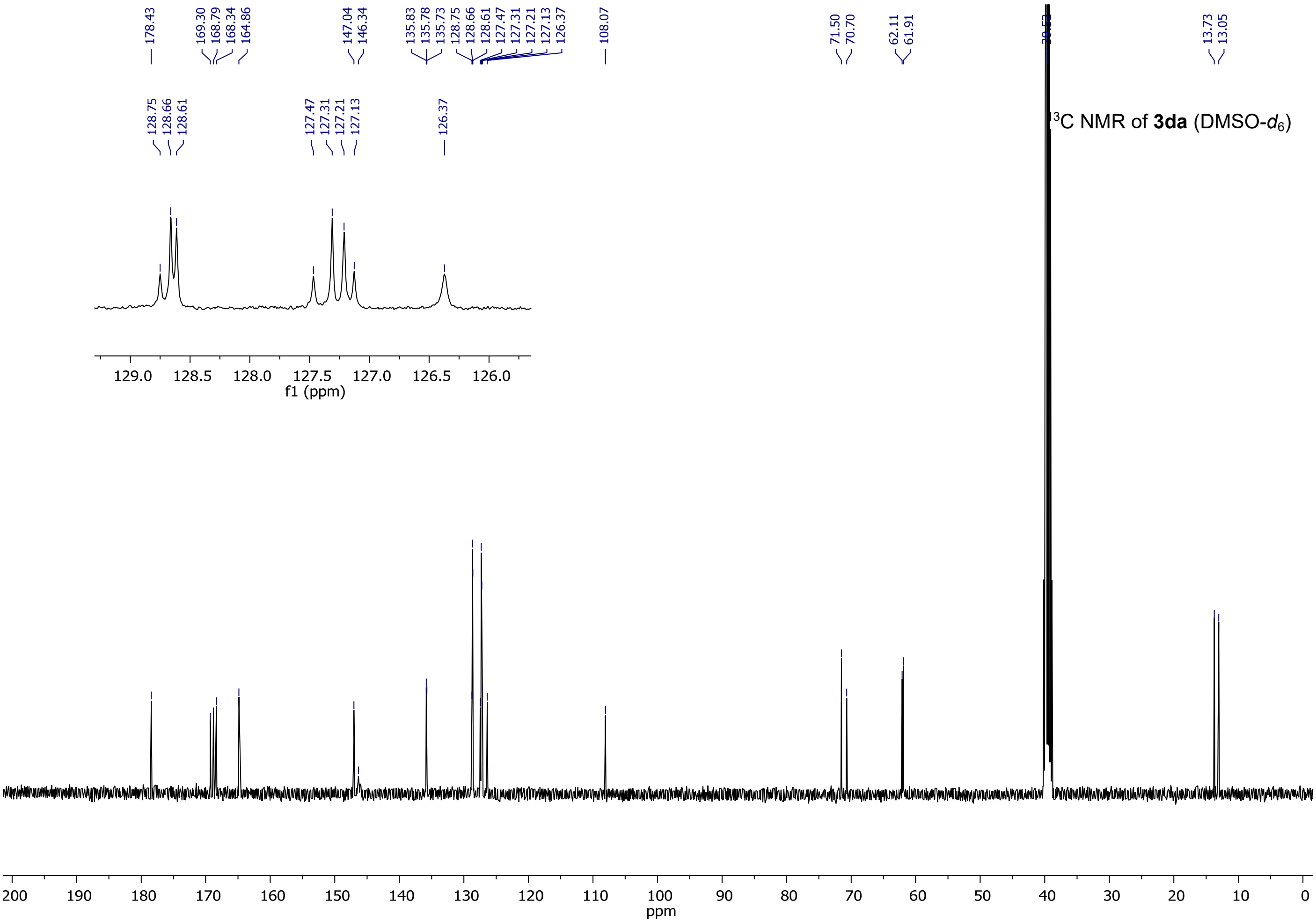

^{13}C NMR of **3da** (DMSO-d_6)

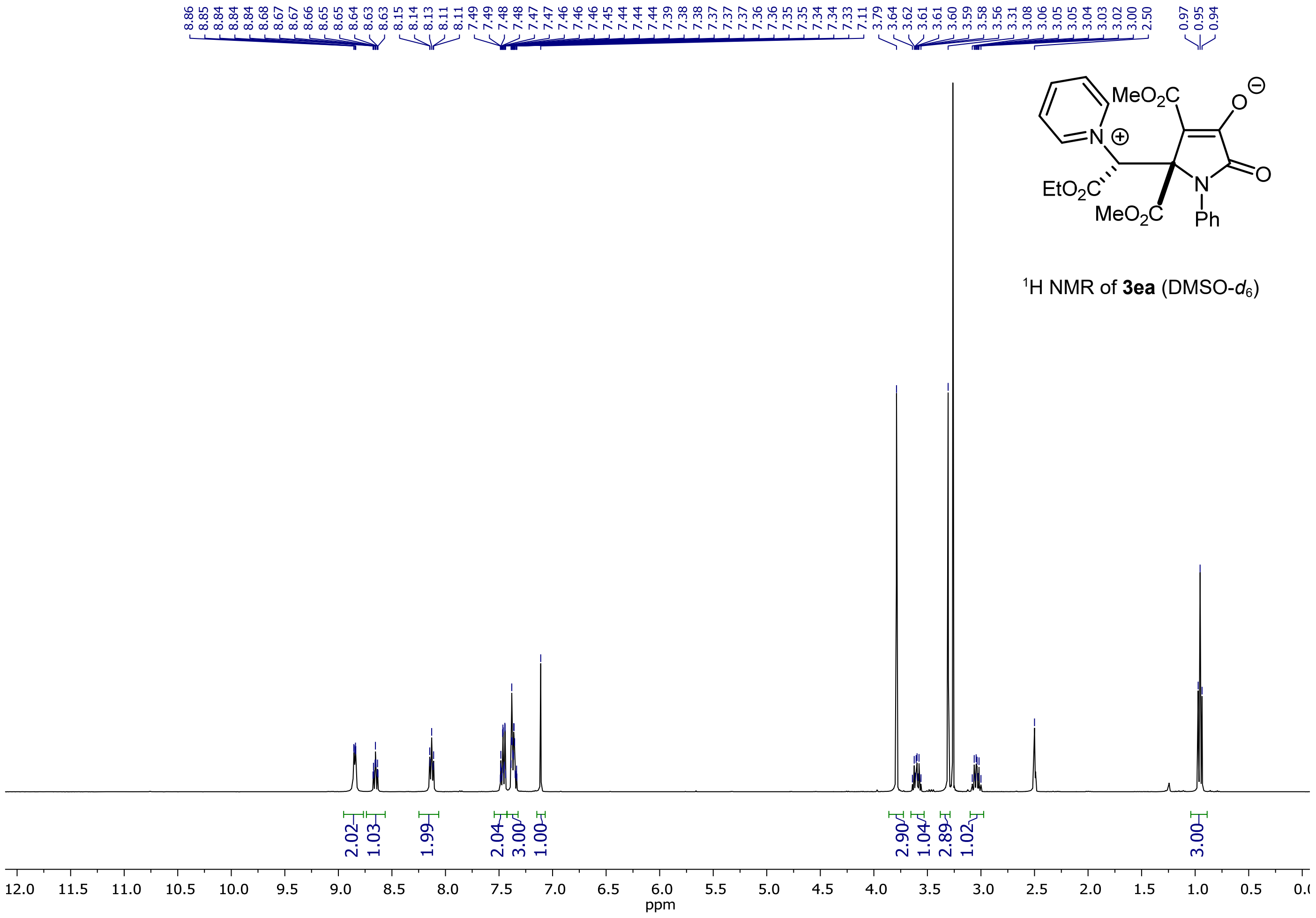
^{1}H NMR of **3ea** (DMSO-d_6)
ppm

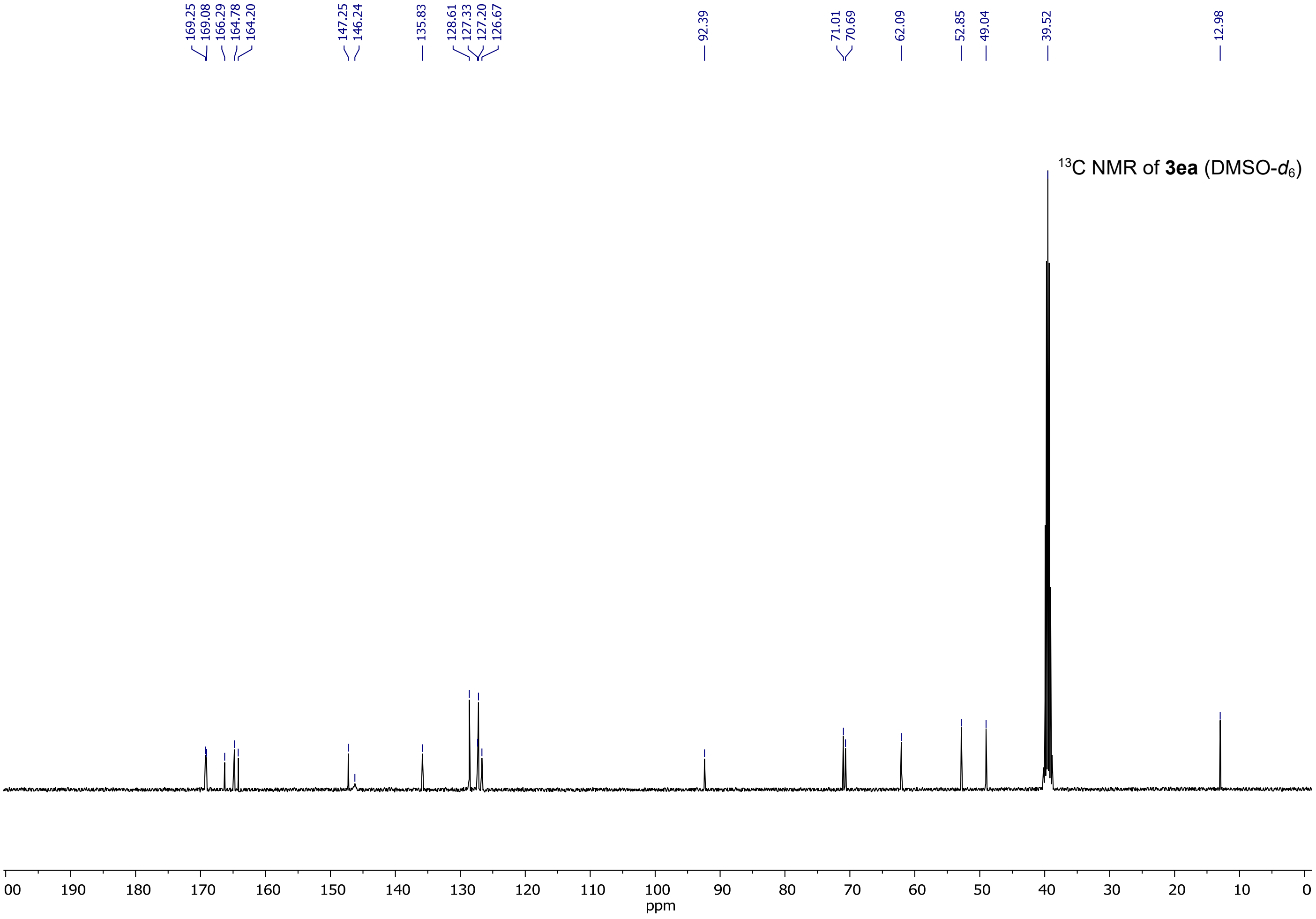
^{13}C NMR of **3ea** (DMSO-d_6)
ppm

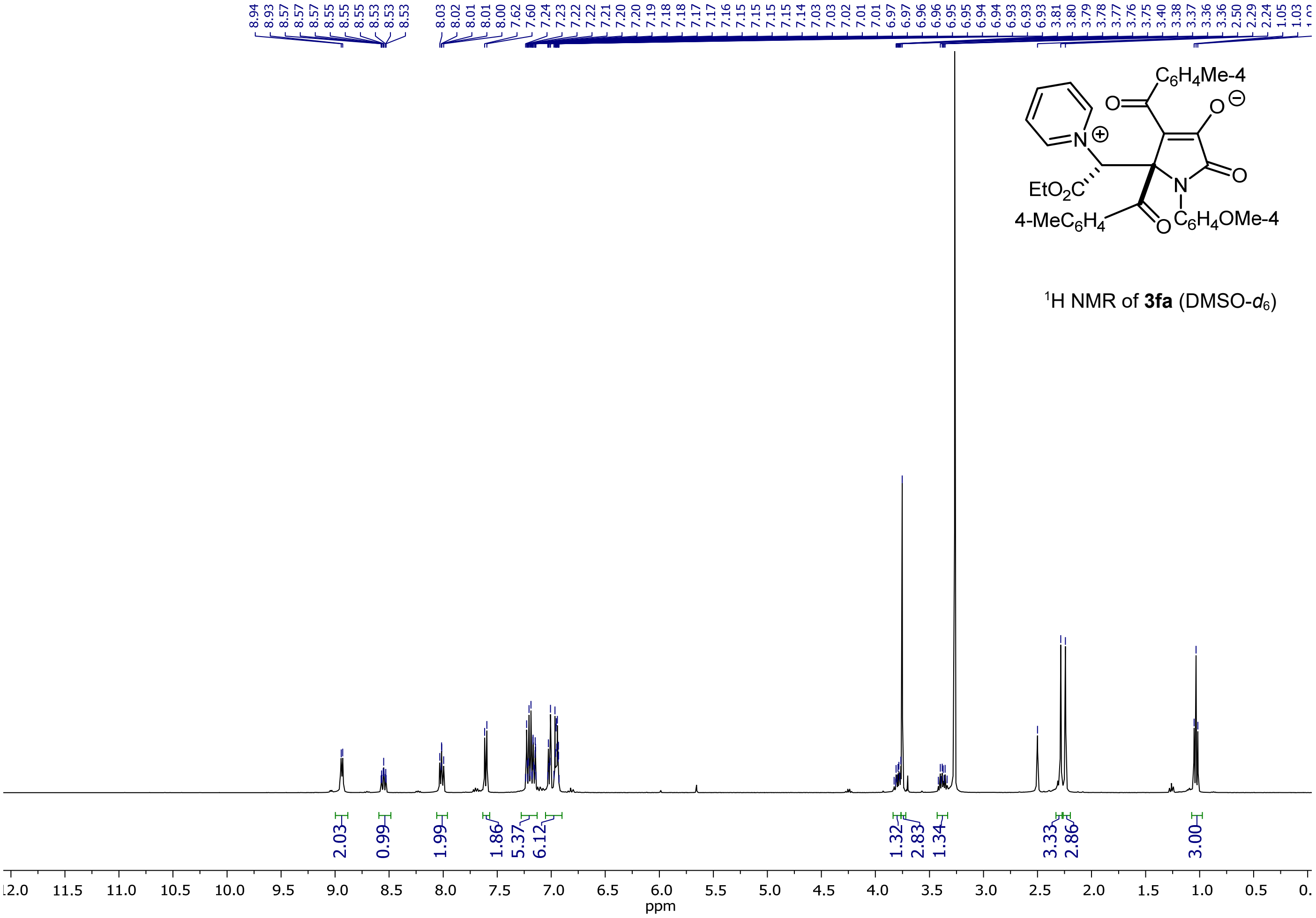

^{1}H NMR of **3fa** (DMSO-d_6)

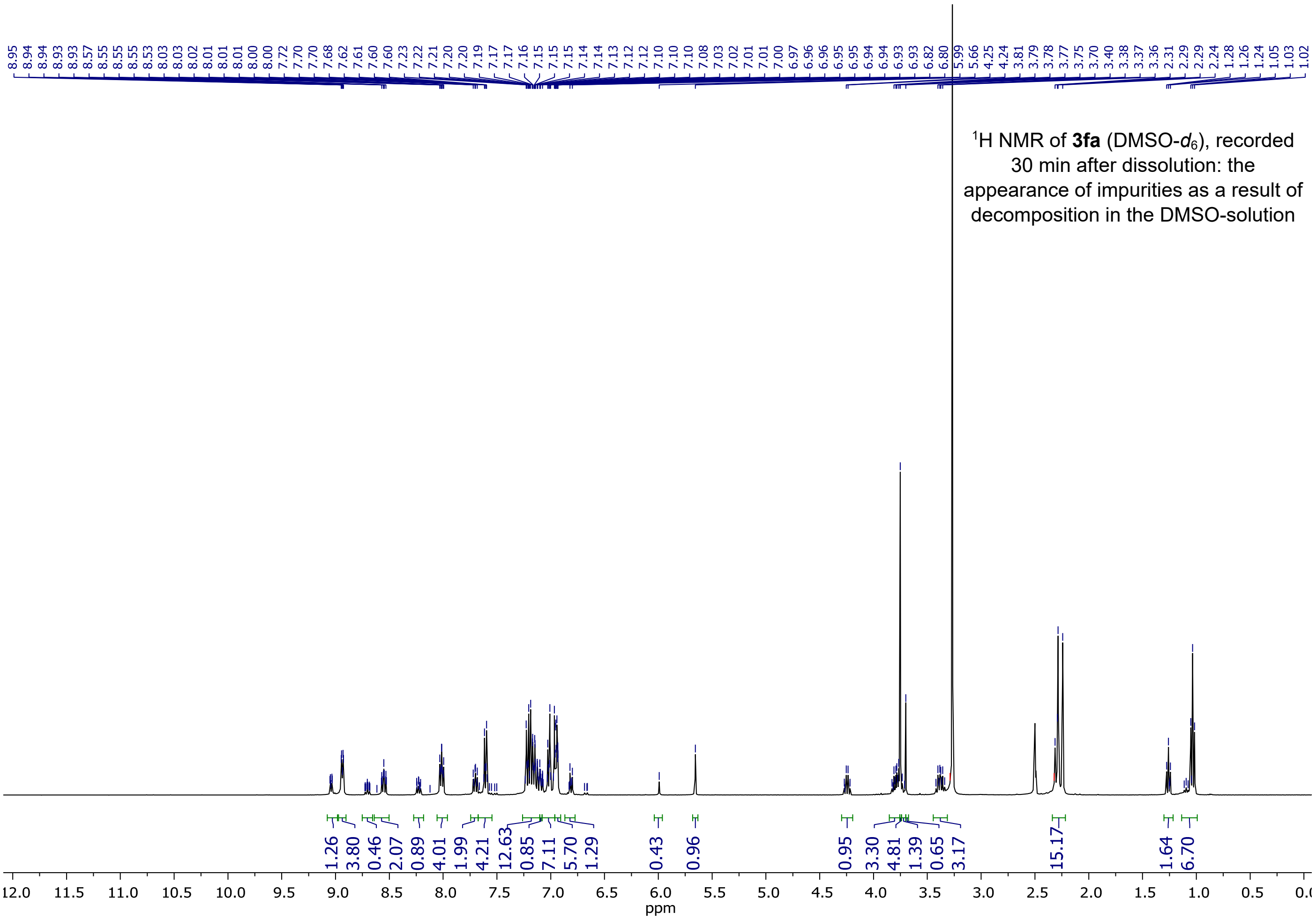

^{1}H NMR of **3fa** (DMSO-d_6), recorded 30 min after dissolution: the appearance of impurities as a result of decomposition in the DMSO-solution

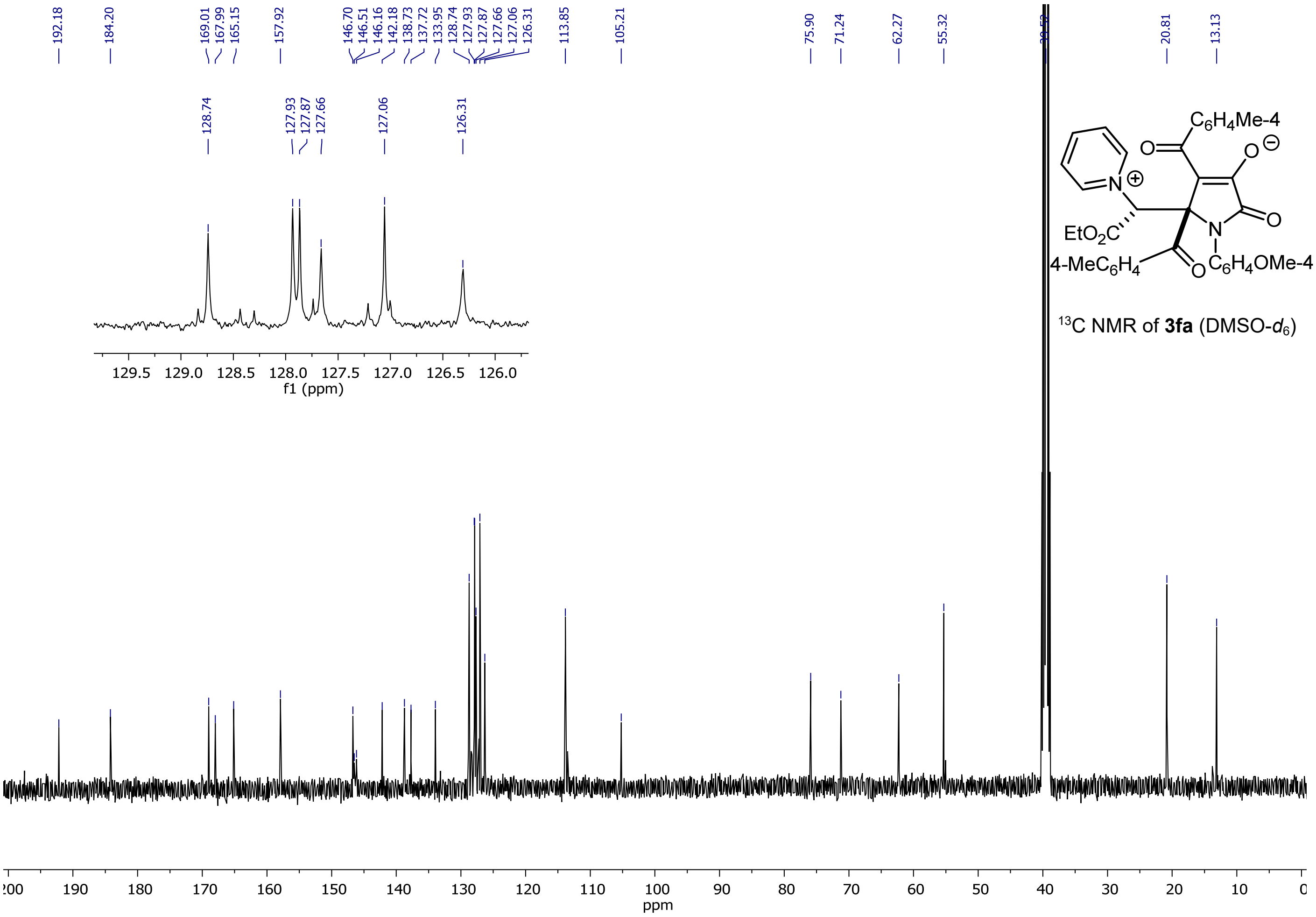

^{13}C NMR of **3fa** (DMSO-d_6)

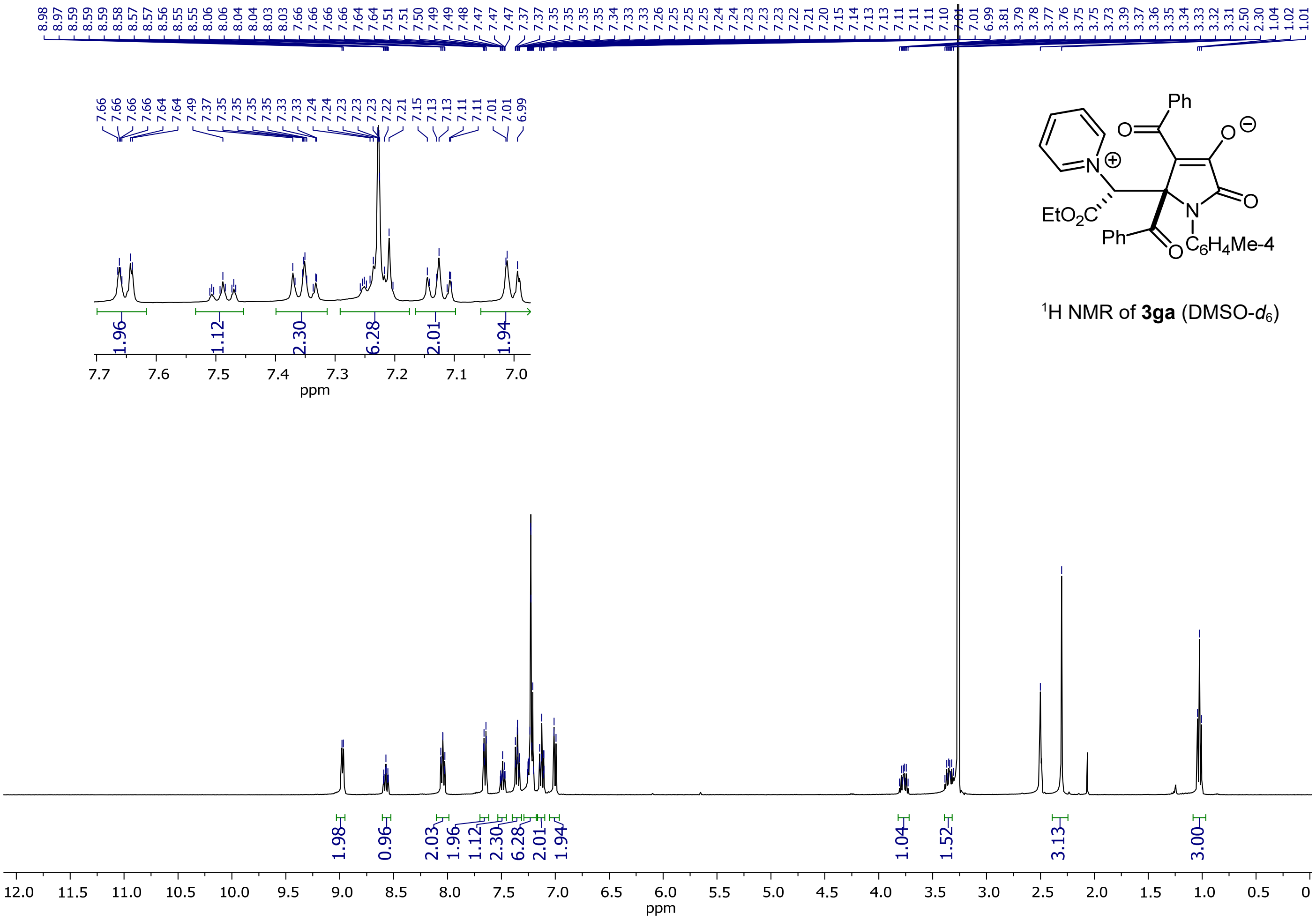

^{1}H NMR of **3ga** (DMSO-d_6)

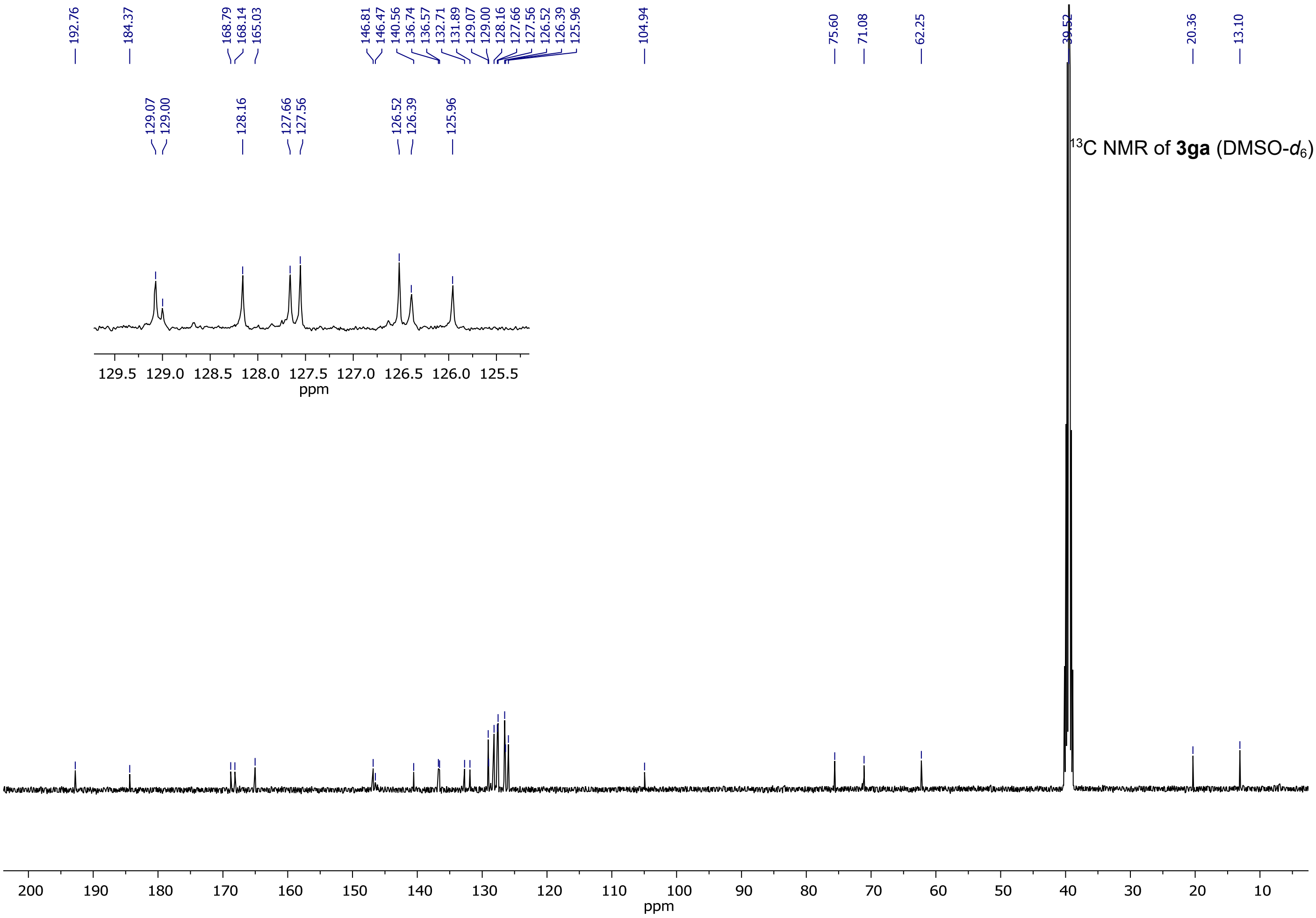

^{13}C NMR of **3ga** (DMSO-d_6)

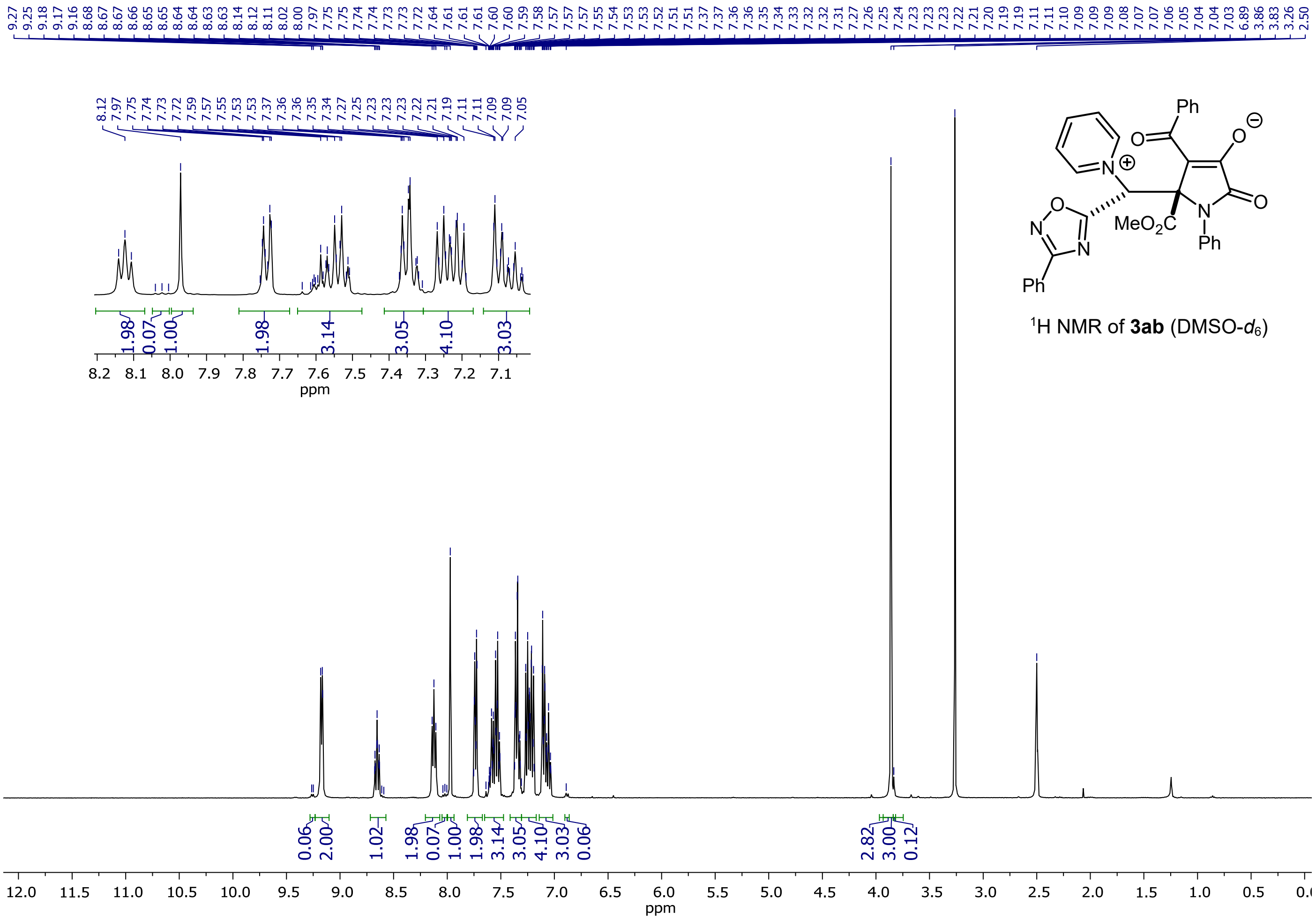
9.27 9.25 9.18 9.17 9.16 8.68 8.67 8.67 8.66 8.65 8.65 8.64 8.64 8.63 8.63 8.14 8.12 8.11 8.02 8.00 7.97 7.75 7.75 7.74 7.73 7.73 7.72 7.64 7.61 7.61 7.60 7.60 7.59 7.58 7.57 7.57 7.57 7.55 7.54 7.53 7.53 7.52 7.51 7.51 7.37 7.37 7.36 7.36 7.35 7.34 7.33 7.32 7.32 7.31 7.27 7.26 7.25 7.24 7.23 7.23 7.23 7.22 7.22 7.21 7.20 7.19 7.19 7.11 7.11 7.10 7.09 7.09 7.09 7.08 7.07 7.07 7.06 7.05 7.04 7.04 7.03 6.89 3.86 3.83 3.26 2.50
8.12 7.97 7.75 7.74 7.73 7.72 7.59 7.57 7.55 7.53 7.53 7.37 7.36 7.36 7.35 7.34 7.27 7.25 7.23 7.23 7.23 7.22 7.21 7.19 7.11 7.11 7.09 7.09 7.05
1.98 0.07 1.00 1.98 3.14 3.05 4.10 3.03
ppm
Ph
O
N
MeO2C
Ph
1H NMR of 3ab (DMSO-d6)
0.06 2.00 1.02 1.98 0.07 1.00 1.98 3.14 3.05 4.10 3.03 0.06 2.82 3.00 0.12
ppm

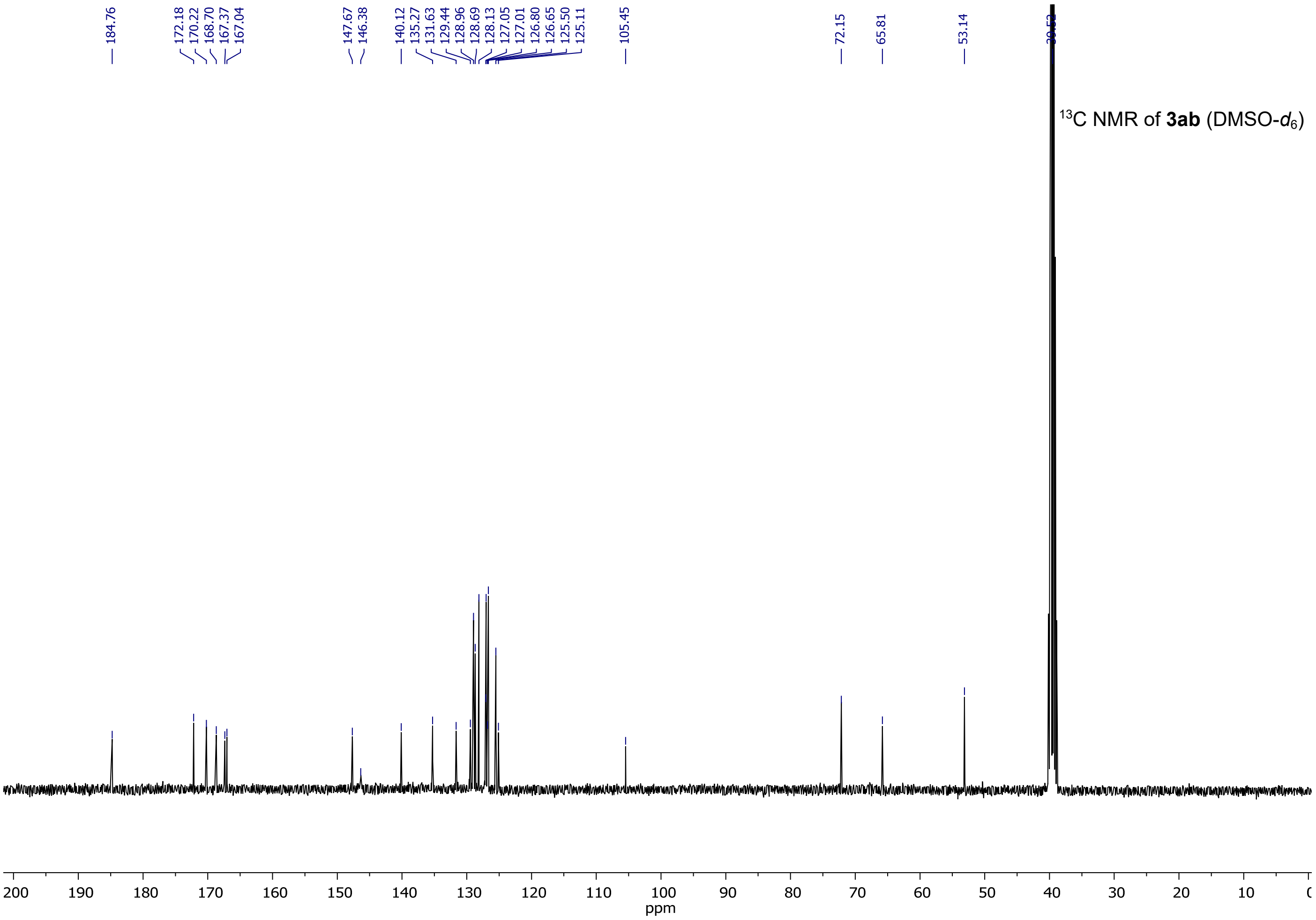
184.76 172.18 170.22 168.70 167.37 167.04 147.67 146.38 140.12 135.27 131.63 129.44 128.96 128.69 128.13 127.05 127.01 126.80 126.65 125.50 125.11 105.45 72.15 65.81 53.14
13C NMR of 3ab (DMSO-d6)
ppm

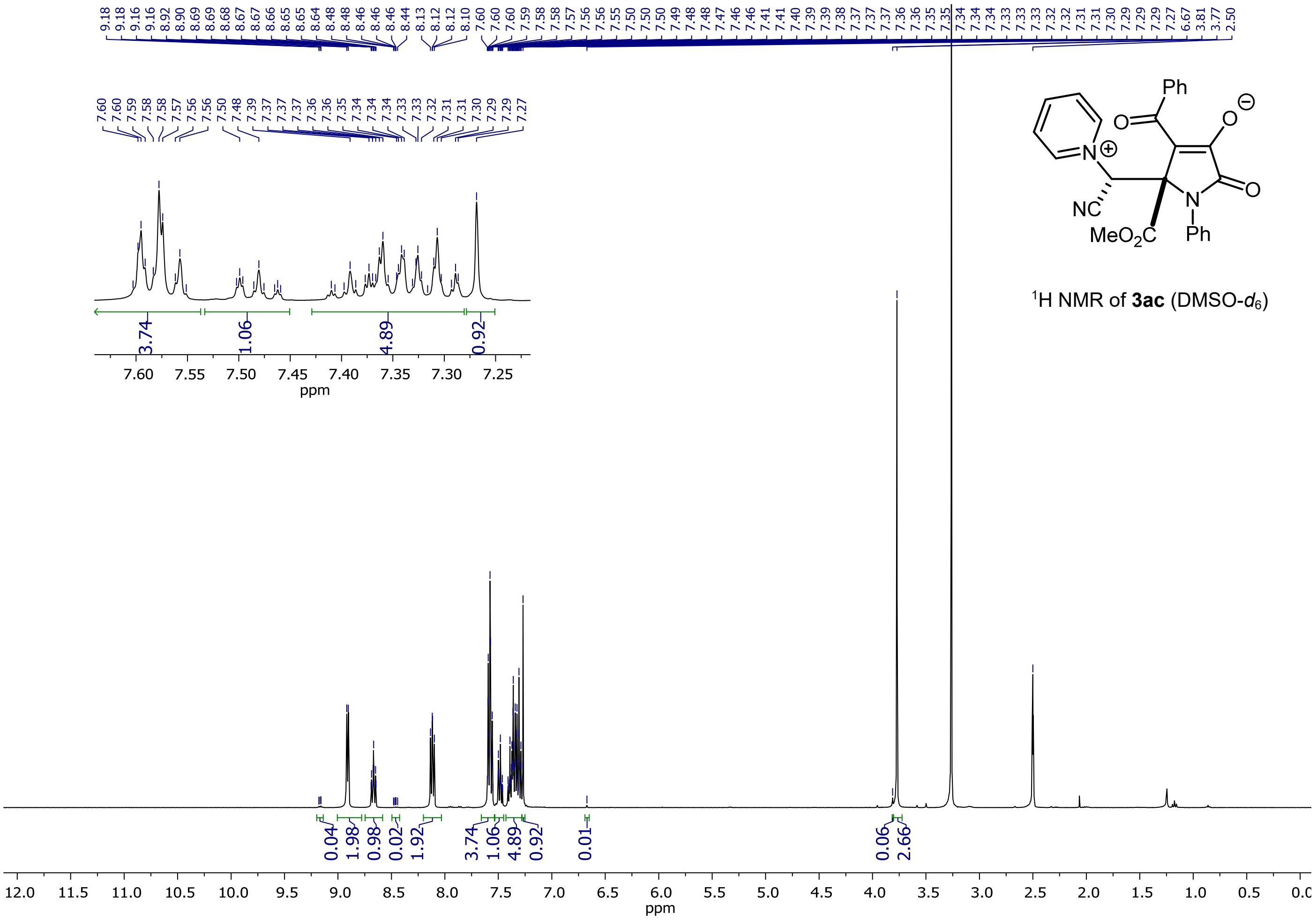

^{1}H NMR of **3ac** (DMSO-d_6)

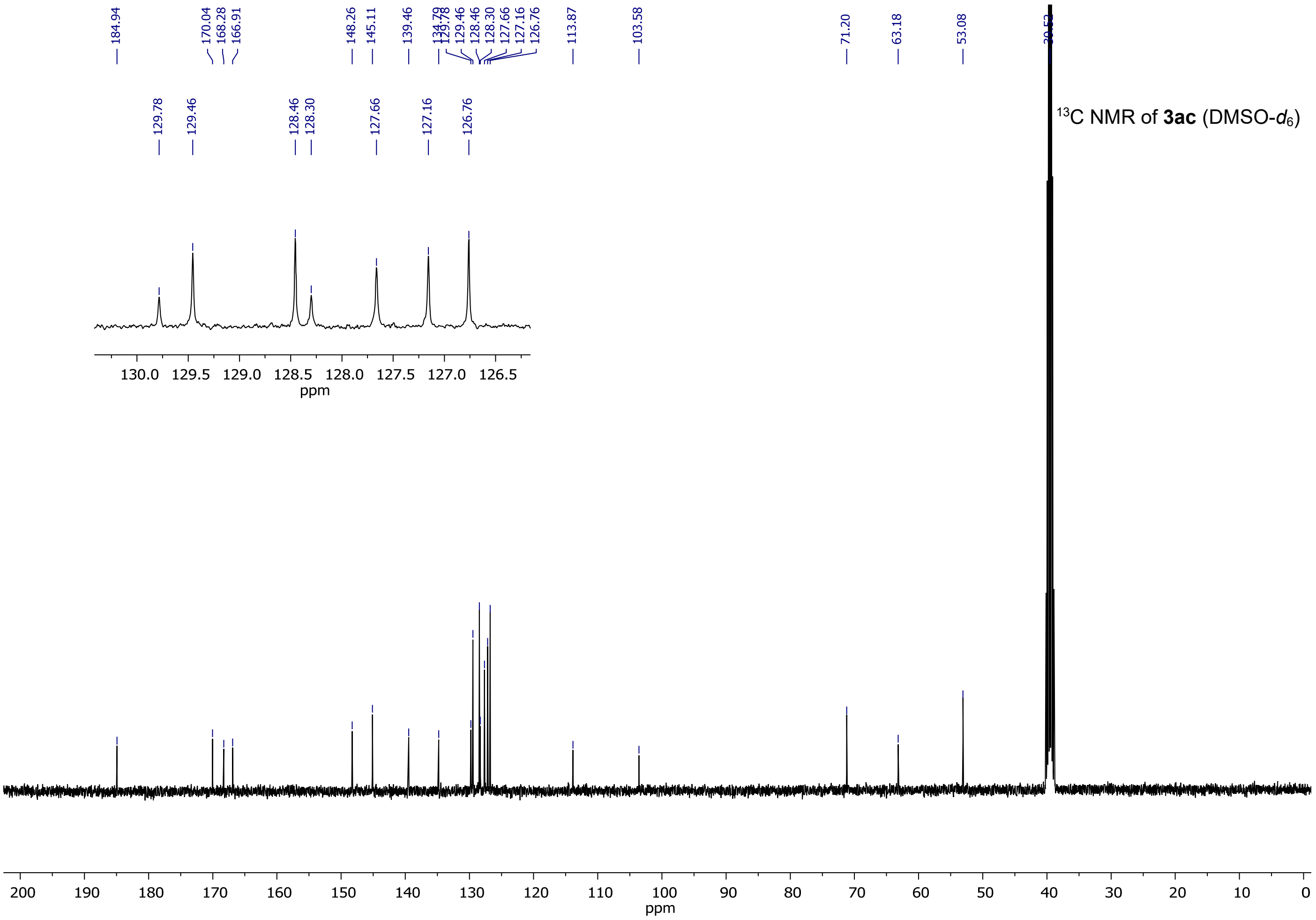

^{13}C NMR of **3ac** (DMSO-d_6)

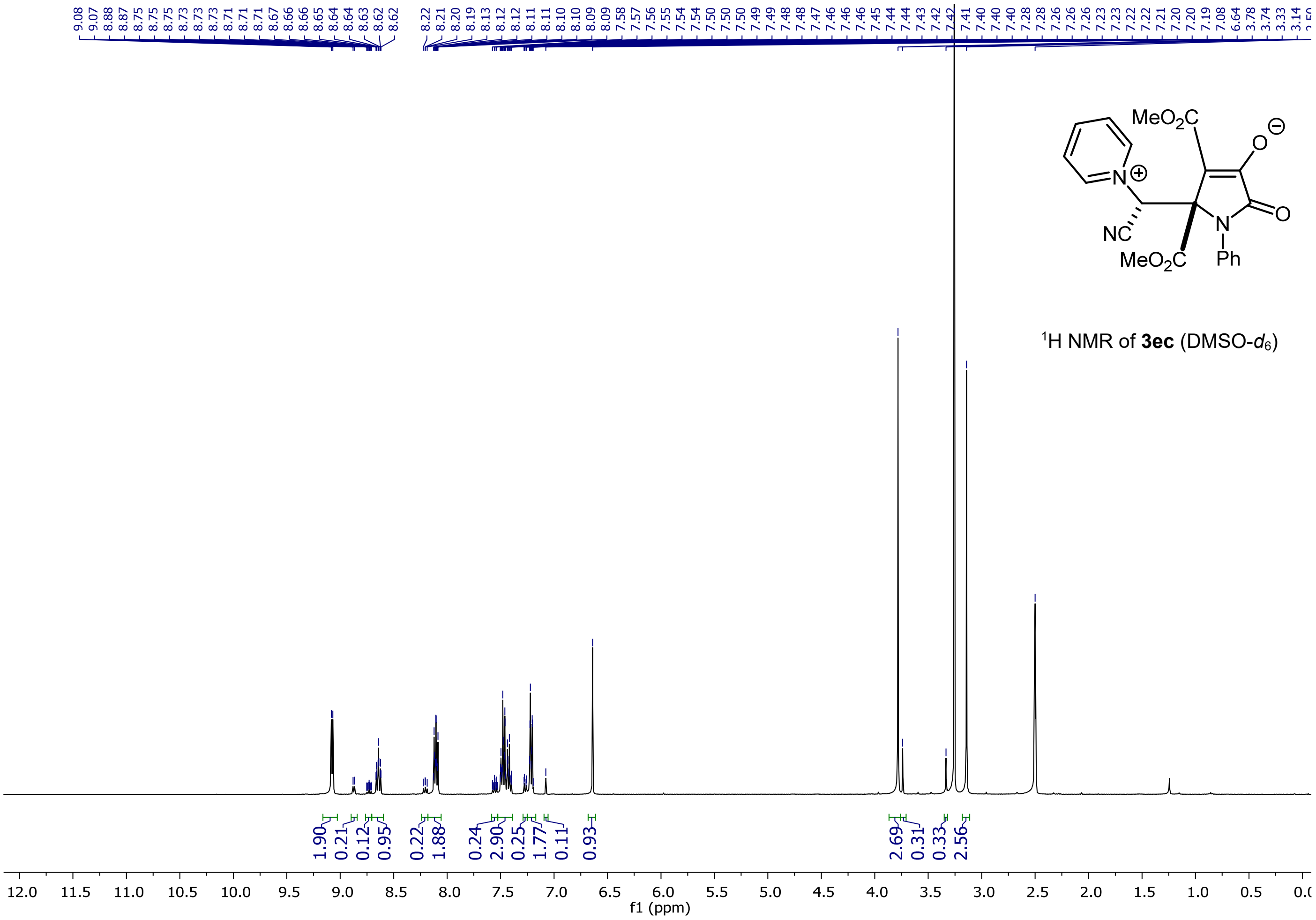
MeO$_2$C
O$^{\ominus}$
N$^{\oplus}$
NC
N
O
MeO$_2$C
Ph
^{1}H NMR of **3ec** (DMSO-d_6)
f1 (ppm)

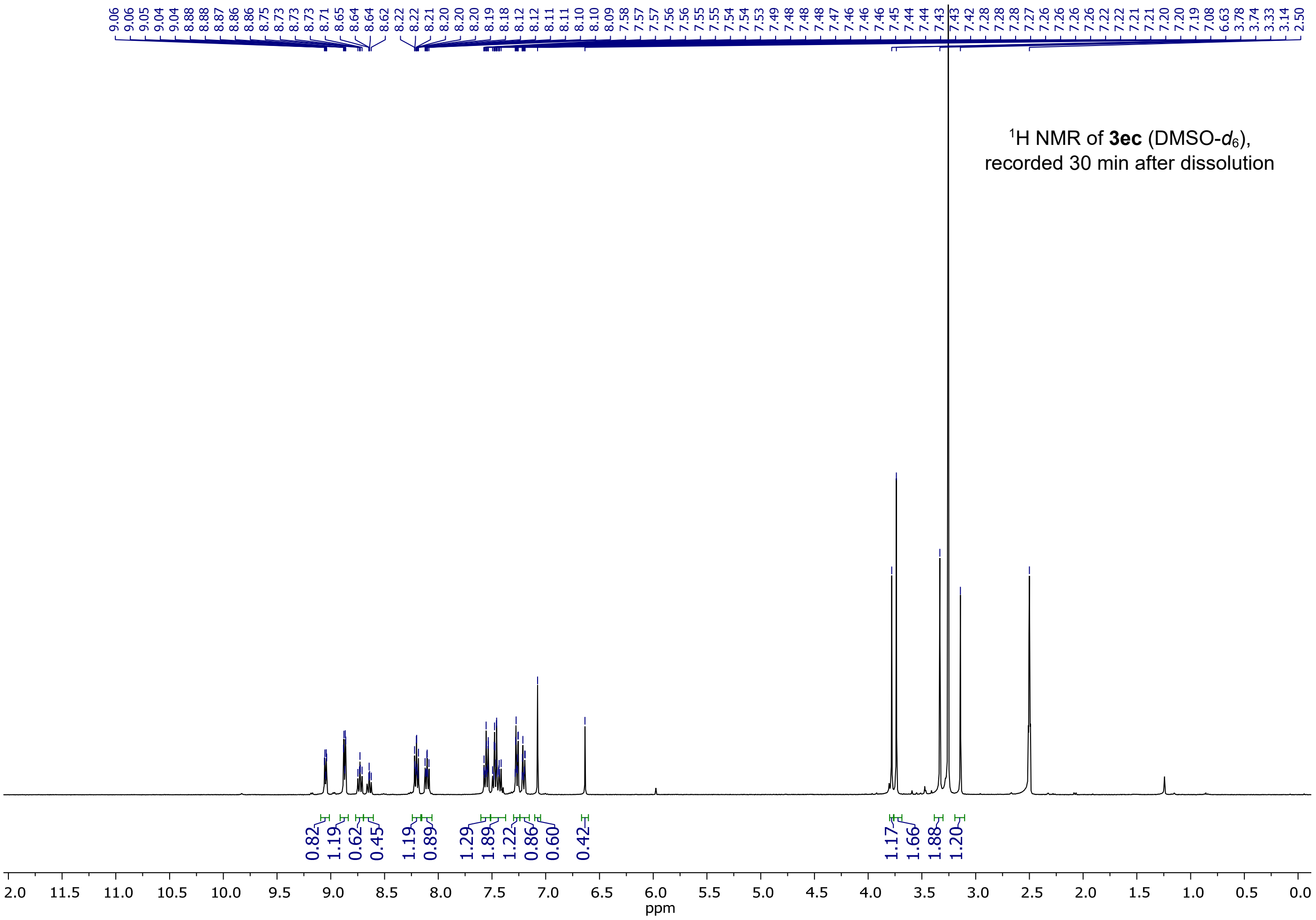
^{1}H NMR of **3ec** (DMSO-d_6),
recorded 30 min after dissolution
ppm

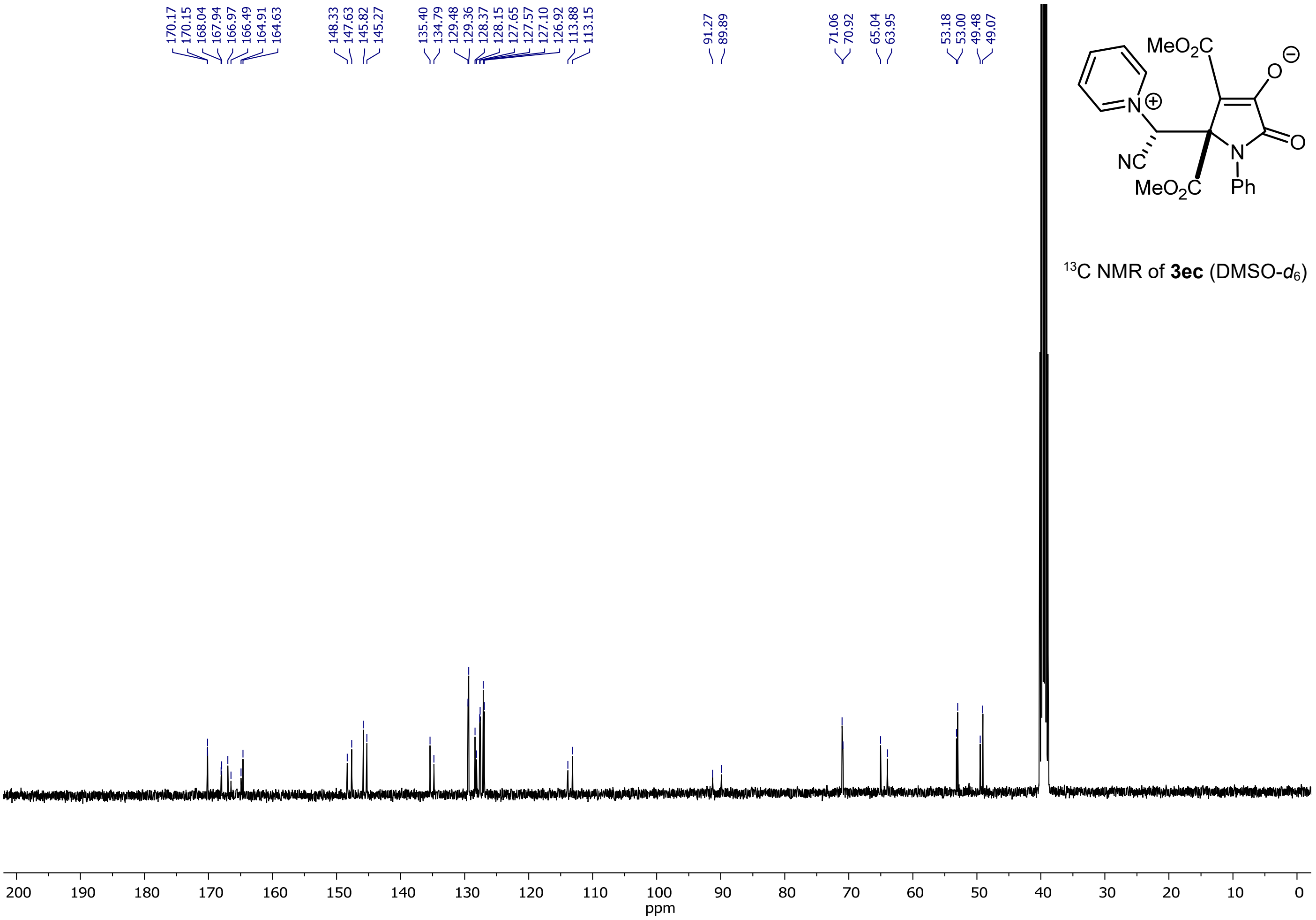

^{13}C NMR of **3ec** (DMSO-d_6)

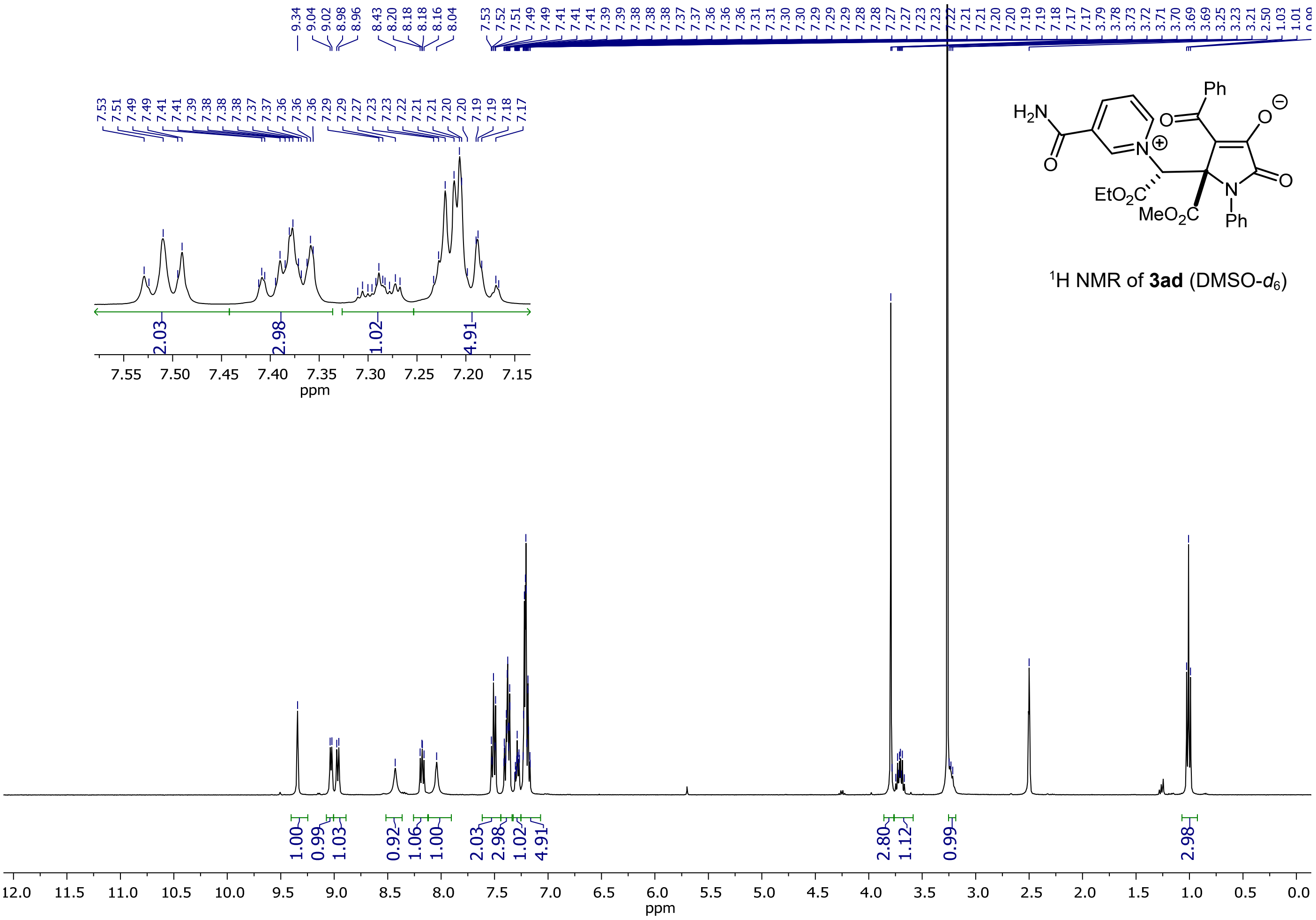

¹H NMR of **3ad** (DMSO-d_6)
ppm

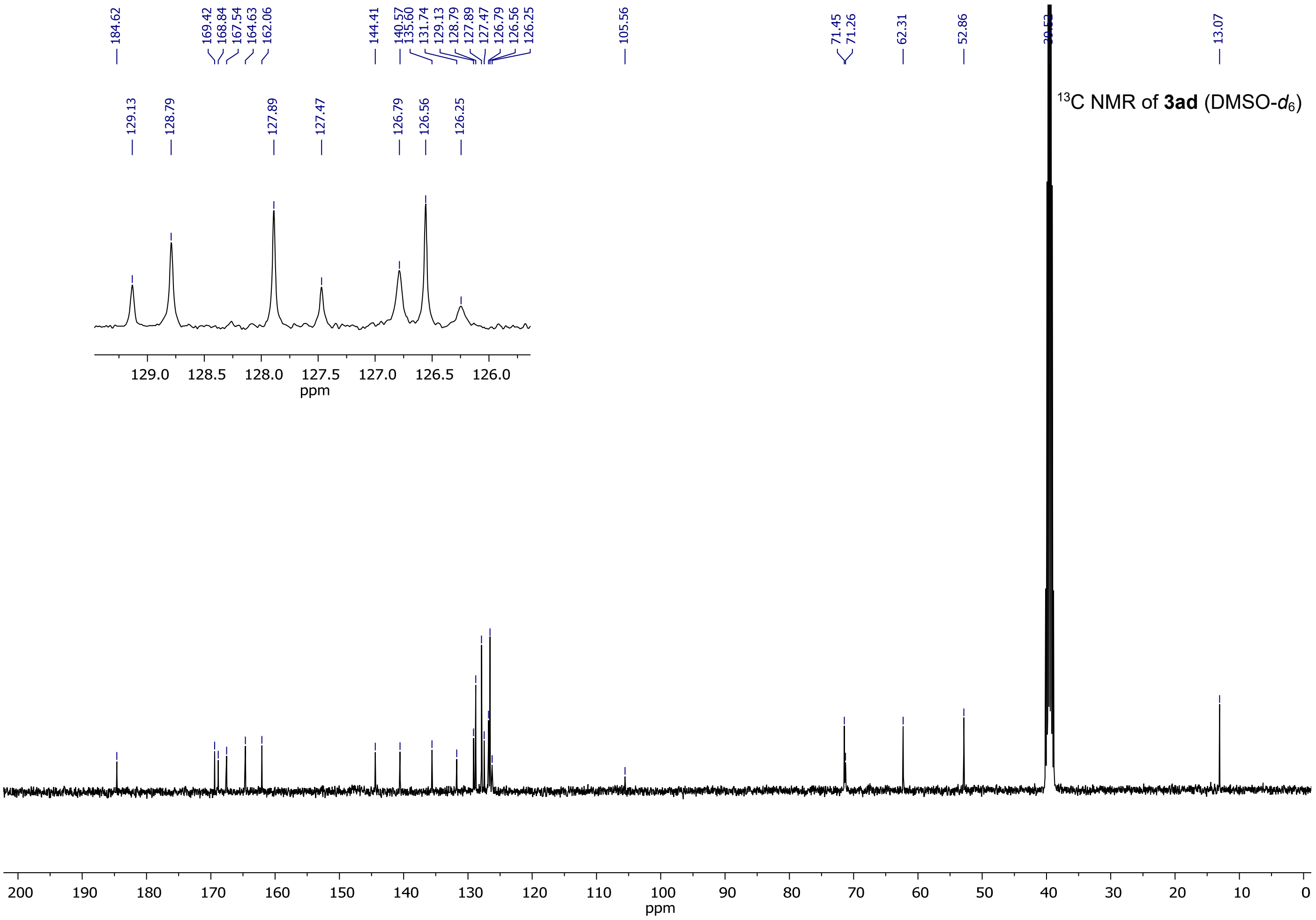

¹³C NMR of **3ad** (DMSO-d_6)
ppm

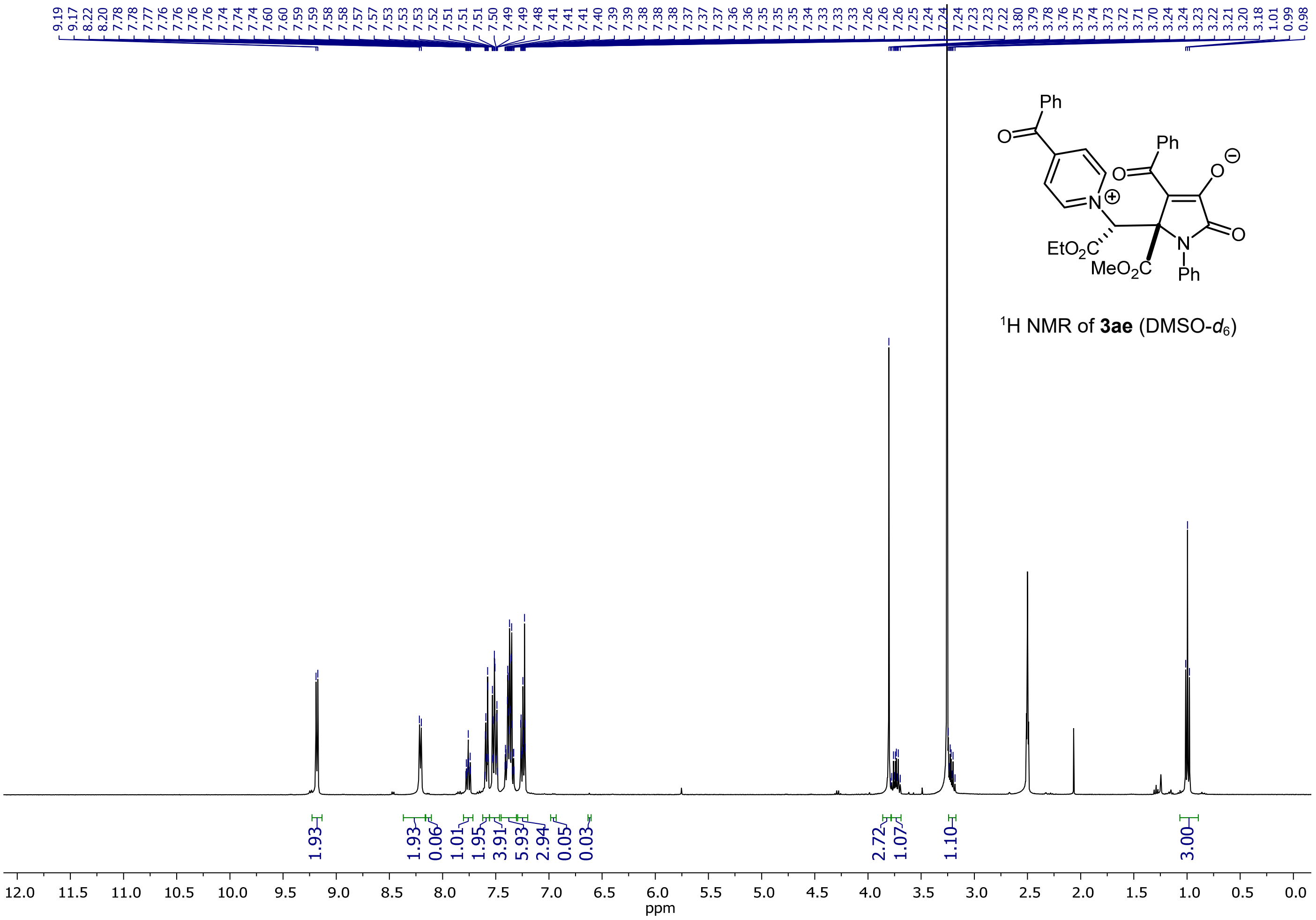

^{1}H NMR of **3ae** (DMSO-d_6)

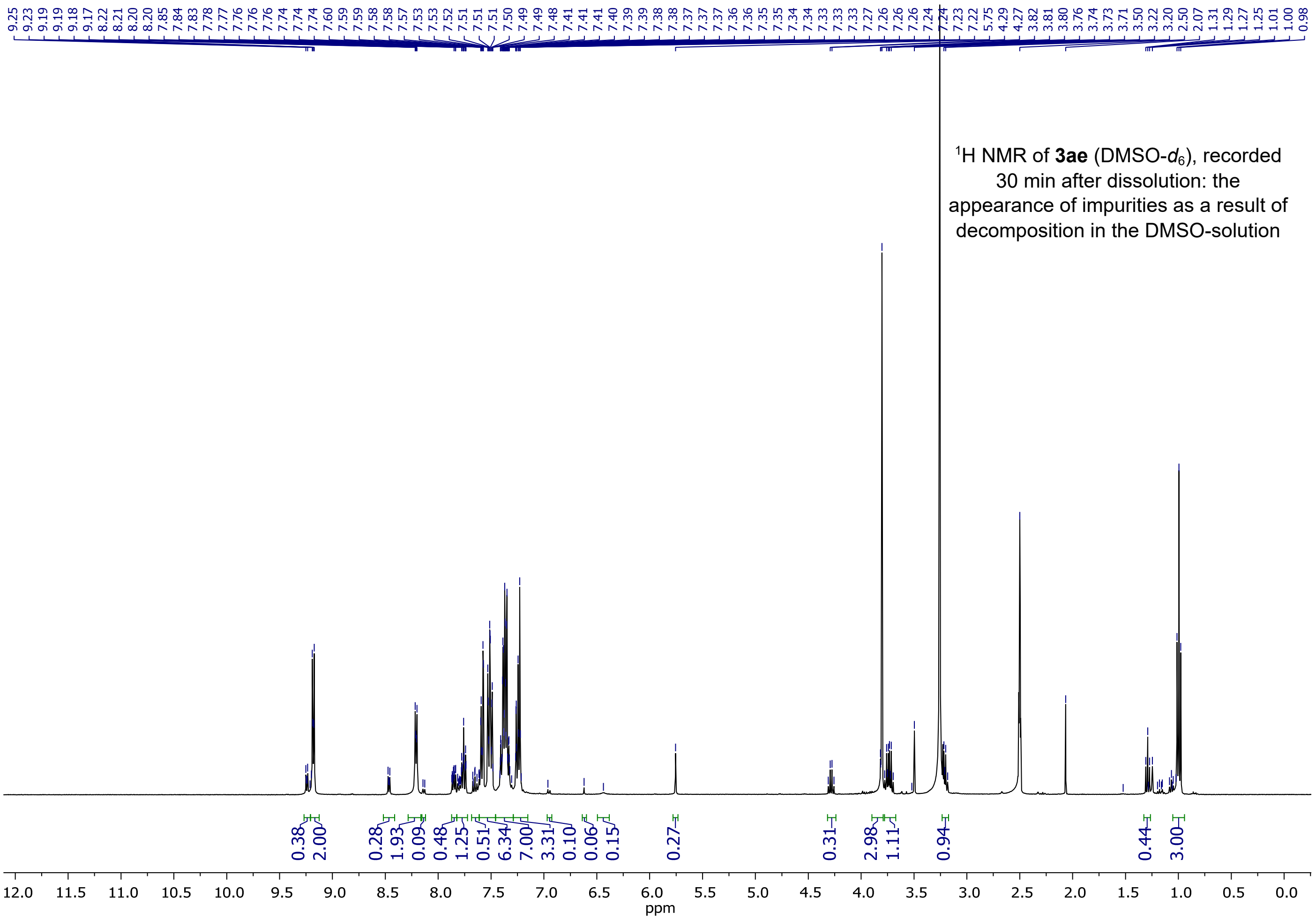

^{1}H NMR of **3ae** (DMSO-d_6), recorded 30 min after dissolution: the appearance of impurities as a result of decomposition in the DMSO-solution

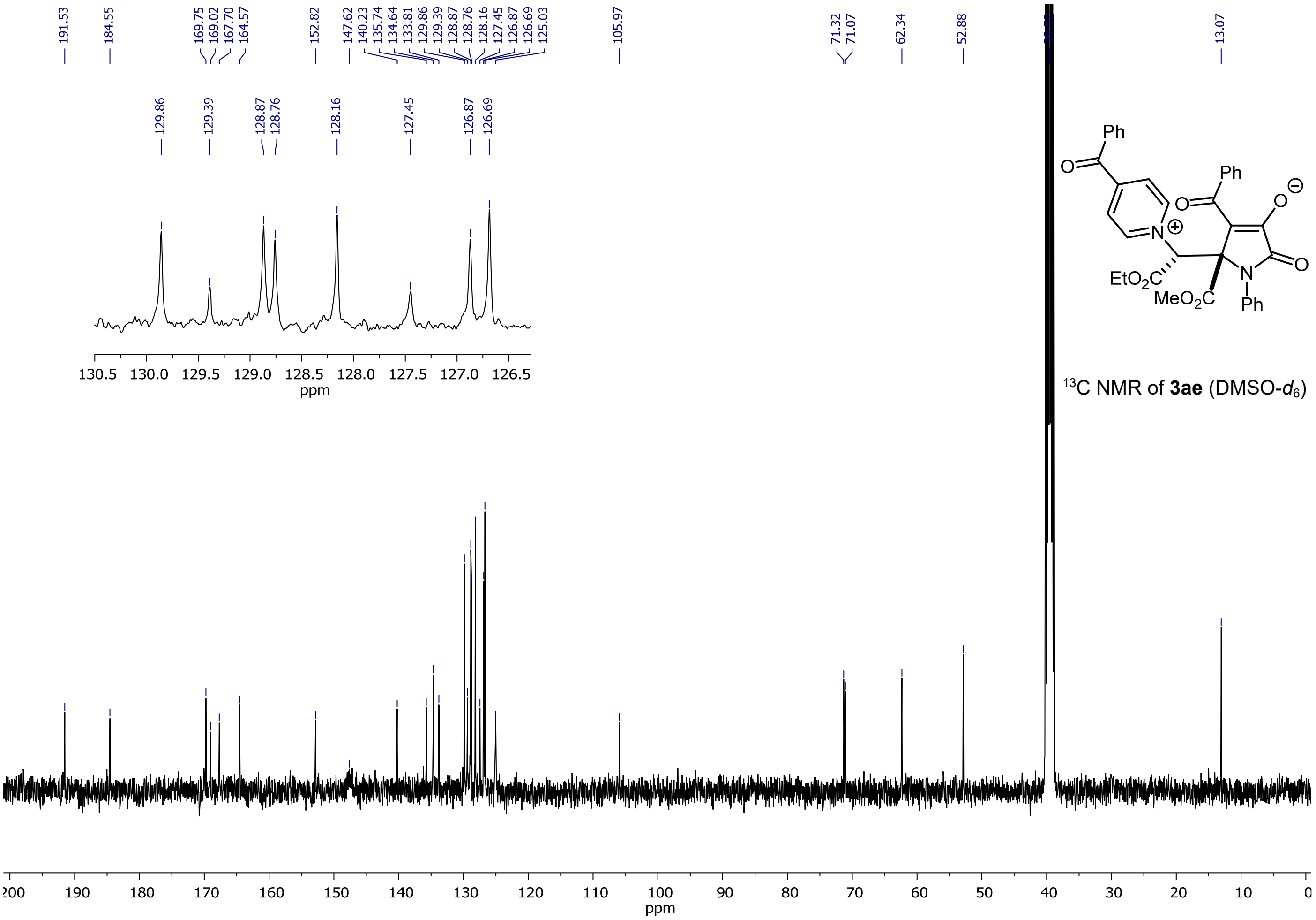

^{13}C NMR of **3ae** (DMSO-d_6)

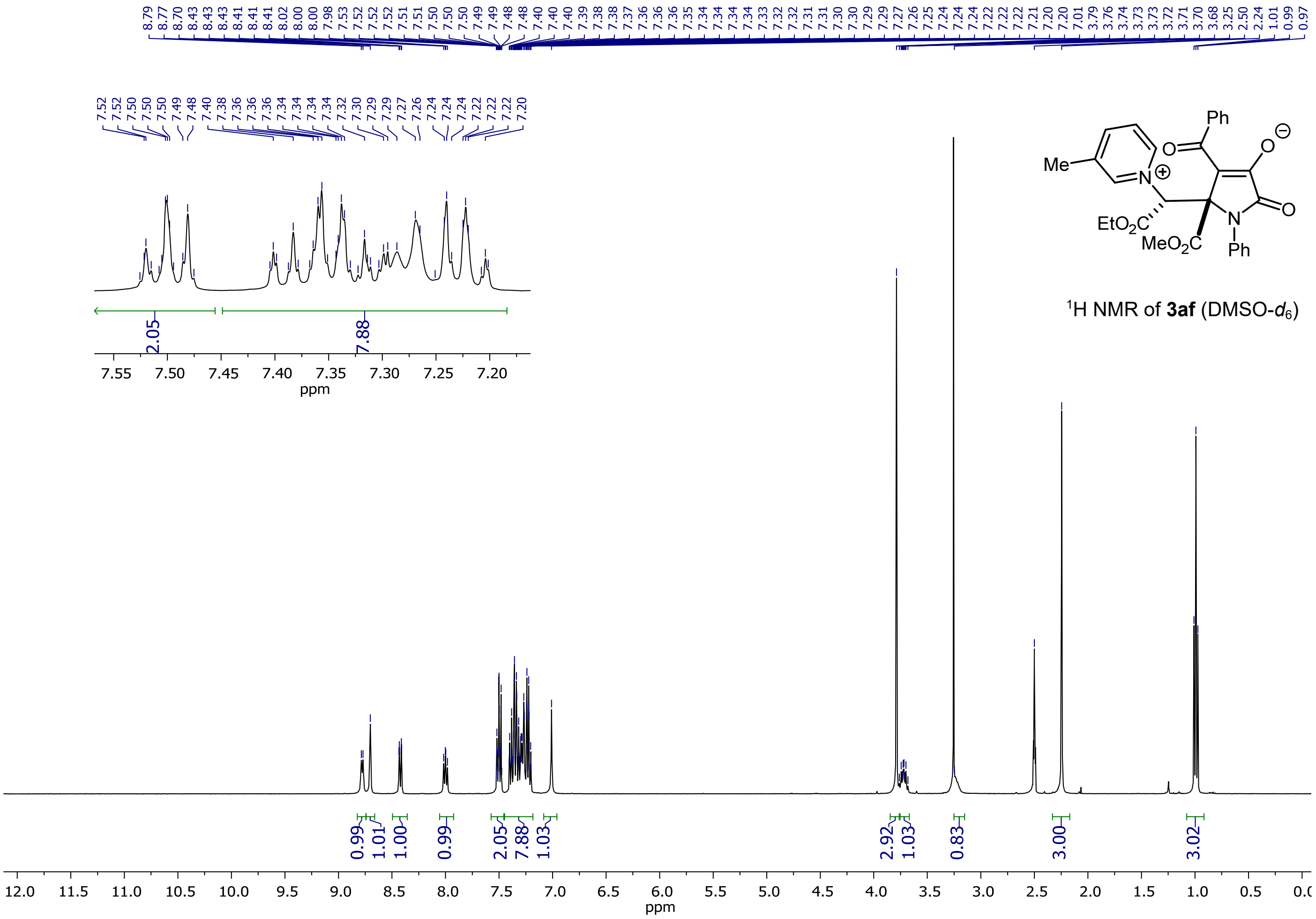
8.79 8.77 8.70 8.43 8.43 8.43 8.41 8.41 8.02 8.00 8.00 7.98 7.53 7.52 7.52 7.52 7.51 7.51 7.50 7.50 7.50 7.49 7.48 7.48 7.40 7.40 7.39 7.38 7.38 7.37 7.36 7.36 7.35 7.34 7.34 7.34 7.34 7.33 7.32 7.32 7.31 7.31 7.30 7.30 7.29 7.29 7.27 7.26 7.25 7.24 7.24 7.24 7.22 7.22 7.22 7.21 7.20 7.20 7.01 3.79 3.76 3.74 3.73 3.73 3.72 3.71 3.70 3.68 3.25 2.50 2.24 1.01 0.99 0.97
Ph
O
Me
N
EtO2C
MeO2C
N
Ph
O
^{1}H NMR of **3af** (DMSO-d_6)
2.05
7.88
0.99 1.01 1.00 0.99 2.05 7.88 1.03 2.92 1.03 0.83 3.00 3.02
ppm

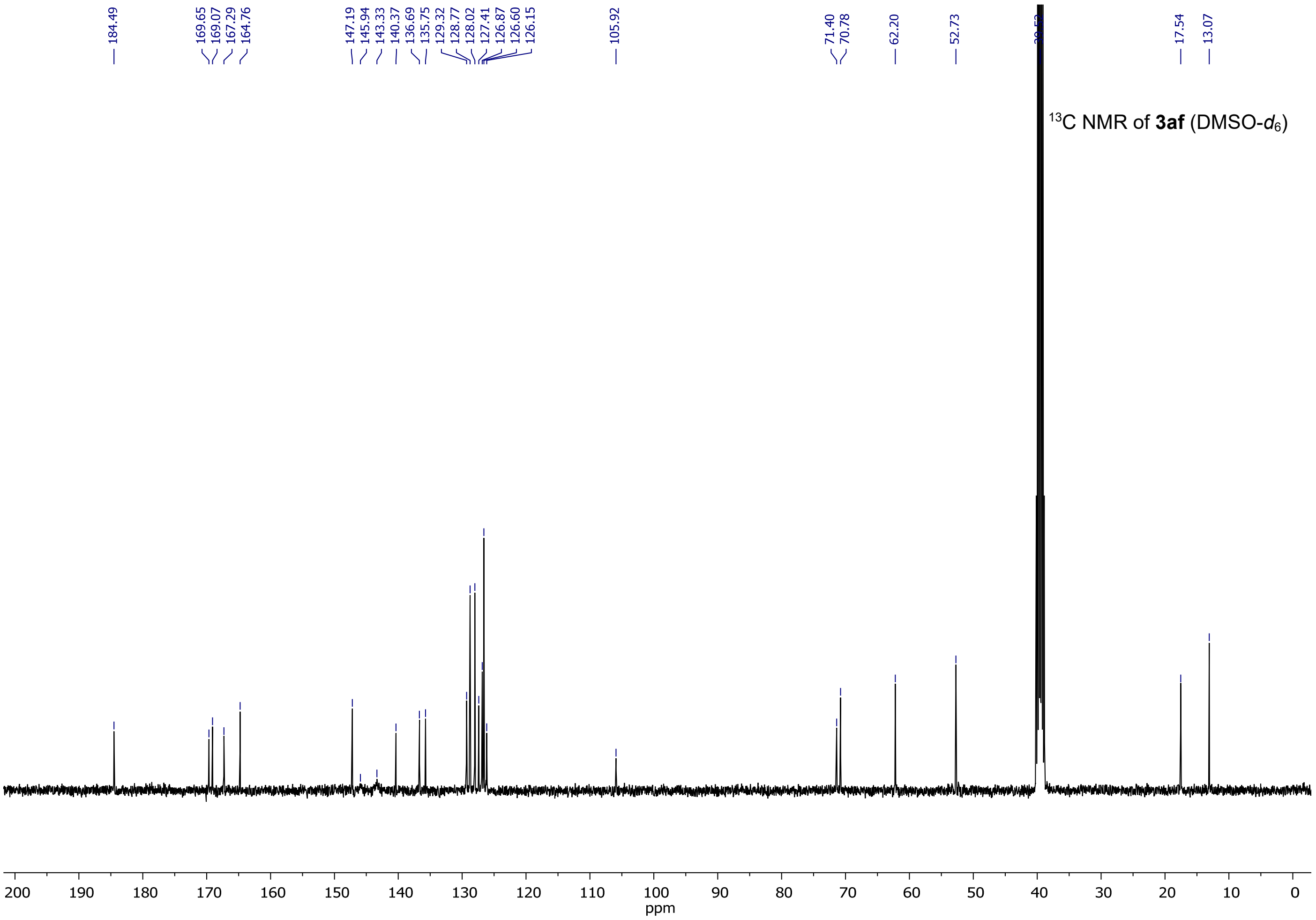
184.49
169.65 169.07 167.29 164.76
147.19 145.94 143.33 140.37 136.69 135.75 129.32 128.77 128.02 127.41 126.87 126.60 126.15
105.92
71.40 70.78
62.20
52.73
17.54
13.07
^{13}C NMR of **3af** (DMSO-d_6)
ppm

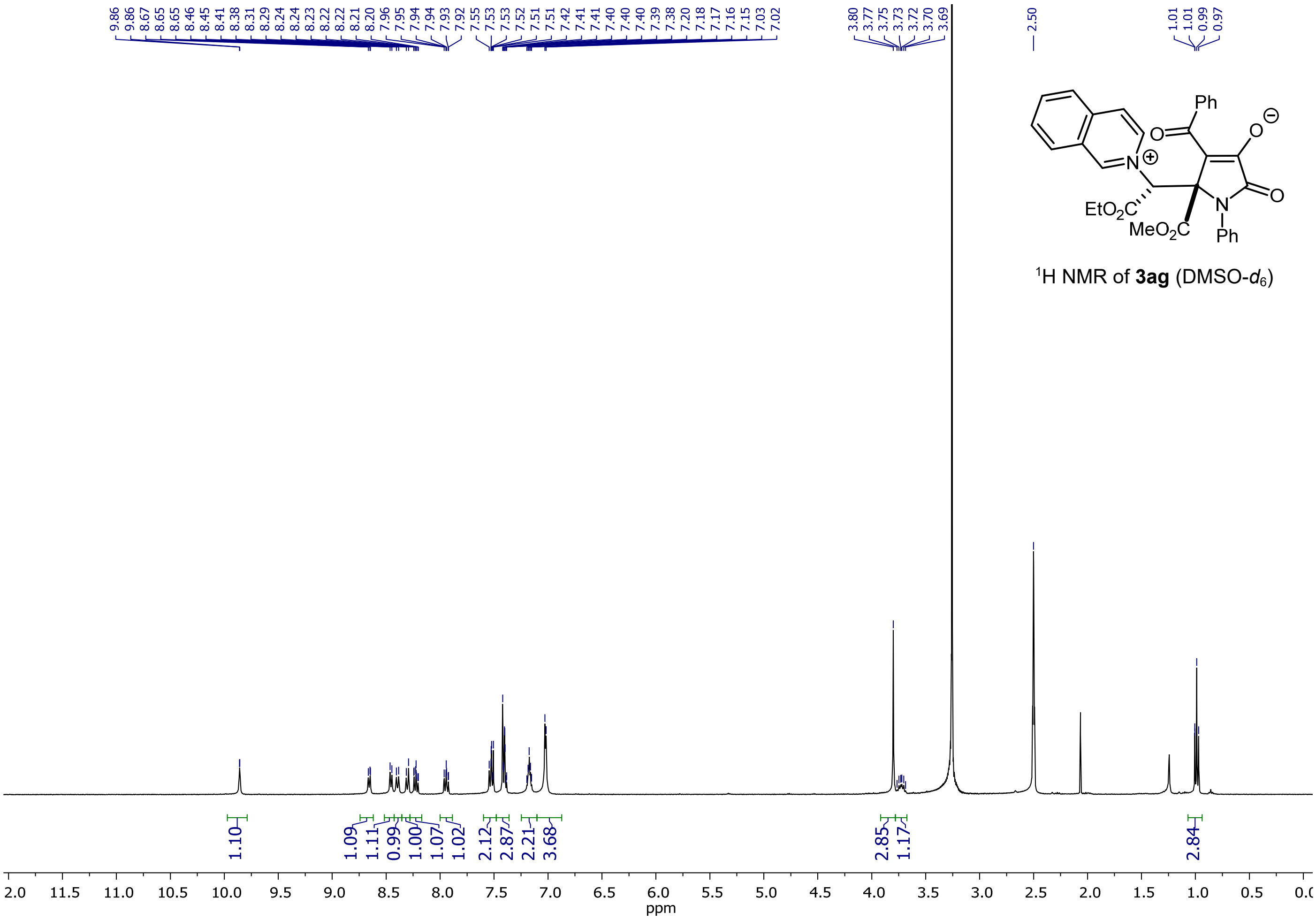

1H NMR of 3ag (DMSO-d6)
EtO2C
MeO2C
Ph
O
N

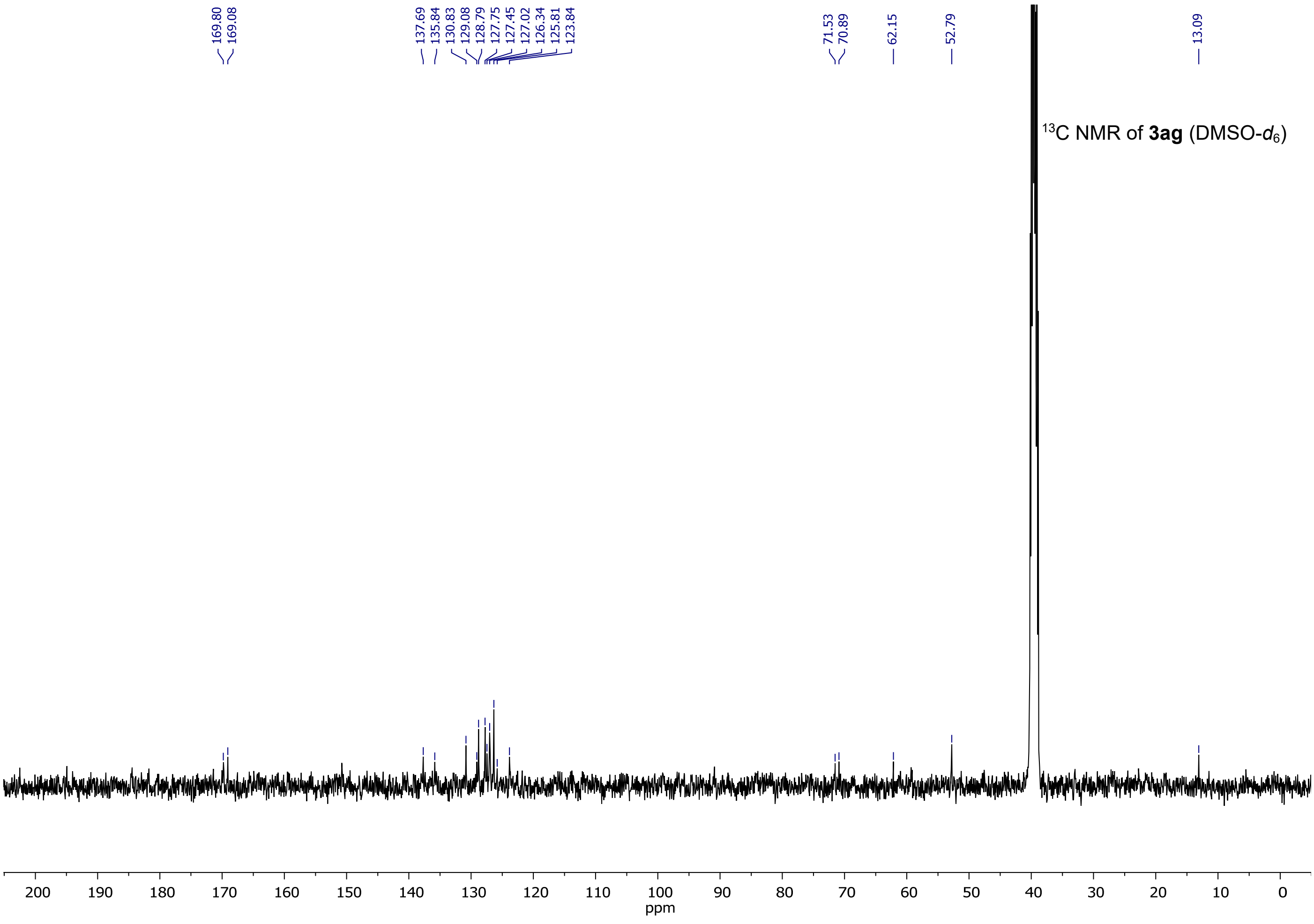

13C NMR of 3ag (DMSO-d6)
ppm

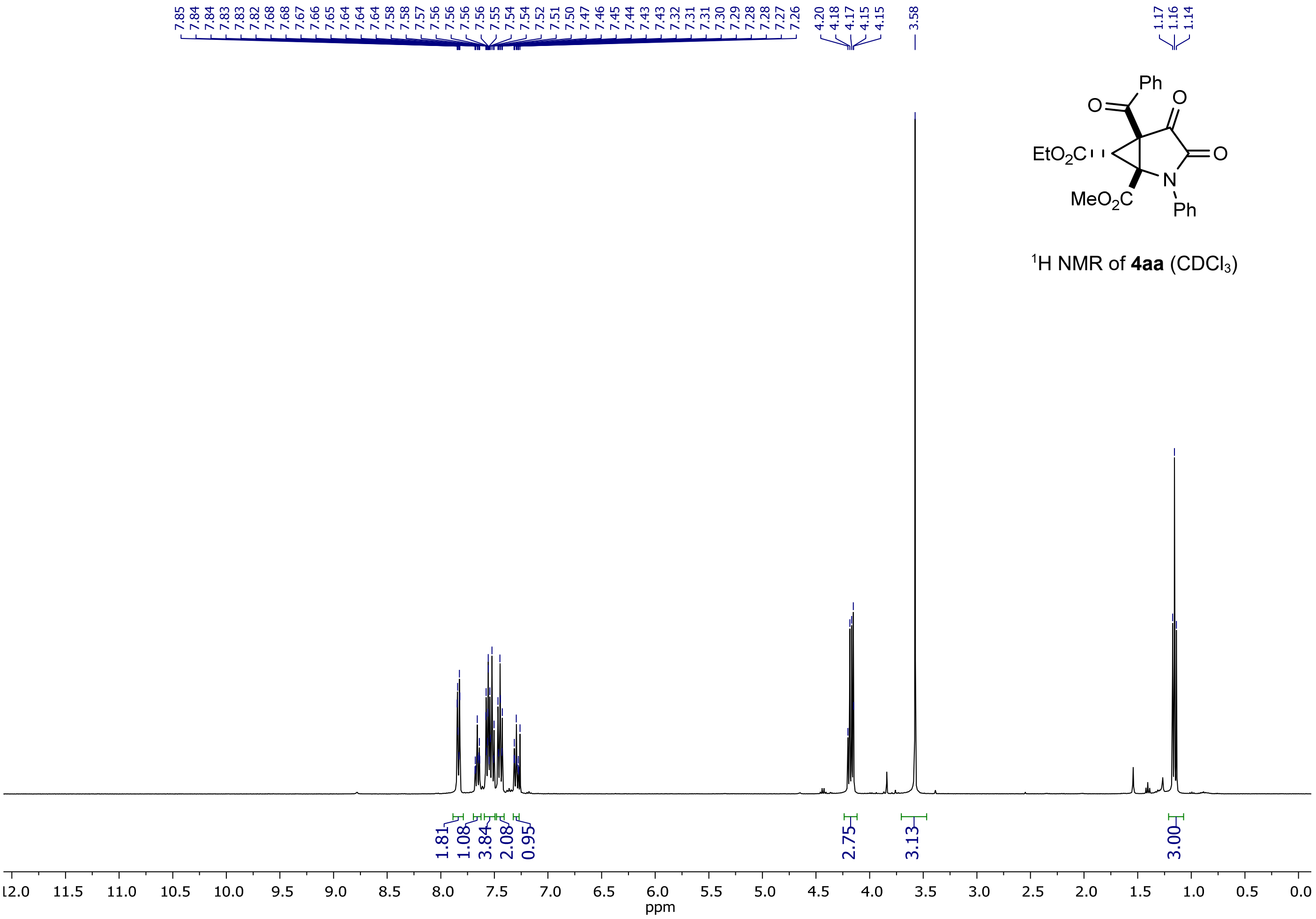

^1H NMR of **4aa** ($CDCl_3$)

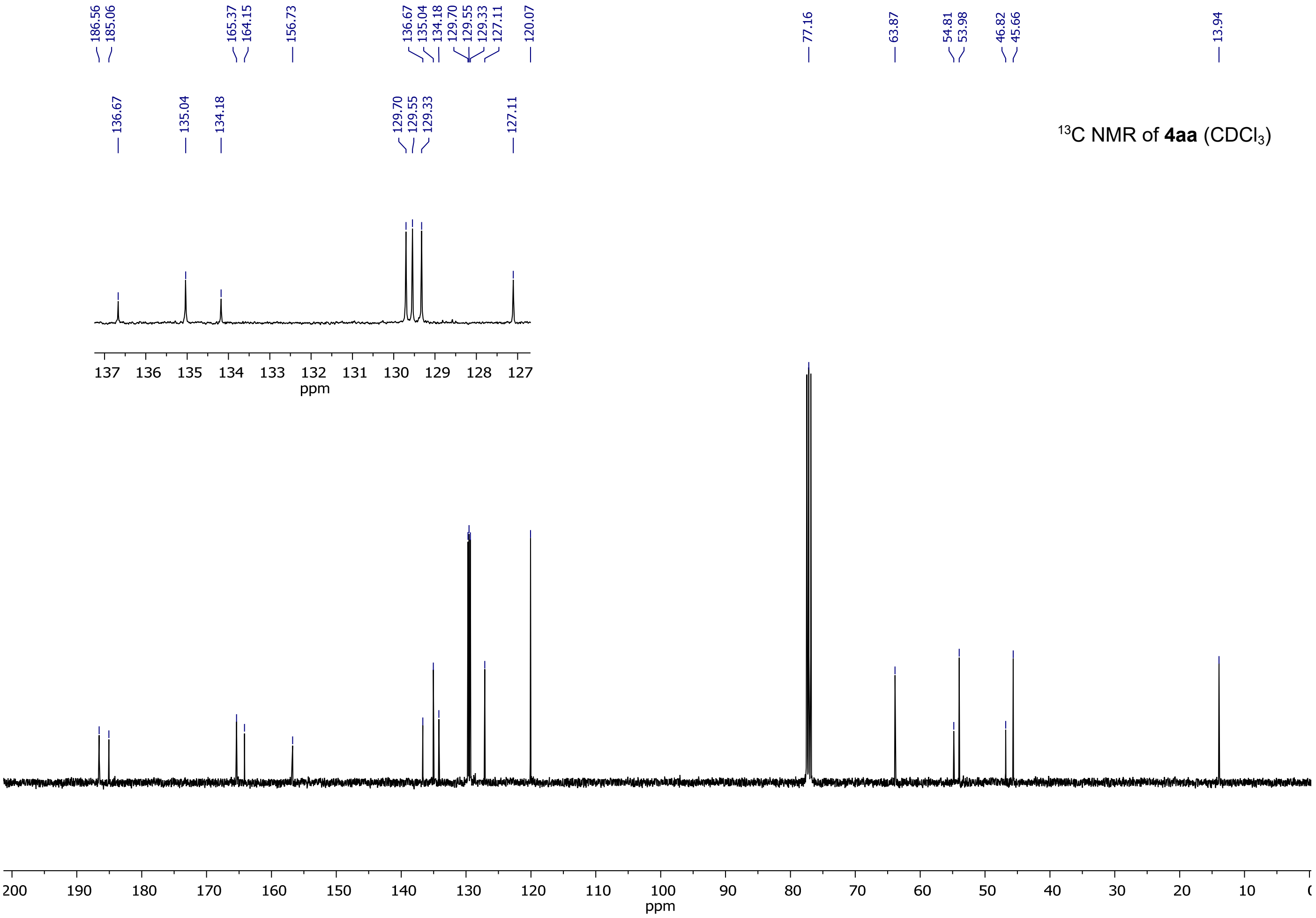

^{13}C NMR of **4aa** ($CDCl_3$)

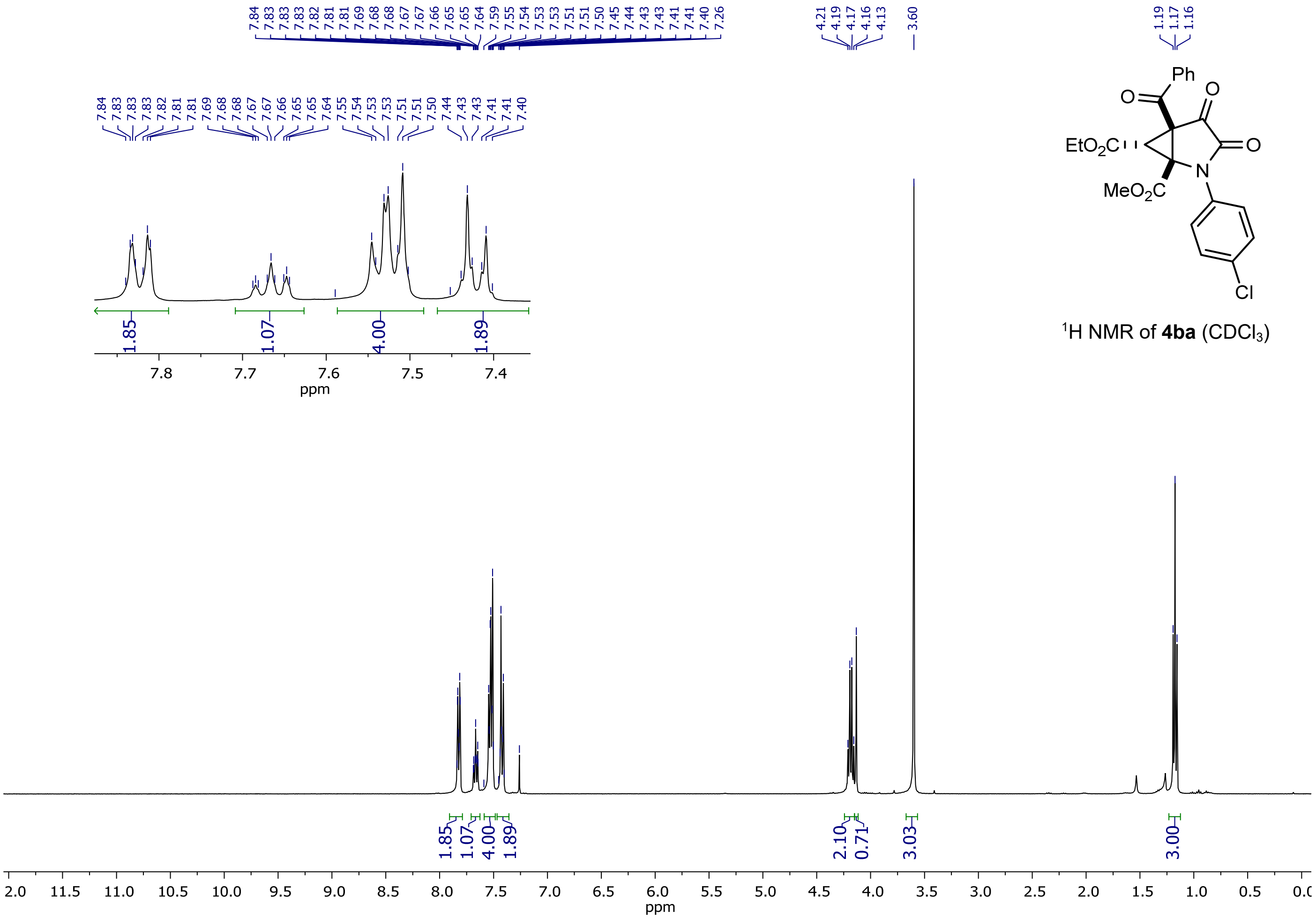

^{1}H NMR of **4ba** ($CDCl_3$)

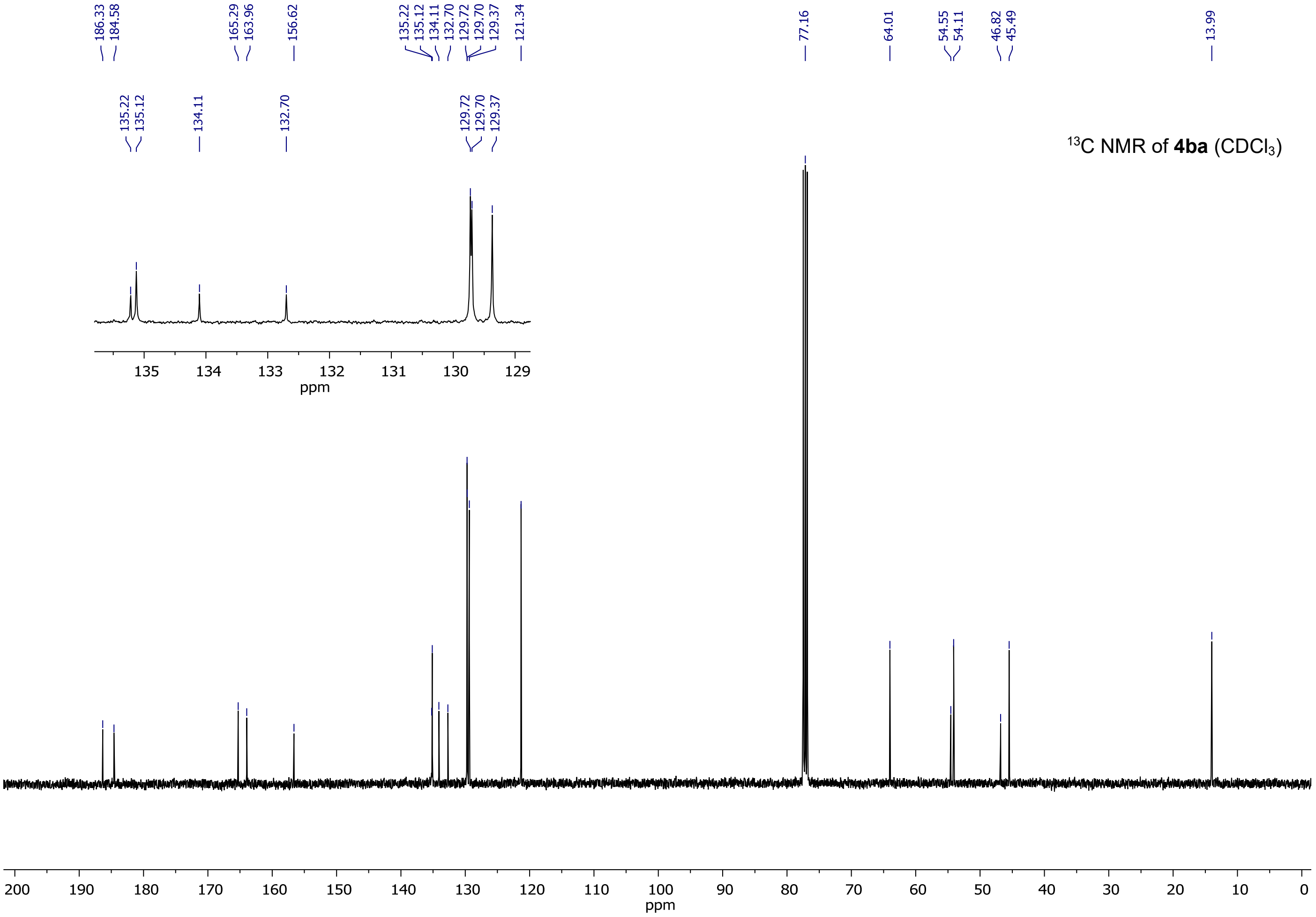

^{13}C NMR of **4ba** ($CDCl_3$)

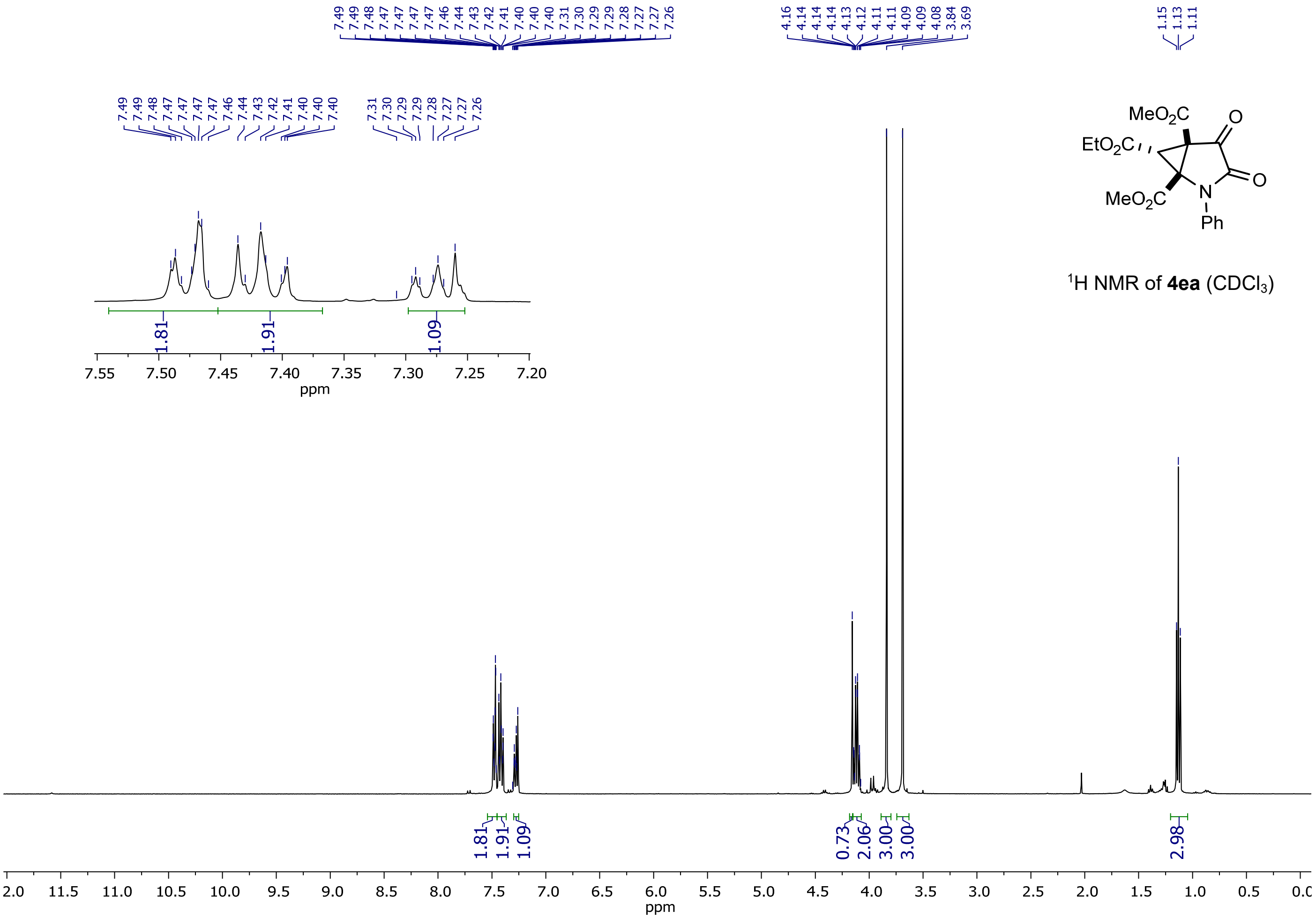
MeO2C
EtO2C
MeO2C
N
Ph
O
O
1H NMR of 4ea (CDCl3)
ppm

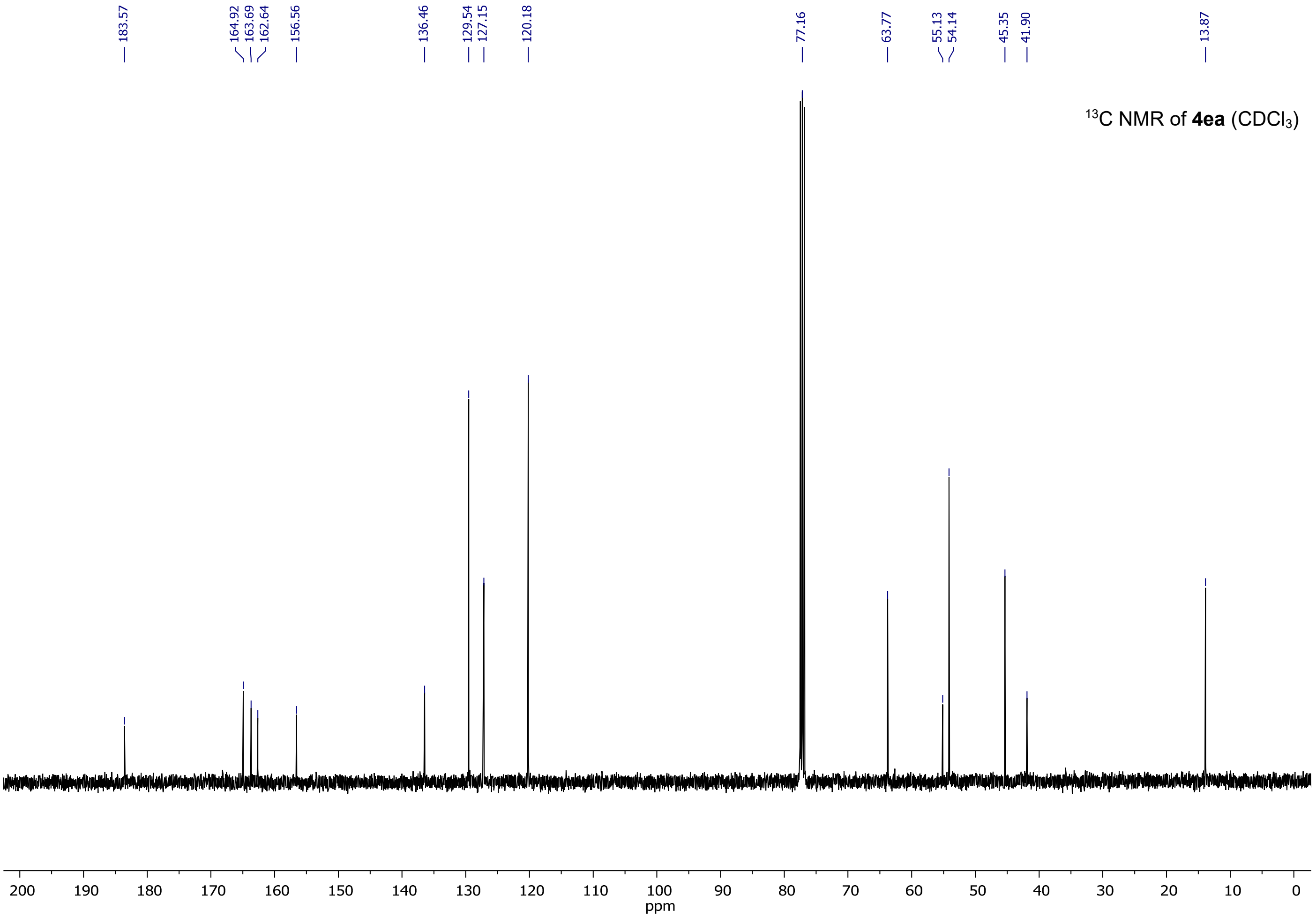
13C NMR of 4ea (CDCl3)
ppm

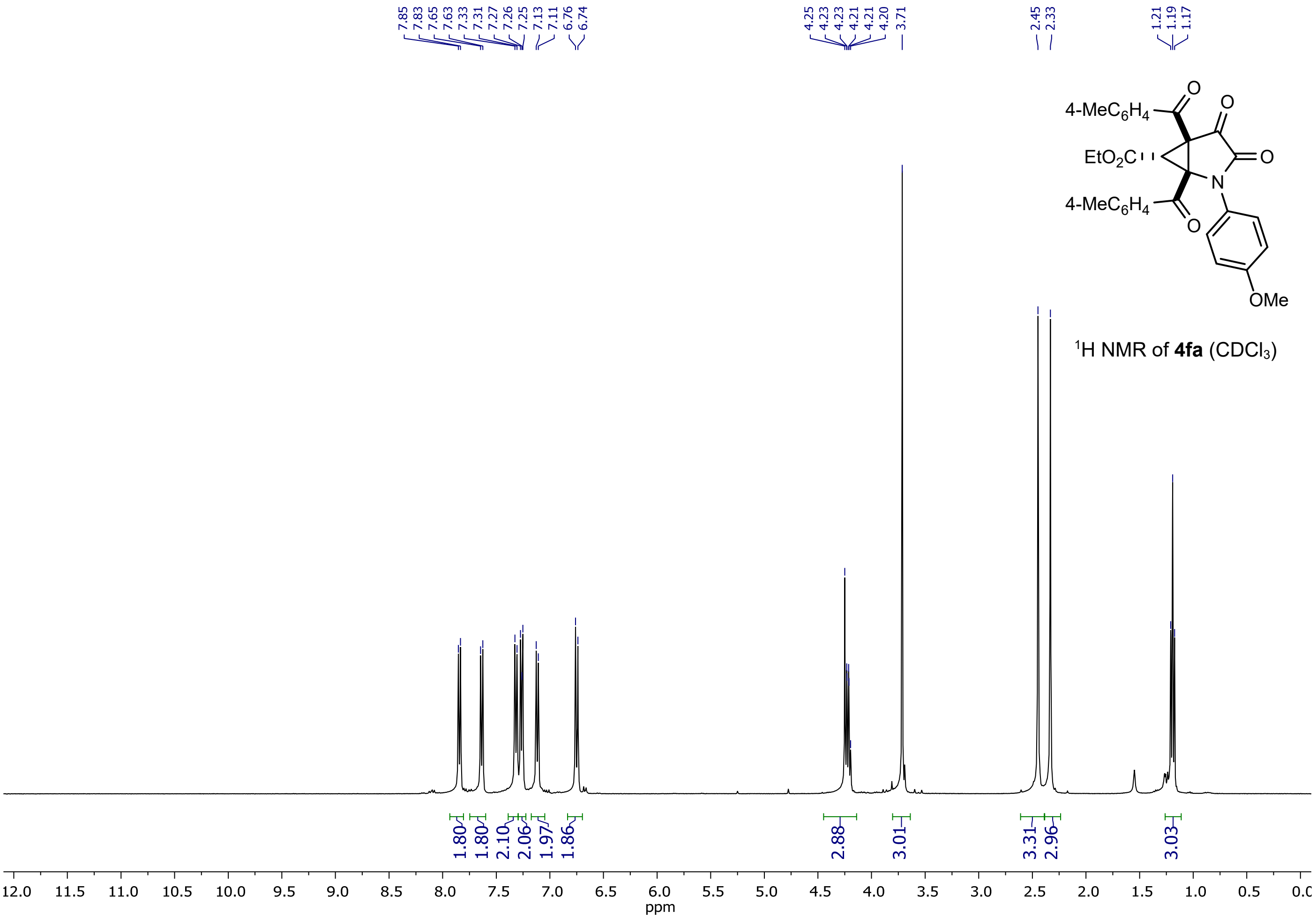

^{1}H NMR of **4fa** ($CDCl_3$)

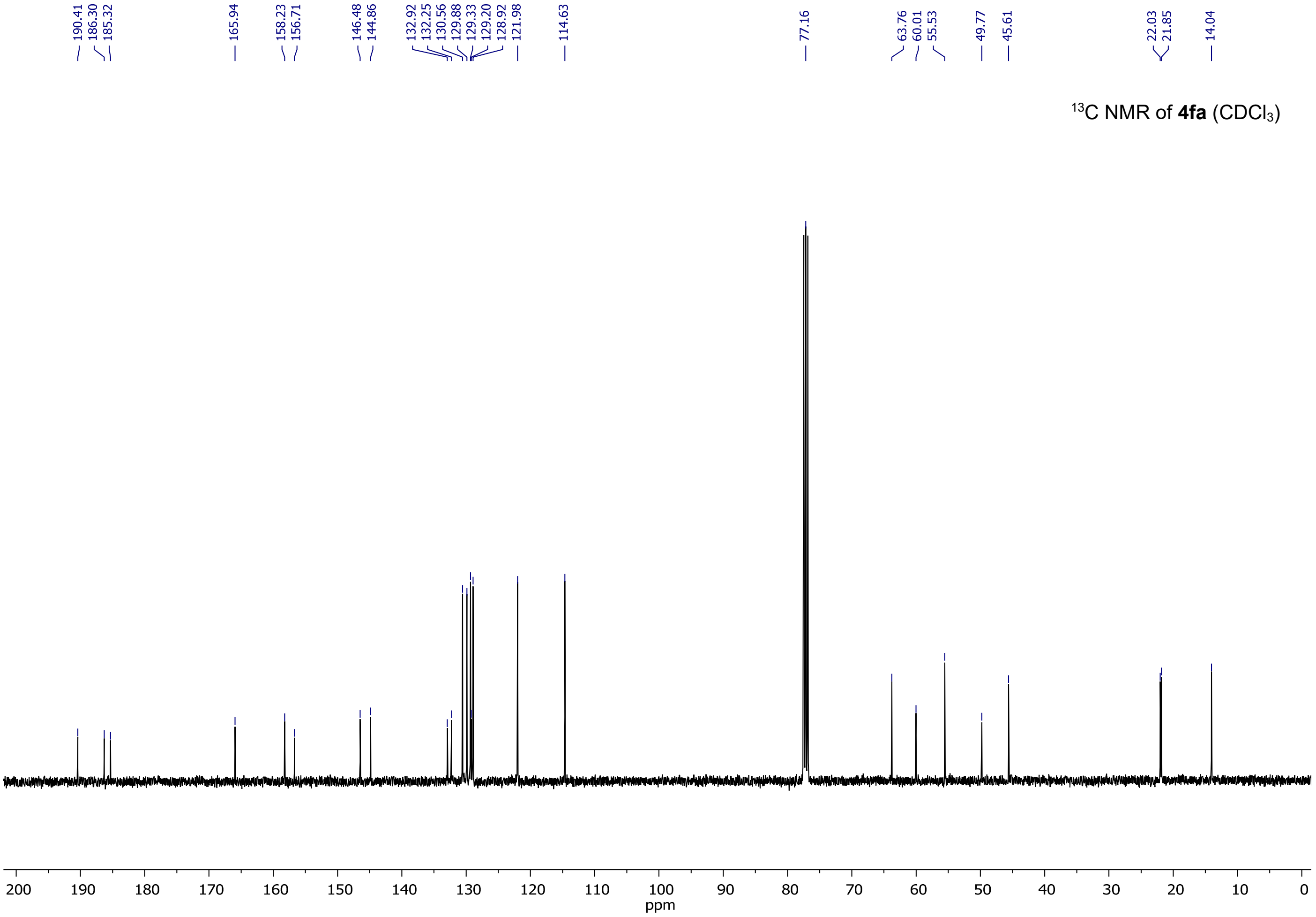

^{13}C NMR of **4fa** ($CDCl_3$)

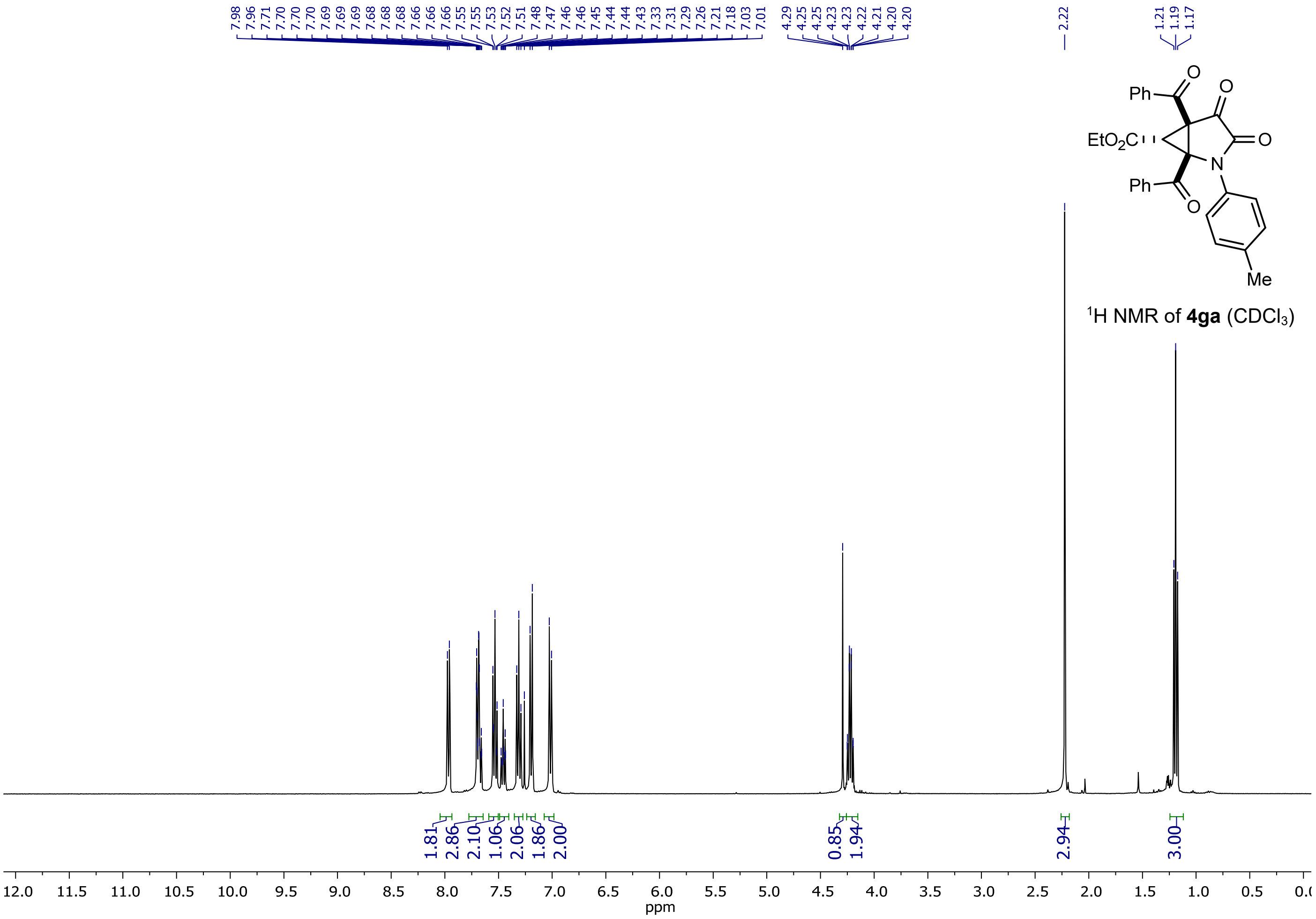
Ph
EtO2C
O
N
Me
1H NMR of 4ga (CDCl3)
ppm

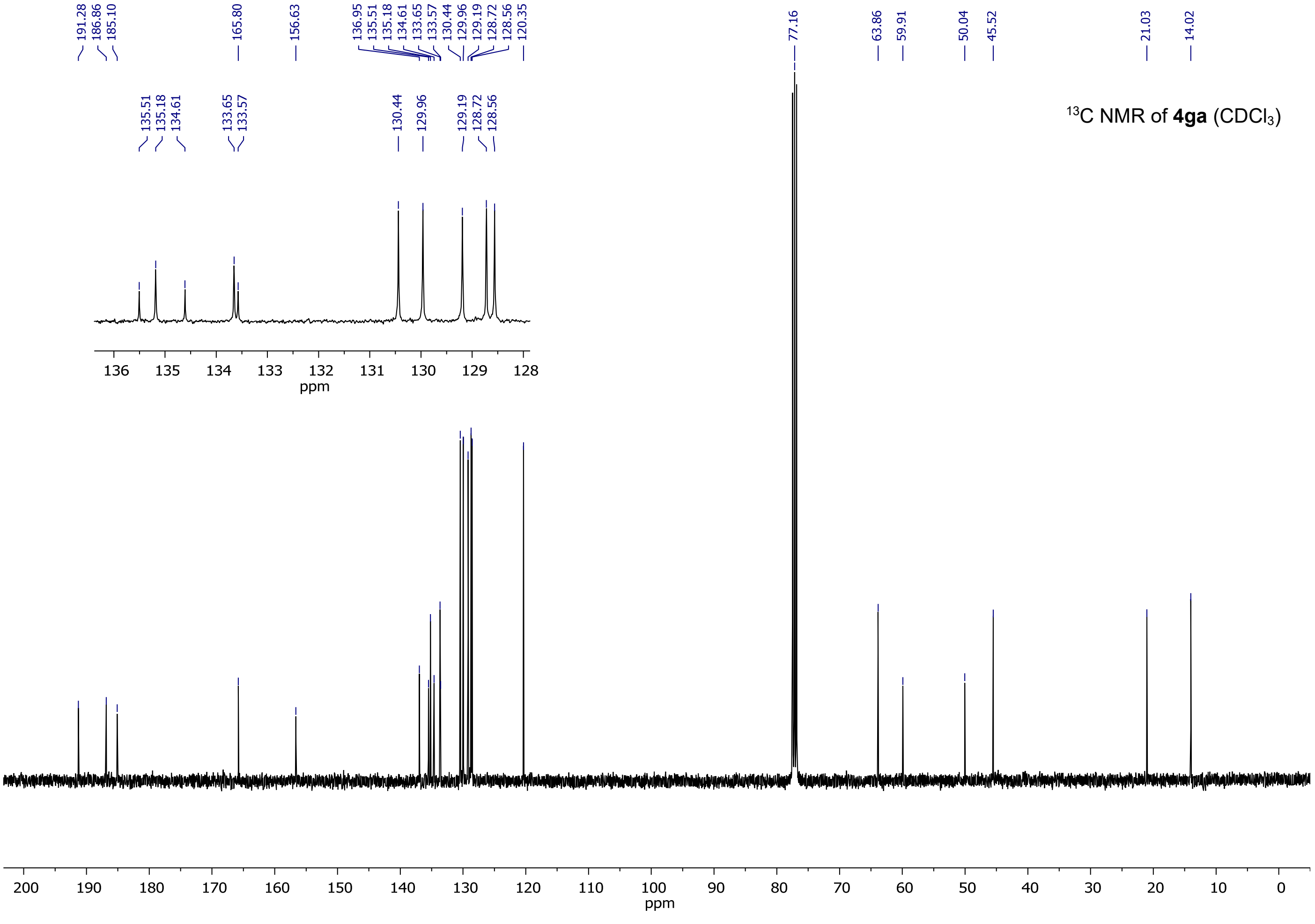
13C NMR of 4ga (CDCl3)
ppm

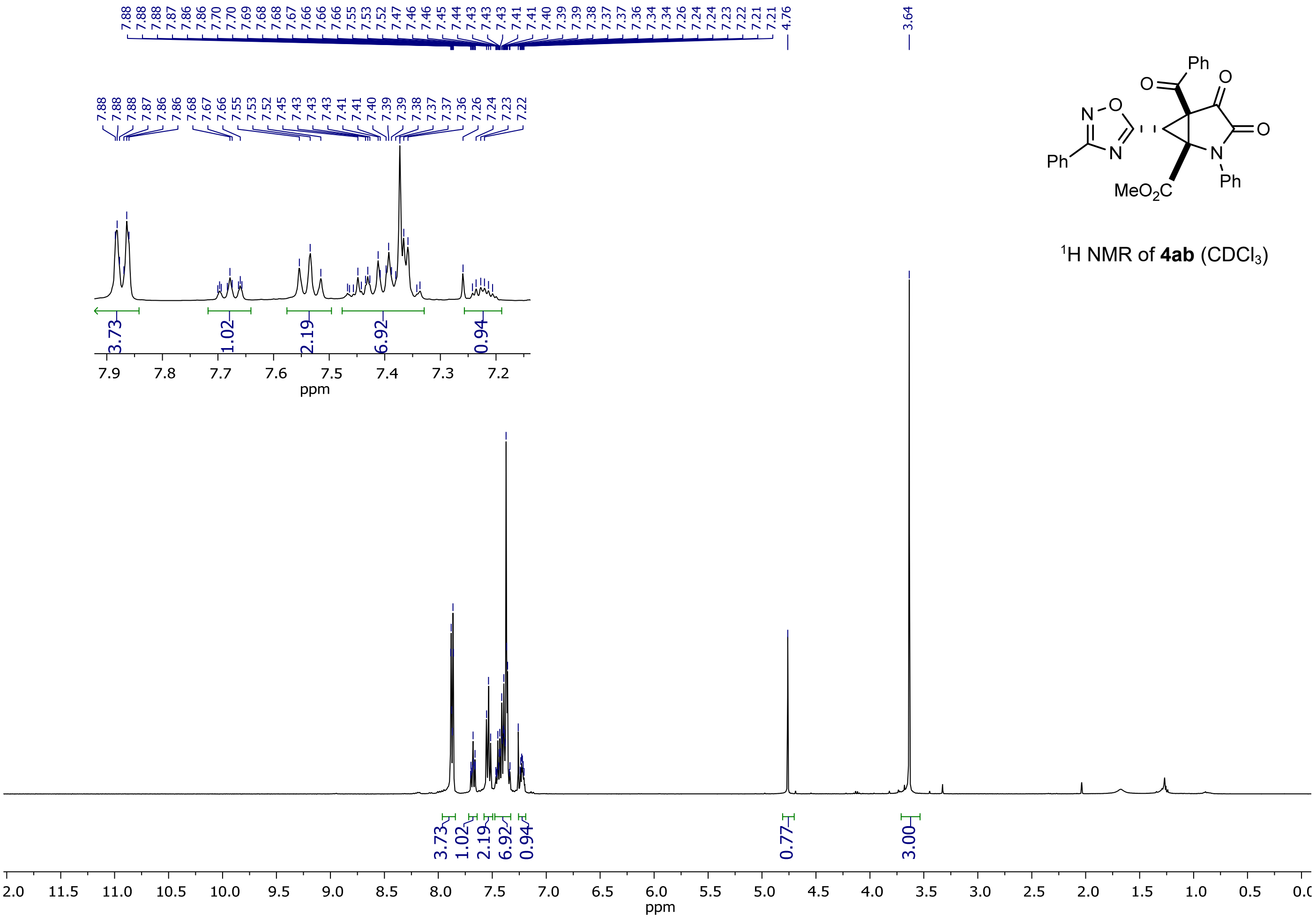

^{1}H NMR of **4ab** ($CDCl_3$)

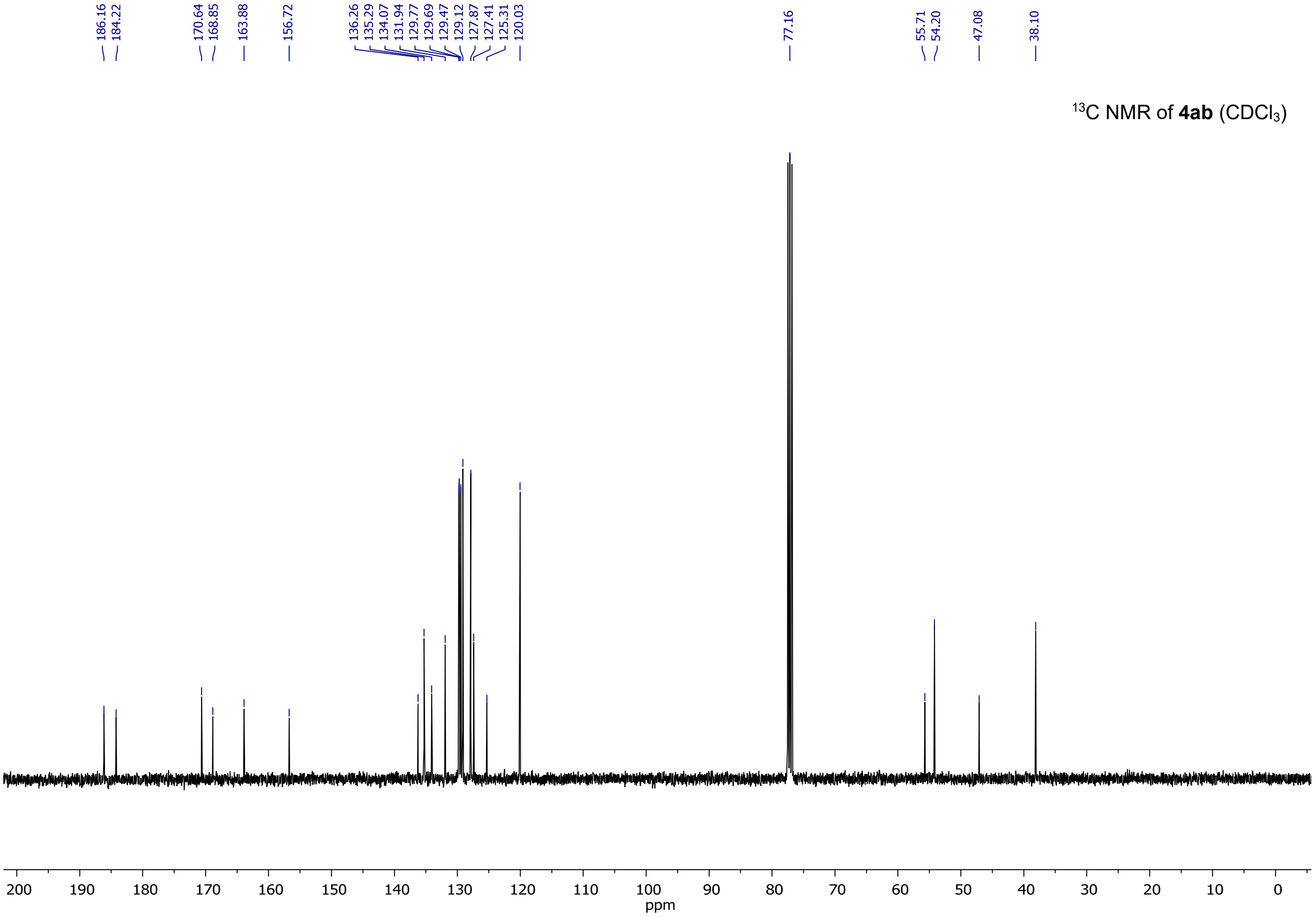

^{13}C NMR of **4ab** ($CDCl_3$)

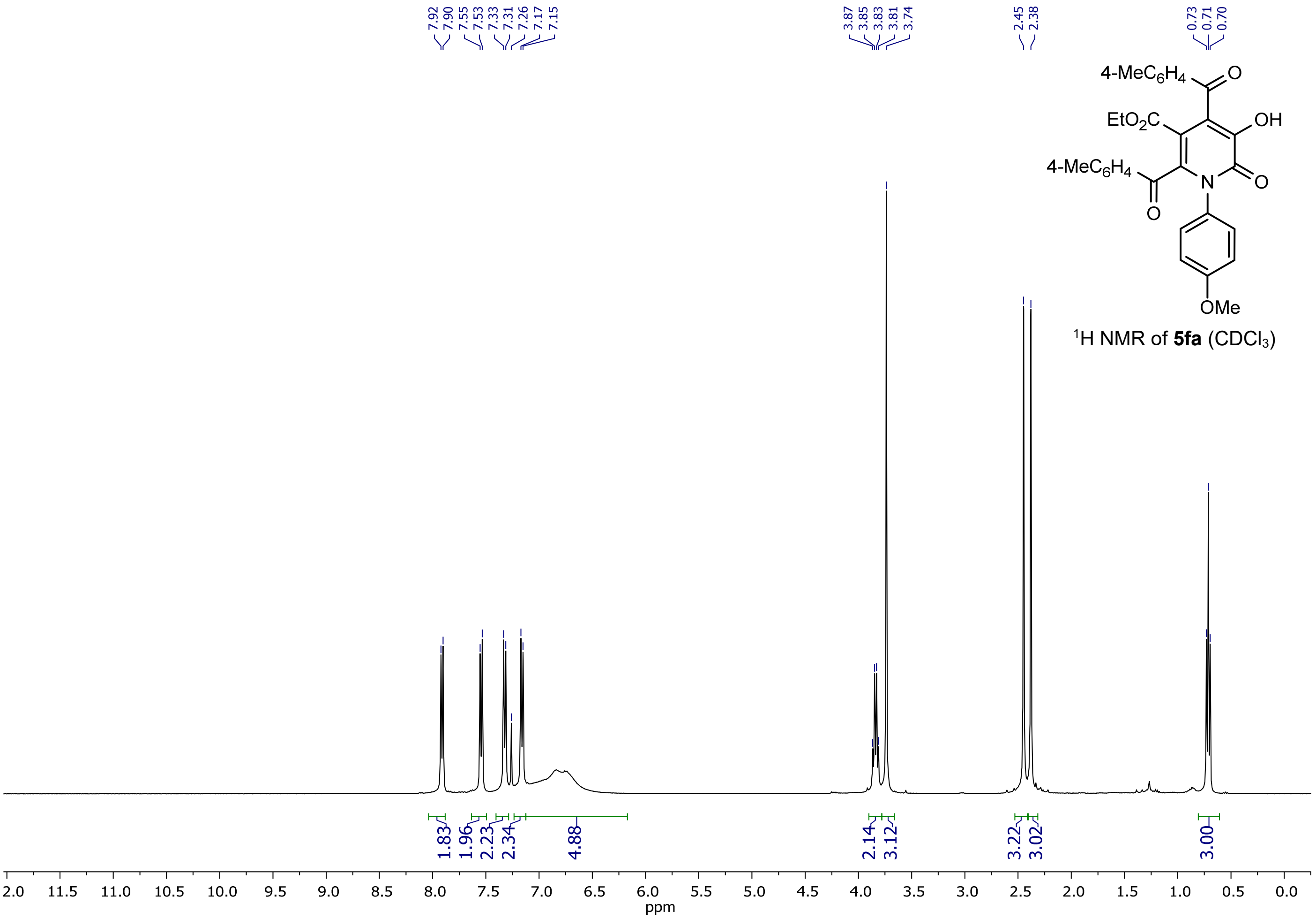
7.92
7.90
7.55
7.53
7.33
7.31
7.26
7.17
7.15
3.87
3.85
3.83
3.81
3.74
2.45
2.38
0.73
0.71
0.70
4-MeC6H4
O
EtO2C
OH
4-MeC6H4
N
O
O
OMe
1H NMR of 5fa (CDCl3)
1.83
1.96
2.23
2.34
4.88
2.14
3.12
3.22
3.02
3.00
ppm

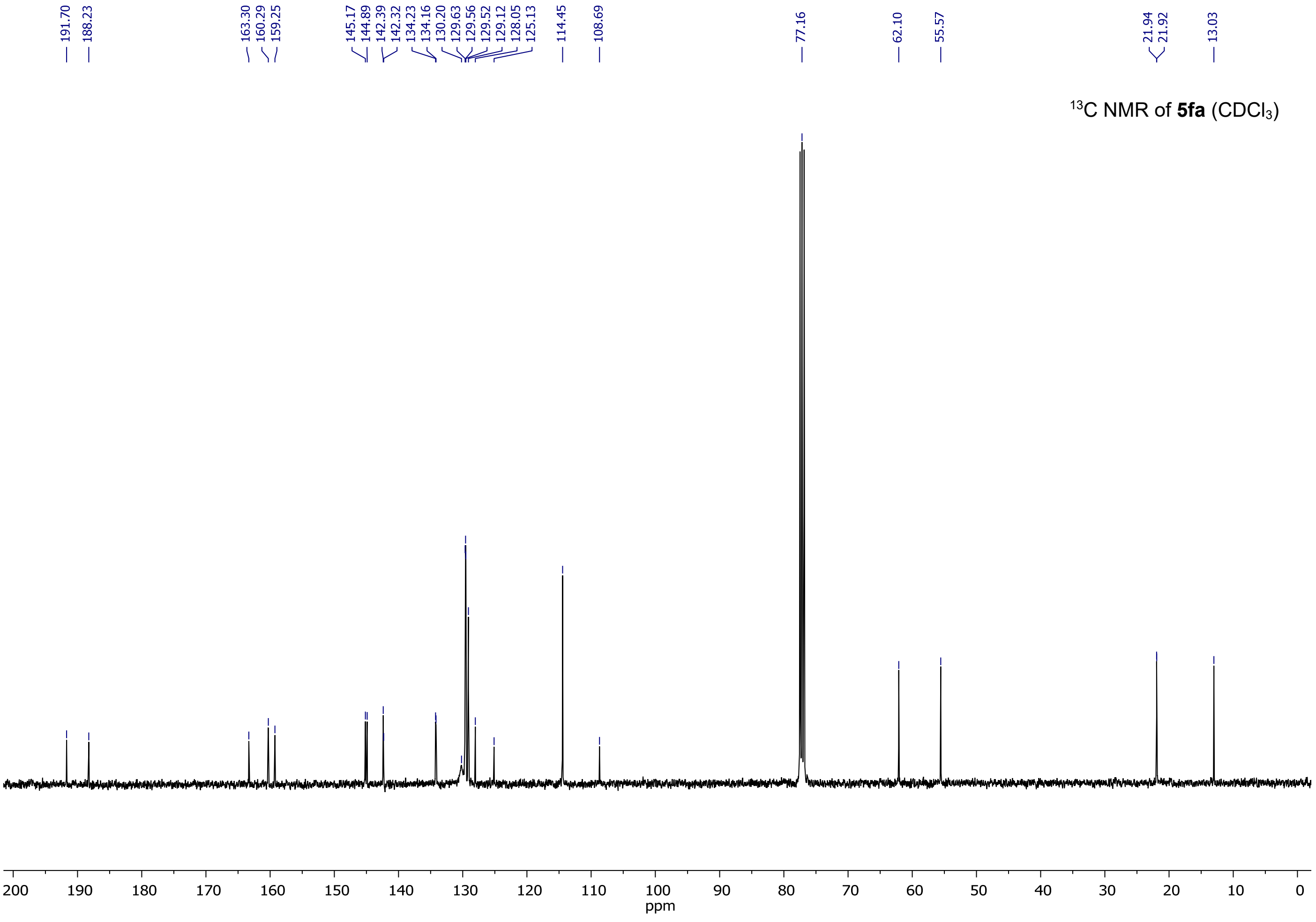
191.70
188.23
163.30
160.29
159.25
145.17
144.89
142.39
142.32
134.23
134.16
130.20
129.63
129.56
129.52
129.12
128.05
125.13
114.45
108.69
77.16
62.10
55.57
21.94
21.92
13.03
13C NMR of 5fa (CDCl3)
ppm

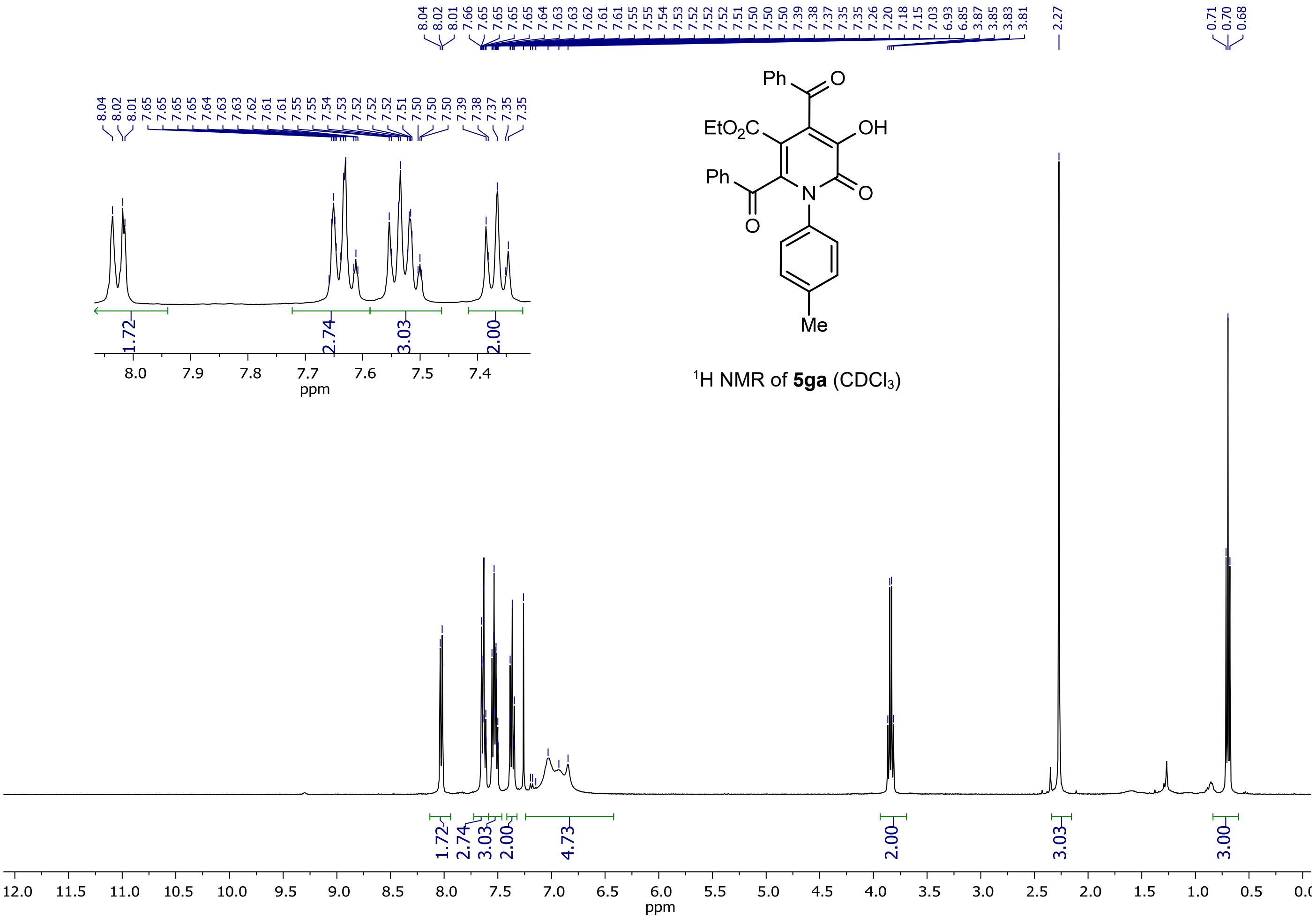
Ph
EtO2C
OH
Ph
N
O
Me
1H NMR of 5ga (CDCl3)
ppm

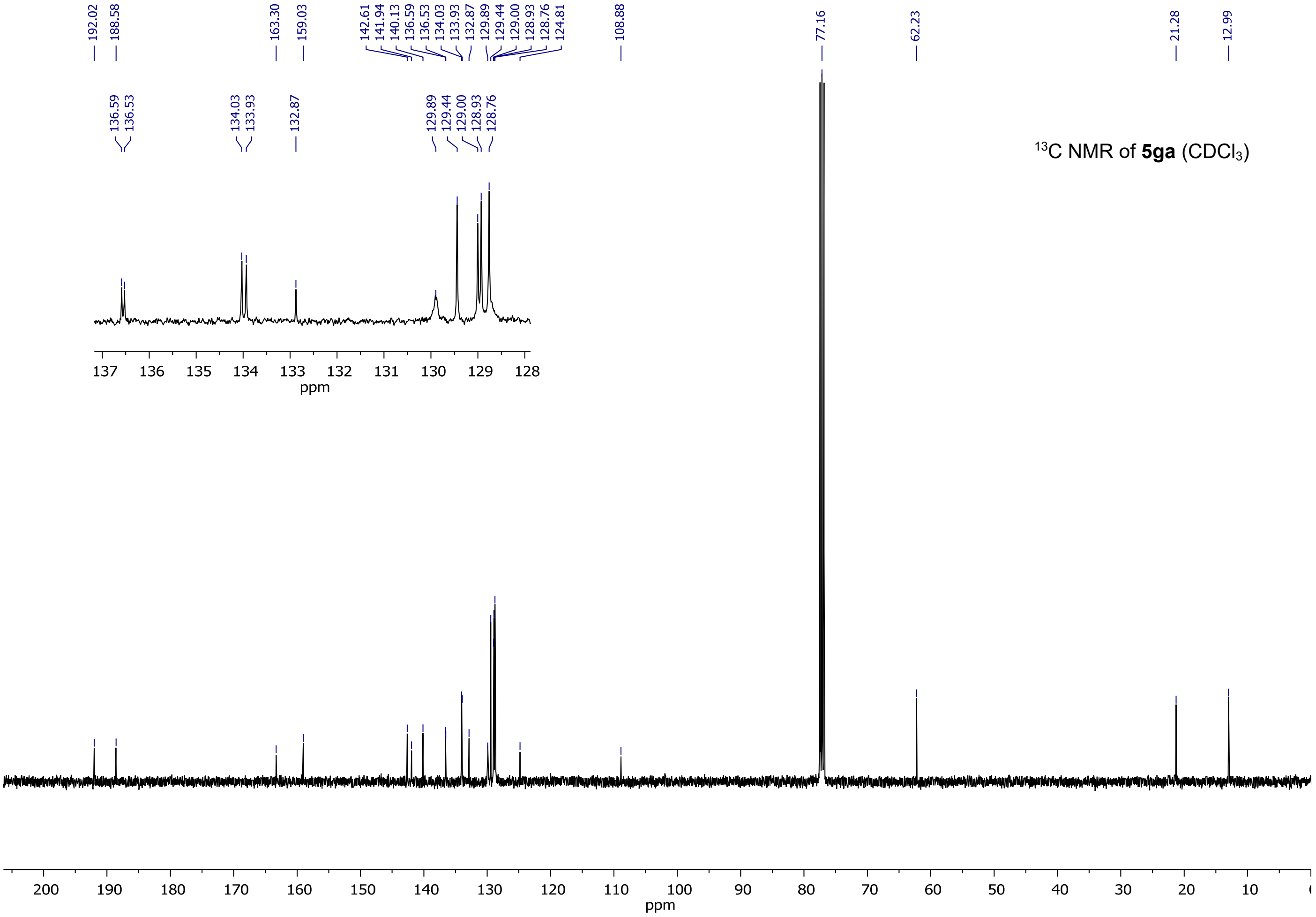
13C NMR of 5ga (CDCl3)
ppm

References:

1. Muranova, M. M. et al. Cyclopropanation vs. single-carbon insertion of pyrrole-2, 3-diones with sulfonium ylides: synthesis of functionalized 2-azabicyclo [3.1.0] hexanes and pyridine-2,3-diones. *Organic Chemistry Frontiers*, **2025**, *12*(7), 2187
2. Katritzky, A. R. et al. J. Preparation of tetrahydroindolizines from pyridinium and isoquinolinium ylides. *Journal of the Chemical Society, Perkin Transactions 1*, **1981**, 1180
3. Delaine, T. et al.Synthesis of the isonicotinoylnicotinamide scaffolds of the naturally occurring isoniazid− NAD (P) adducts. *The Journal of Organic Chemistry*, **2007**, *72*(2), 675
4. Caneschi W. et al. Synthesis and anticancer evaluation of new lipophilic 1, 2, 4 and 1, 3, 4-oxadiazoles. *European journal of medicinal chemistry*, **2019**, 165, 18
5. *CrysAlisPro*, Version 1.171.42.74a, Rigaku Oxford Diffraction, **2022**.
6. Dolomanov O.V., Bourhis L.J., Gildea R.J., Howard J.A.K., Puschmann H. *J. Appl. Cryst.*, **2009**, *42*, 339.
7. Sheldrick G.M. *Acta Crystallogr., Sect. A: Found. Adv.*, **2015**, *71*, 3.
8. Sheldrick G.M. *Acta Crystallogr., Sect. C: Struct. Chem.*, **2015**, *71*, 3.
